# Enabling Cysteine‐Free Native Chemical Ligation at Challenging Junctions with a Ligation Auxiliary Capable of Base Catalysis

**DOI:** 10.1002/anie.202107158

**Published:** 2021-07-29

**Authors:** Olaf Fuchs, Sebastian Trunschke, Hendrik Hanebrink, Marc Reimann, Oliver Seitz

**Affiliations:** ^1^ Department of Chemistry Humboldt-Universität zu Berlin Brook-Taylor-Str. 2 12489 Berlin Germany; ^2^ Department of Chemistry Technische Universität Berlin Straße des 17. Juni 135 10623 Berlin Germany

**Keywords:** amides, auxiliaries, base catalysis, peptide ligation, protein synthesis

## Abstract

Ligation auxiliaries are used in chemical protein synthesis to extend the scope of native chemical ligation (NCL) beyond cysteine. However, auxiliary‐mediated ligations at sterically demanding junctions have been difficult. Often the thioester intermediate formed in the thiol exchange step of NCL accumulates because the subsequent S→N acyl transfer is extremely slow. Here we introduce the 2‐mercapto‐2‐(pyridin‐2‐yl)ethyl (MPyE) group as the first auxiliary designed to aid the ligation reaction by catalysis. Notably, the MPyE auxiliary provides useful rates even for junctions containing proline or a β‐branched amino acid. Quantum chemical calculations suggest that the pyridine nitrogen acts as an intramolecular base in a rate‐determining proton transfer step. The auxiliary is prepared in two steps and conveniently introduced by reductive alkylation. Auxiliary cleavage is induced upon treatment with TCEP/morpholine in presence of a Mn^II^ complex as radical starter. The synthesis of a de novo designed 99mer peptide and an 80 aa long MUC1 peptide demonstrates the usefulness of the MPyE auxiliary.

## Graphical abstract

The MPyE group is the first sulfur based auxiliary enabling peptide ligation at proline and β‐branched amino acids. The pyridine nitrogen probably acts as a base that catalyzes the S→N acyl transfer during native chemical ligation.

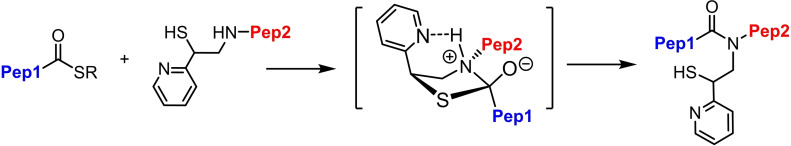

## Introduction

**Scheme 1:**
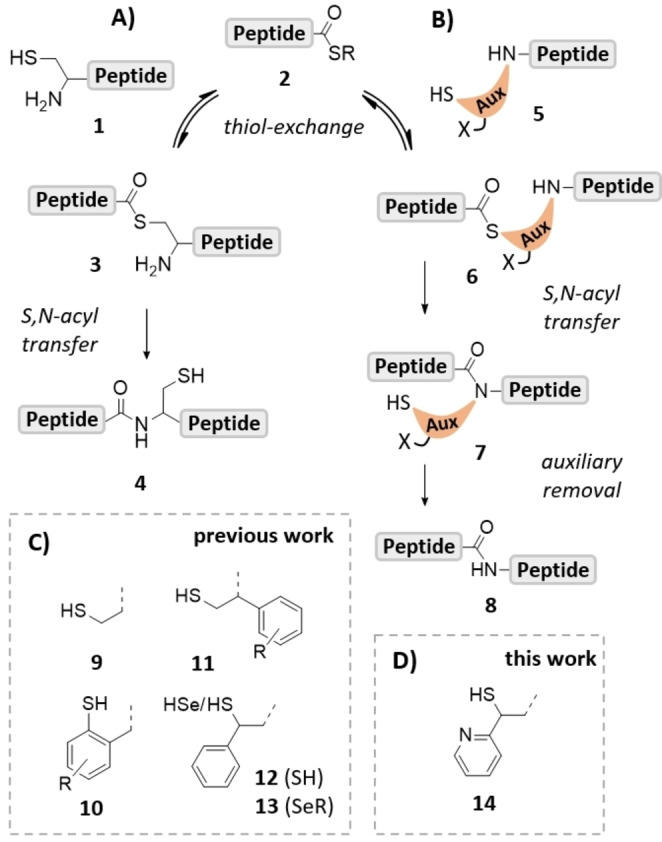
Mechanism of A) NCL and B) auxiliary‐mediated peptide ligation. Structure of C) reported auxiliaries and D) the new 2‐mercapto‐2‐(pyridin‐2‐yl)ethyl‐(MPyE)‐auxiliary.

The total chemical synthesis of proteins typically involves the solid phase assembly of unprotected peptide segments which are joined in aqueous buffers via native chemical ligation (NCL) reactions.^[1]^ NCL relies on the attack of an N‐terminal cysteine residue in **1** at the C‐terminal peptide thioester **2** (Scheme 1 A). The initially formed thioester intermediate **3** swiftly rearranges to form the peptide bond **4**. However, there are proteins that lack cysteine. Furthermore, cysteine residues might be inconveniently located e.g., close to terminal ends of the targeted protein. Several strategies exist to extend the scope of NCL chemistry to ligation junctions beyond cysteine.[^1c^, ^2^]

One such strategy relies on a sequence of ligation and desulfurization reactions. Initially, cysteine was used and converted to alanine by metal‐mediated desulfurization after NCL.^[3]^ The advent of the more robust radical desulfurization^[4]^ stimulated the development of a host of mercapto‐functionalized amino acids, which need to be prepared in suitably protected form prior to their coupling in solid phase peptide synthesis.^[1c]^ According to a potentially more convenient approach, a cleavable ligation auxiliary is coupled to the N‐terminal residue of the peptide destined to react in a NCL with a C‐terminal partner (Scheme 1 B).^[5]^ In theory, a single ligation auxiliary should be sufficient to access arbitrary ligation junctions.

The initially used ligation auxiliaries are based on N‐benzyl units, which scaffold the mercapto group in a position allowing the S→N acyl rearrangement to proceed via a 5‐ or 6‐membered transition state. For example, 2‐mercaptobenzyl‐type auxiliaries (**10**) and congeners with electron donating aryl substituents are cleavable under acidic conditions but useful ligation rates can only be achieved at glycine‐containing junctions.^[6]^ Ligations at 1‐aryl‐2‐mercaptoethyl auxiliaries (**11**) can proceed through the preferred 5‐membered transition state but the steric bulk introduced in α‐position to the reactive amine limits applications, again, to ligations at glycine.^[7]^ We explored different auxiliary architectures^[8]^ and with the introduction of the 2‐mercapto‐2‐phenethyl (MPE) auxiliary^[9]^ **12**, we have changed the N‐benzyl paradigm. Moving the phenyl substituent from the α‐ to the β‐position decreases the steric demand in vicinity of the reactive amine. The MPE auxiliary permits NCL reactions at non‐glycine containing junctions such as Ala‐Asn, Leu‐Asn and enabled the ligation with sterically more demanding amino acid residues such as Arg, Met and Gln. After the ligation, removal of the auxiliary is achieved through a radical fragmentation reaction.^[8b]^ However, sterically hindered junctions containing β‐branching amino acids were still out of reach. Recently, Wang et al. introduced a seleno version, the 2‐seleno‐2‐phenethyl auxiliary (**13**).^[10]^ As expected for reactions based on selenol exchange of selenoesters,^[11]^ this auxiliary allowed rapid ligation rates.

**Scheme 2:**
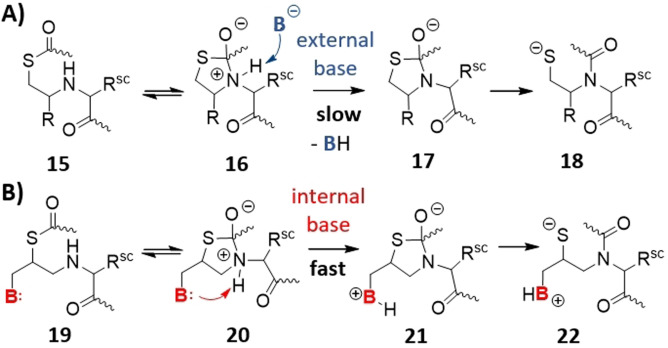
Proton transfer steps during the S→N acyl shift in NCL reactions. A) Hypothetic mechanism explaining the slow rate of the S→N acyl shift in auxiliary‐mediated ligation. B) An internal base within the auxiliary may accelerate S→N acyl transfer.

With the desire to develop a thiol auxiliary that would allow peptide ligations at arbitrary junctions, we looked more closely at the mechanism of auxiliary‐mediated native chemical ligation. A common observation is that ligation reactions at sterically demanding junctions afford the thioester‐linked intermediate **15** (Scheme 2 A) but the subsequent acyl shift is slow or does not occur at all.[^5^, ^6^] We hypothesized that intramolecular addition of the amine in **15** to the thioester may still occur, given the high rate of 5‐exo‐trig cyclization, but that the further reaction is hindered. For the S→N acyl shift to proceed, the resulting zwitterionic intermediate **16** must undergo deprotonation which yields thiazolidine **17** (or its OH form) as a precursor of the amid **18**, because otherwise an energetically unfavourable N‐protonated amid would have to be formed from intermediate **16**. At sterically demanding ligation sites, the zwitterionic intermediate **16** may have only a short lifetime because back reaction to **15** is faster than deprotonation by an external base. To speed up deprotonation (and, therefore, enable the S→N acyl shift to occur) we envisioned to introduce an internal base, which will afford a high effective molarity (Scheme 2 B). After thiol exchange, the thioester intermediate **19** will undergo ring closure and form **20**, which will be followed by a rapid intramolecular deprotonation delivering thiazolidine **21**. Once this stage is reached, an energetically favoured process leads to the amid bond upon extrusion of the thiolate (**21**→**22**).

Herein, we describe the first design of a ligation auxiliary envisioned to enable intramolecular base catalysis of native chemical ligation. We introduce the 2‐mercapto‐2‐(pyridine‐2‐yl)ethyl (MPyE) auxiliary and demonstrate its application in the synthesis of peptides via ligations at difficult junctions containing valine and proline. We also report quantum chemical calculations that illustrate the action of the pyridine base during the S→N acyl shift of a NCL reaction.

## Results and Discussion

**Figure 1:**
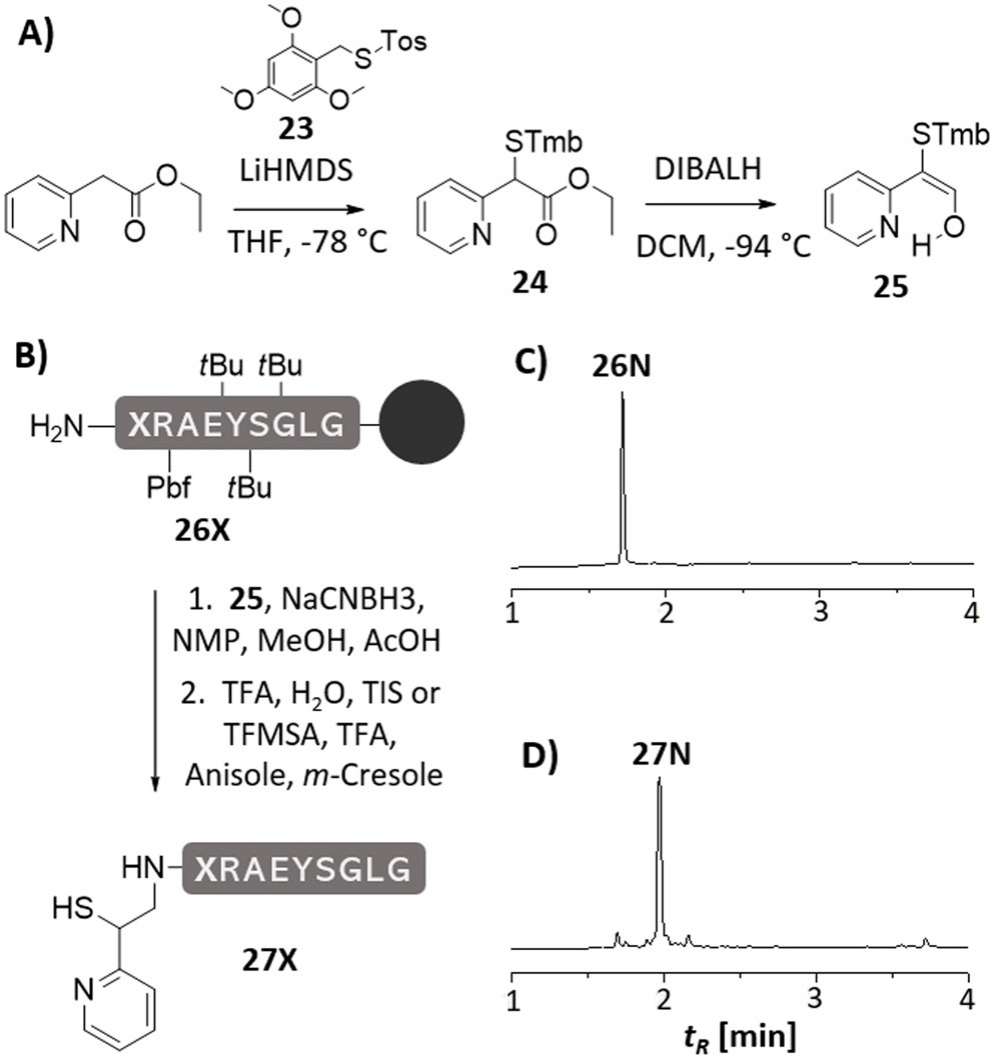
A) Synthesis of auxiliary building block **25** useful for B) introduction to resin‐bound peptides **26X** via reductive alkylation. Representative example of UPLC analysis of crude peptide **27N** (X=Asn) C) before and D) after reductive alkylation with **25**.

A suitable precursor of the 2‐mercapto‐2‐(pyridin‐2‐yl)ethyl auxiliary (**14**) was prepared in only two steps (Figure 1 A). Ethyl 2‐pyridylacetate was converted to an ester enolate and subsequently subjected to an electrophilic sulfurization with *S*‐2,4,6‐trimethoxybenzyl 4‐methylbenzenesulfonothioate (**23**).^[12]^ Reduction of the formed α‐mercapto ester **24** with diisobutylaluminium hydride (DIBALH) afforded **25** as a reagent ready for usage in reductive alkylation reactions. In contrast to previous aldehyde precursors used for auxiliary introduction,[^7b^, ^9^] compound **25** appears entirely in its enol form, which probably is stabilized by a hydrogen bond between the pyridine nitrogen and the alcohol. As a result, precursor **25** is shelf‐stable (Figure S5).

Next, we attached the MPyE auxiliary to the unprotected N‐terminus of resin‐bound peptides **26X** (Figure 1 B). Reductive alkylation with enol **25** and sodiumcyanoborohydride proceeded efficiently in polar solvents such as a mixture of MeOH and NMP (3:1) in presence of 2 % acetic acid. The auxiliary‐modified peptides were released by treatment with TFA/TIS/H_2_O (95/2.5/2.5). On the MPyE auxiliary, the *S*‐Tmb protecting group showed a comparably high acid stability, which may be due to a preferential protonation of the pyridine nitrogen hindering a second protonation at the thioether linkage. We extended the cleavage time to 24 h. For faster TMB removal, the peptide obtained upon standard TFA cleavage was treated with TFMSA/*m*‐cresol/thioanisol (7/1/1/1) for 2 h at 0 °C. The MPyE‐peptides **27X** showed high purity already in crude form, indicative of a smooth reductive alkylation reaction (see Figure 1 D, see also figures S16–S21, for details about storage: Suppl. Inf. 6.2).

**Figure 2:**
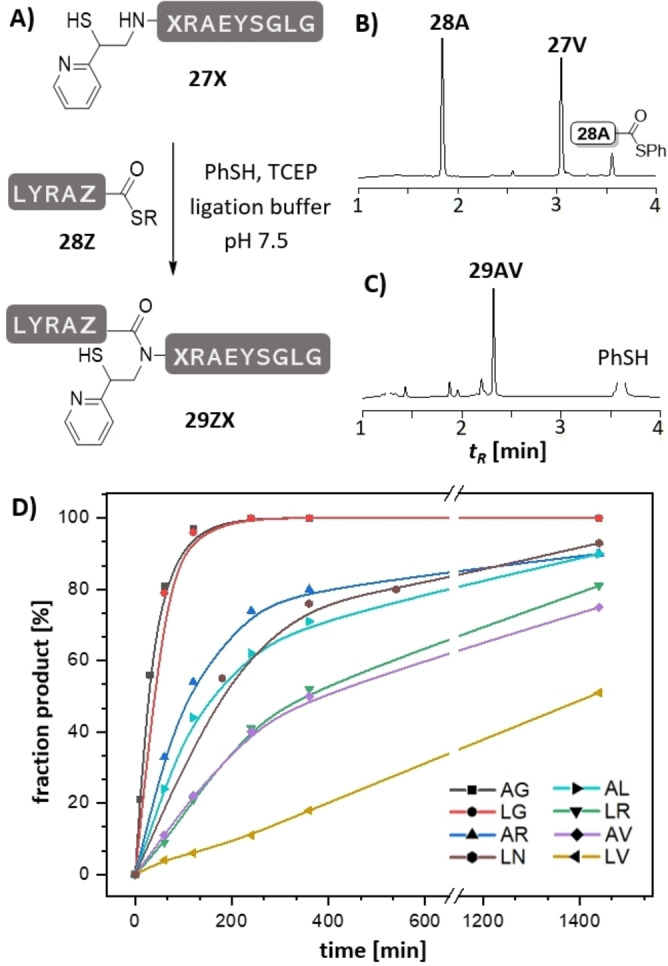
A) MPyE‐induced ligation between peptide thioesters **28Z** and peptides **27X** (letters Z and X represent amino acids specified in the Figure legend, R=(CH_2_)_2_CONHCH_2_CONH_2_). Representative UPLC analysis for the ligation of peptides **27V** and **28A** B) at *t*=1 min, C) after *t*=24 h and treatment with hydrazine (2.5 %). D) Time course of ligation at different junctions. Conditions: 5 mM peptides, 20 mM TCEP, 200 mM Na_2_HPO_4_, 6 M Gdn‐HCl, 3 vol. % PhSH, rt, pH 7.5.

To assess the MPyE auxiliary in NCL reactions, we examined the ligation of model peptides at nine different junctions (Figure 2, Table 1). As expected, reactions between alkylthioester **28A** or **28L** with the glycinyl peptide **27G** in presence of thiophenol presented little challenge and afforded the product in near quantitative yields. The reaction half time *t*_1/2_=25–40 min determined for ligation of 5 mM peptides is comparable to the speed of ligation with the MPE auxiliary. However, the enhanced reactivity of the MPyE auxiliary became apparent at sterically more hindered junctions. For example, reactions of alanine thioesters **28A** with peptides **27N**, **27R** and **27L** offering N‐terminal asparagine, arginine or leucine, respectively, furnished 90 % product after 24 h, with *t*_1/2_≈120–160 min. Even the ligation of **28A** with the sterically more demanding valine peptide **27V** occurred at useful rates (*t*_1/2_≈360 min). Also, Leu‐Asn and Leu‐Arg junctions were readily accessible. Furthermore, it was possible to establish the challenging Leu‐Val bond in reactions between **28L** and MPyE‐peptide **27V**. It should be emphasized that such junctions were inaccessible with previous ligation auxiliaries.

**Table 1:** Ligation yields, auxiliary cleavage yields and estimated reaction half times of MPyE induced peptide ligation.^[a]^

Ligation product	Ligation *t*_1/2_^[c]^	Ligation yield after HPLC purification^[a]^	Peptide after MPyE cleavage	Cleavage yield after HPLC purification^[b]^
**29AG**	≈25 min	82 %	**37AG**	88 %
**29LG**	≈40 min	78 %	**37LG**	54 %
**29AN**	≈120 min	78 %	**37AN**	55 %
**29LN**	≈160 min	72 %	**37LN**	39 %
**29AR**	≈120 min	73 %	**37AR**	47 %
**29LR**	≈360 min	66 %	**37LR**	58 %
**29AL**	≈160 min	42 %	**37AL**	30 %
**29AV**	≈360 min	58 %	**37AV**	22 %
**29LV**	≈24 h	51 %^[d]^	**37LV**	n.d.

[a] Conditions: see Figure 2. [b] Conditions: see Figure 5. [c] estimated from reaction time course in Figure 2 E). [d] fraction of product determined by UPLC analysis after 24 h.

The reactions studied by us involved the use of thiophenol as thiol additive. Though not studied yet, we believe that, based on the mechanism (vide infra), the MPyE auxiliary will provide for fast reactions also with other powerful thiol additives such as 4‐mercaptophenylacetic acid^[13]^ and 2,2,2‐trifluoroethanol.^[14]^

**Figure 3:**
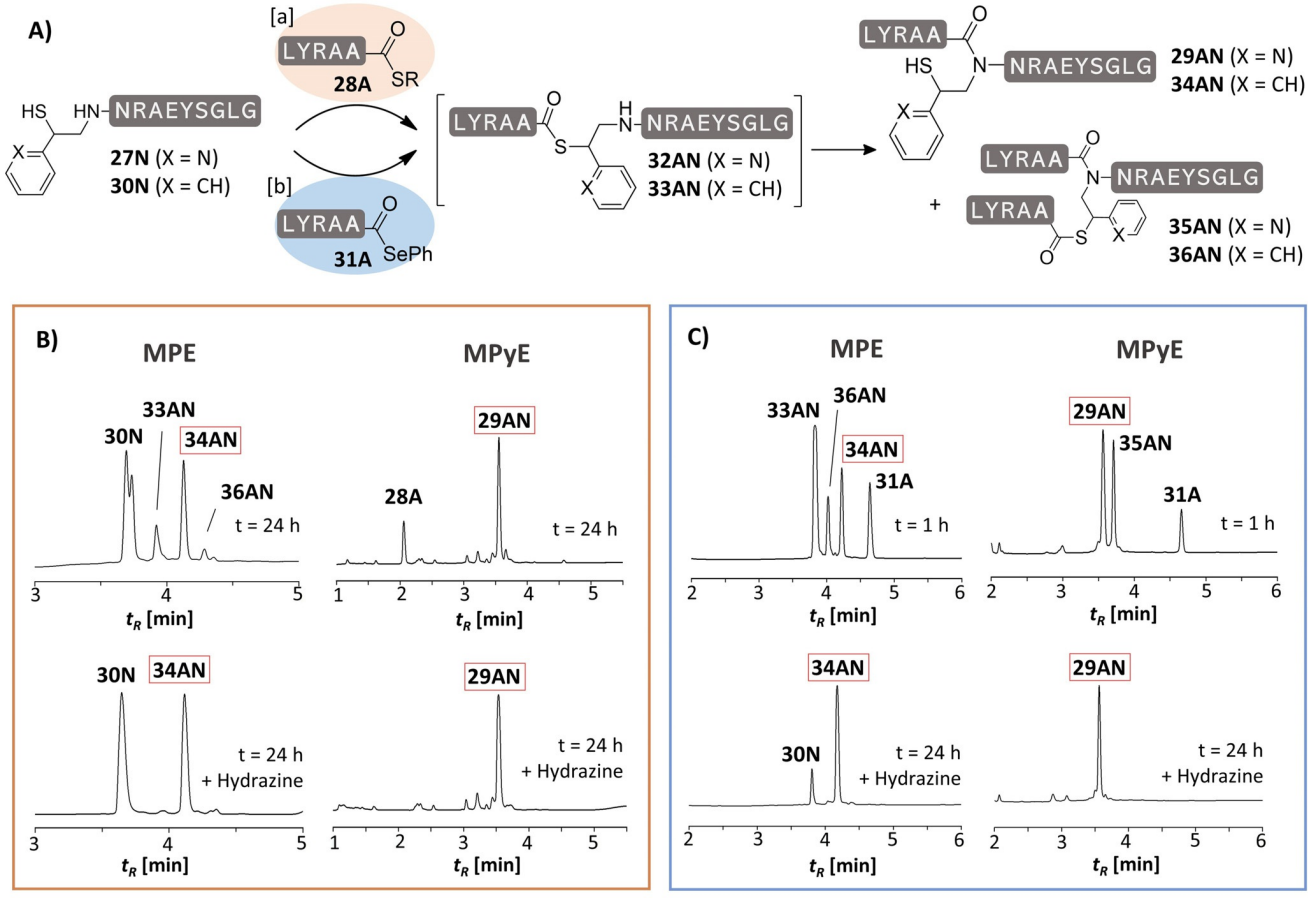
Comparison between peptide ligation on MPE and MPyE auxiliaries performed with peptide thioester **28A** (orange box) or peptide selenoester **31A** (blue box). UPLC analysis of aliquots from reactions with B) peptide thioester **28A** after 24 h or C) peptide selenoester **31A** after 1 h and 24 h. Hydrazine (2.5 %), if added, was used to cleave thioester‐linked conjugates such as **32AN**, **33AN**, **35AN** and **36AN**. Conditions: [a] 5 mM peptides, 20 mM TCEP, 200 mM Na_2_HPO_4_, 6 M Gdn‐HCl, 3 vol % PhSH, rt, pH 7.5 [b] 5 mM, 100 mM TCEP, 200 mM Na_2_HPO_4_, 6 M Gdn‐HCl, 28 mM DPDS, rt, pH 6.2.

A comparison between the MPyE and the MPE auxiliaries illustrates the effect of the pyridine residue (Figure 3 A). UPLC analysis revealed the existence of the thioester intermediate **33AN** when the MPE auxiliary was used for an Ala‐Asn ligation (Figure 3 B, left). After 24 h, a significant portion of the MPE‐Asn‐peptide **30N** was still not converted. By contrast, conversion of the MPyE‐Asn‐peptide **27N** was complete and UPLC analysis showed no trace of a thioester intermediate (Figure 3 B, right). In a next set of experiments, we used peptide selenoesters instead of thioesters to allow for an extremely rapid formation of the thioester intermediate.^[15]^ Under these conditions differences in the S→N acyl transfer rates become clearly apparent. The selenoesters were added in twofold excess to avoid scavenging of selenoesters by the auxiliary mercapto group of the formed ligation product. With the MPyE auxiliary, product formation was completed within one hour only (Figure 3 C, right). After this time, ligation on the MPE auxiliary mainly formed the thioester intermediate **33AN** (Figure 3 C, left). Of note, this intermediate was not observed with the MPyE auxiliary. Also, in reactions of leucine selenoester with MPyE‐Asn and MPyE‐Val peptides, the thioester intermediate did not accumulate (Figure S32–S33). In these investigations we observed that two isomers of MPE‐peptides can be formed at different rates, suggesting that the MPE enantiomers react differently (Figure S32B vs. S32C). Such effects were not observed with the MPyE auxiliary (Figure S32D–F).

Testing the limits of NCL chemistry, we also examined ligations involving C‐terminal proline which is known for its particularly low reactivity.^[16]^ Remarkably, the reaction of the proline selenoester with a MPyE‐peptide afforded the Pro‐Arg ligation product (Figure S34). Selenoester ligations are performed at slightly acidic conditions (pH 6.2).^[15]^ Under these conditions, the MPyE auxiliary is cleaved gradually from the peptide before ligation, which is notable when ligations proceed slowly, for example at the Leu‐Val (Figure S33) and the Pro‐Arg junction (Figure S34). Sluggish ligation reactions can furthermore be accompanied by desulfurization reactions (Figure S33, S34). Regardless of the side reactions occurring during selenoester ligation at the most challenging junctions, the experiments clearly illustrate that the MPyE auxiliary facilitates S→N acyl transfer.

During the course of our investigations of the MPyE auxiliary, Kanai et al. reported that coupling of a 2‐mercaptomethyl‐4‐dialkylaminopyridine (a DMAP derivative) to a nucleosome ligand guided acylation reactions to histone side chains.^[17]^ In a follow‐up paper, Otaka et al. described the use of 2‐mercaptomethyl‐4‐dimethylaminopyridine as a thiol additive for NCL chemistry.^[18]^ Though not used as a cleavable ligation auxiliary, the 2‐mercaptomethyl‐DMAP systems from Kanai et al. and Otaka et al. share a key feature with our MPyE auxiliary, that is, the 2‐mercaptomethylpyridine structure. Kanai and Otaka explained the beneficial effect of the 2‐mercaptomethyl‐DMAP systems with an increased acidity of the thiol group which would in turn increase the reactivity of the acyl component of the thioester intermediate formed upon thiol exchange. Considering that even NCL reactions on seleno‐modified auxiliaries can stop before rearrangement,^[10]^ we think that activation of the acyl component cannot account for the high ligation rates observed with the MPyE auxiliary.

**Figure 4:**
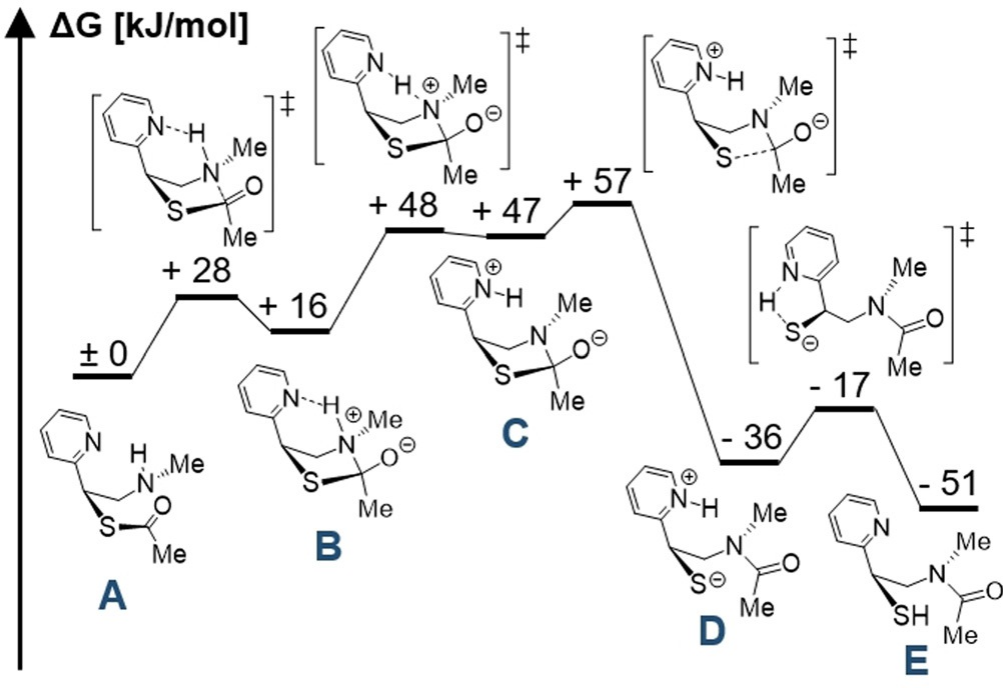
Calculated free energy surface for the S→N acyl transfer reaction for a model system. Free energies were calculated at the PBE0‐D4/TZ2P/3D‐RISM‐KH+PC+ level of theory using coupled cluster corrections to the final energies.

We investigated the elementary steps of the acyl transfer computationally at a PBE0‐D4/TZ2P level of theory for a minimal MPyE model system, starting from the thioester‐linked intermediate **A** (Figure 4). Hydration effects were described using a recent 3D‐RISM‐SCF implementation employing the exact electrostatic potential of the solute.^[19]^ The final energy calculations also include coupled cluster corrections calculated at the DLPNO‐CCSD(T)‐F12 level of theory (see SI for details). The resulting free energy surface indicates that the nitrogen of the pyridine ring acts as an intramolecular base, not only assisting in the formation of the zwitterionic intermediate **B** but also taking part in the deprotonation of the ammonium ion. This deprotonation is extremely facile. From **C**, a transition state leading to cleavage of the C−S bond in **D** is readily accessible. The resulting formation of the peptide bond is the thermodynamic driving force for the reaction. This mechanistic description is in very good agreement with the theoretical study of the S→N acyl transfer during a normal NCL from Wang et al.^[20]^ who find a free energy curve that is qualitatively very similar to ours. An additional intramolecular protonation of the sulfide anion in **D** by the pyridinium ion is possible and energetically feasible, but the involvement of the solvent cannot be excluded. Although the effect of sterically demanding junctions has not been investigated computationally, we propose the rate‐determining step to be fairly independent of the substituents, since it only involves an intramolecular proton transfer and a C−S bond cleavage. According to the quantum chemical calculations it seems plausible that pyridine facilitates S→N acyl transfer by base catalysis. Of note, Kemp et al. already discussed a possible contribution of intramolecular bases as catalysts of a proton transfer step in Kemp's thiol capture ligation, which involves an O→N acyl migration.^[21]^ Whether the known rapid reactions at histidine thioesters are caused by intramolecular base catalysis would have to be investigated in further studies.

**Figure 5:**
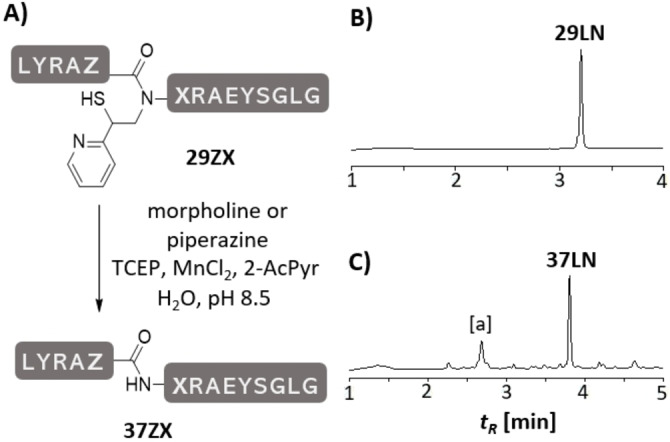
A) Removal of the MPyE auxiliary from ligation products **29ZX**. Representative UPLC analysis of MPyE cleavage of peptide **29LN** B) at *t*=0 h, C) after 20 h. [a] non‐peptidic material. Conditions: 2 M amine base*, 0.5 M TCEP, 0.5 M 2‐AcPyr, 5 mM MnCl_2_, rt, pH 8.5. *piperazine for X=G, morpholine for X≠G.

Cleavage of the MPyE auxiliary (Figure 5) was expected to proceed in presence of TCEP and an amine according to the radical fragmentation mechanism as previously described for the MPE auxiliary (see Figure S35 for a putative mechanism).^[8b]^ Reaction progress was slow when the peptides were exposed to conditions (1 M TCEP, 4 M morpholine, pH 8.5) optimized for removal of the MPE auxiliary. Interestingly, we observed that the rate of detachment depended on the source of the TCEP supply. We identified manganese as a beneficial impurity (Figure S53). Inspired by reports on Mn^III^‐induced radical reactions^[22]^ and considering that Mn^II^ salts in presence of organic ligands give Mn^III^ complexes upon oxidation by air, we explored the addition of Mn^II^ with different amine ligands. Highest yields were obtained by adding MnCl_2_ and 2‐acetylpyridine to an aqueous solution containing 0.5 M TCEP and 2 M morpholine (X≠Gly) or piperazine (X=Gly). The MPyE removal from Gly at the Leu‐Gly and Ala‐Gly junctions was achieved in less than two hours in >50 % yield of isolated material (Table 1). Detachment from other amino acids required longer reaction times but was complete after 17 h. Trace amounts of side products, that is desulfurized and oxidized auxiliary peptides were formed. In a particular case (Ala‐Leu junction **29AL**, Figure S43), auxiliary removal was accompanied by formation of a small amount of N‐formyl peptide indicative of the reaction mechanism (Figure S43).

**Figure 6:**
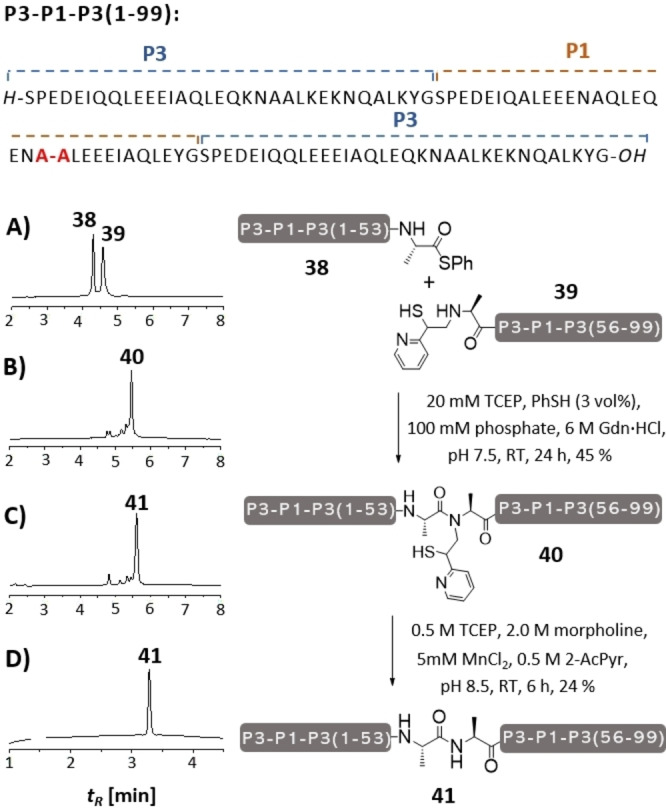
Synthesis of 99 aa peptide P3‐P1‐P3 (**41**) by ligation of 54 aa thioester **38** and 55 aa MPyE‐peptide **39** and auxiliary removal. UPLC analysis of aliquots withdrawn A) at the start of the ligation, B) after 24 h ligation and treatment with 2.5 % hydrazine (to confirm completion of S→N acyl transfer), C) auxiliary removal from purified ligation product and D) of purified **41**.

To demonstrate its versatility, the MPyE auxiliary was used in the synthesis of a 99 amino acid long P3‐P1‐P3 peptide (Figure 6) consisting of a sequence of the orthogonal coiled coil peptides P1 and P3, recently introduced by Jerala.^[23]^ The design emerged from our recent research activities,^[24]^ in which we explore coiled coil peptides as a means to control the labelling and, potentially, oligomerization of membrane receptors. For example, P3‐P1‐P3 may serve as a landing hub for the binding of a P2 peptide (via formation of a P1‐P2 coiled coil) and two P4 peptides (via P3‐P4 coiled coils). However, we noticed that extended coiled coil peptides are difficult to synthesize, mostly because their tendency to form aggregates hampers purification. For the synthesis of P3‐P1‐P3 we chose an Ala‐Ala ligation site within the central P1 unit. The SPPS of the N‐terminal segment (1–54) was performed on a chlorotrityl tentagel resin. Mild acid cleavage furnished the protected peptide acid which was converted to the phenyl thioester **38** upon reaction with diisopropylcarbodiimide (DIC) and thiophenol and subsequent treatment with TFA/H_2_O/TIS.^[25]^ The synthesis of the C‐terminal segment (55–99), auxiliary peptide **39**, involved chain assembly on a chlorotrityl tentagel resin and the introduction of the MPyE auxiliary, which was carried out via reductive alkylation with **25** as described. Purification of both peptide thioester **38** and auxiliary peptide **39** turned out to be challenging due to gel formation at concentrations >1 mM. For ligation, 6 M guanidinium hydrochloride was added to prevent gelation. UPLC analysis showed that the two peaks for the starting materials **38** and **39** converged to a single peak within 24 h (Figure 6). Treatment with hydrazine verified that the new peak formed upon auxiliary‐mediated NCL at the Ala‐Ala junction is due to an amid bond linkage indicating that the S→N acyl shift proceeded also in this case. After HPLC purification, the MPyE auxiliary was cleaved by treatment of **40** with the aforementioned removal mixture. Detachment was complete after 6 h incubation.

**Figure 7:**
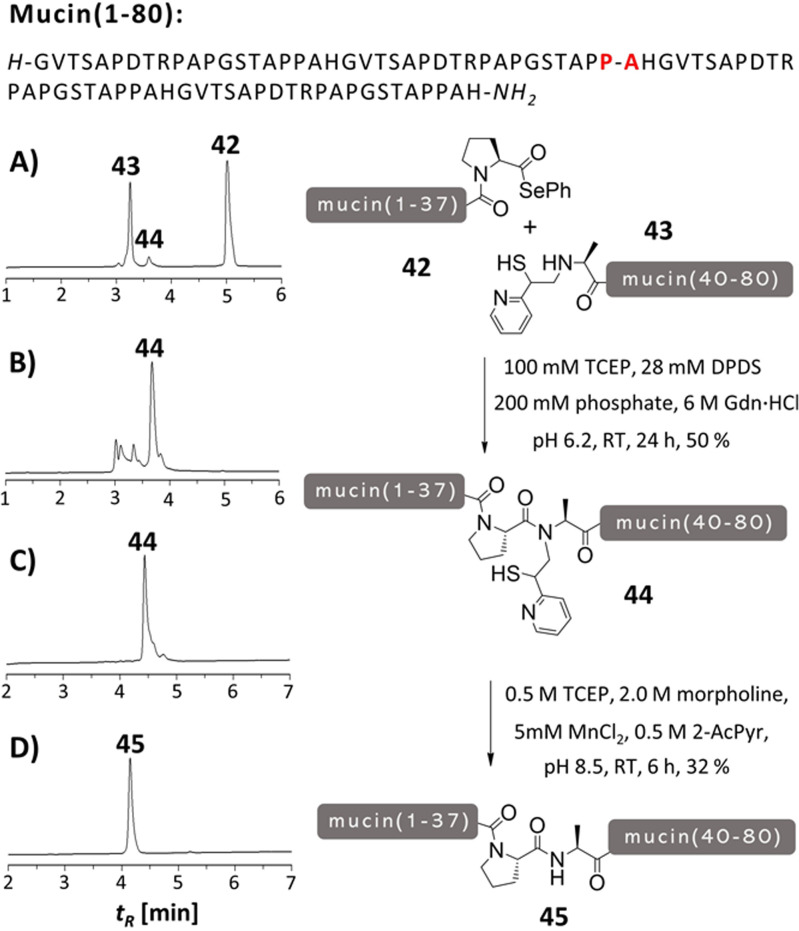
Synthesis of a MUC1 peptide **45** by Pro‐Ala ligation involving peptide selenoester **42** with MPyE‐peptide **43** followed by auxiliary cleavage. UPLC analysis of aliquots withdrawn A) at the start of the ligation, B) after 24 h ligation and treatment with 2.5 % hydrazine (to confirm completion of S→N acyl transfer), C) auxiliary removal from purified ligation product and D) of purified **45**.

In a second application example, we applied the MPyE auxiliary to the synthesis of a MUC1 protein composed of 4 copies of 20mer tandem repeat sequence (Figure 7).^[26]^ MUC1 lacks cysteine to enable conventional NCL reaction. This and the exceptionally high proline content of 25 % prompted us to consider a proline ligation. Though a Pro‐Gly junction would be available, we opted to explore the MPyE auxiliary in a more difficult Pro‐Ala junction. To compensate for the weak reactivity of proline esters, we prepared the selenoester **42** (1–38) by using the method described by Payne et al.^[27]^ The C‐terminal coupling partner, the auxiliary peptide **43** (39–80), was accessed by reductive alkylation of the peptide assembled on a Rink Amid Tentagel Resin. The ligation reaction between proline selenoester **42** and the MPyE‐Ala‐peptide **43** proceeded smoothly without undesired loss of auxiliary that was observed in selenoester ligation of model peptides (Figure 7 B). HPLC analysis of an aliquot that was treated with hydrazine suggested that the S→N rearrangement had occurred also at the Pro‐Ala junction within an 80 aa peptide. HPLC purification after 24 h reaction time afforded the ligation product in 50 % yield. The ligation product **44** was incubated for 5 h with the pH 8.5 removal cocktail, which resulted in a clean detachment of the MPyE auxiliary (Figure 7 C).

## Conclusion

The design of the cleavable ligation auxiliaries used in the past followed two main criteria; to arrange the mercapto group in a position that enables the transfer of the captured acyl component to the amino group, preferably via 5‐ or 6‐membered ring intermediates, and to allow cleavage after ligation. Becker et al. were amongst the first to expand the functionality of an auxiliary by adding a solubility enhancing oligoethylene glycol chain.^[28]^ In this report, we introduce a first of its kind 2‐mercapto‐2‐(pyridin‐2‐yl)ethyl (MPyE) auxiliary that provides additional functionality for the catalysis of native chemical ligation. Based on the results of quantum chemical calculations the pyridine unit serves as a base that accelerates the native chemical ligation by facilitating the S→N acyl transfer step. Confirming this notion, the experimental data show that, in contrast to reactions on previously published systems, MPyE‐mediated NCL at sterically hindered junctions does not result in accumulation of the thioester intermediate. Our data shows that the MPyE unit is, in fact, the first thiol ligation auxiliary enabling amid bond formation at junctions containing prolin or β‐branched amino acids. A precursor for usage in solid‐phase synthesis is readily available in two steps only. The introduction of the MPyE auxiliary is conveniently performed by reductive alkylation of the resin‐bound peptide. For ligation, both peptide thioesters and peptide selenoesters can be used, though care has to be taken to avoid losses of the auxiliary during the mildly acidic conditions of the selenoester method.

For removal of the MPyE auxiliary via a radical‐induced fragmentation reaction, the ligation products were treated with TCEP/morpholine under slightly basic conditions (pH 8.5) in presence of oxygen, as described for the MPE auxiliary. We found that addition of a Mn^II^ complex speed up cleavage, presumably by facilitating initiation of the radical reaction. A comparison of the entries in Table 1 reveals that the MPyE auxiliary cleavage step is typically lower yielding than the ligation step. This is partly due to minor side reactions (desulfurization and oxidation of auxiliary, incomplete cleavage of N‐formyl peptide at hindered junctions), but also probably to the need to separate high amounts of phosphine products and base. However, this peculiarity does not affect the success of protein synthesis, as demonstrated by the successful synthesis of an 80 aa long MUC1 peptide via a selenoester ligation at a Pro‐Ala junction. The synthesis of a 99 aa long de novo designed peptide (P3‐P1‐P3) also proceeded smoothly.

With the MPyE ligation auxiliary we have presented the first example of a new paradigm. The auxiliary can actively participate in the ligation reaction revealing a novel path for future auxiliary design. Based on the ease of auxiliary introduction, the useful reaction rates at challenging ligation junctions and the mild cleavage conditions we believe that MPyE‐type auxiliaries have the potential to simplify chemical protein synthesis. In future work, we will introduce substituents at the MPyE ring and explore other basic heterocyles to further improve ligation rates and facilitate auxiliary cleavage.

## Conflict of interest

The authors declare no conflict of interest.

## Supporting information

As a service to our authors and readers, this journal provides supporting information supplied by the authors. Such materials are peer reviewed and may be re‐organized for online delivery, but are not copy‐edited or typeset. Technical support issues arising from supporting information (other than missing files) should be addressed to the authors.

Supporting Information

## References

[1a] P. E. Dawson, T. W. Miur, I. Clark-Lewis, S. B. H. Kent, Science 1994, 266, 776–779; 7973629 10.1126/science.7973629

[1b] S. Kent, Bioorg. Med. Chem. 2017, 25, 4926–4937; 28687227 10.1016/j.bmc.2017.06.020

[1c] A. C. Conibear, E. E. Watson, R. J. Payne, C. F. W. Becker, Chem. Soc. Rev. 2018, 47, 9046–9068; 30418441 10.1039/c8cs00573g

[1d] S. Bondalapati, M. Jbara, A. Brik, Nat. Chem. 2016, 8, 407–418. 27102674 10.1038/nchem.2476

[2] V. Agouridas, O. El Mahdi, V. Diemer, M. Cargoët, J.-C. M. Monbaliu, O. Melnyk, Chem. Rev. 2019, 119, 7328–7443. 31050890 10.1021/acs.chemrev.8b00712

[3] L. Z. Yan, P. E. Dawson, J. Am. Chem. Soc. 2001, 123, 526–533. 11456564 10.1021/ja003265m

[4a] Q. Wan, S. J. Danishefsky, Angew. Chem. Int. Ed. 2007, 46, 9248–9252; 10.1002/anie.200704195 18046687

[4b] C. Haase, H. Rohde, O. Seitz, Angew. Chem. Int. Ed. 2008, 47, 6807–6810; 10.1002/anie.200801590 18626881

[5] L. E. Canne, S. J. Bark, S. B. Kent, J. Am. Chem. Soc. 1996, 118, 5891–5896.

[6] J. Offer, C. N. C. Boddy, P. E. Dawson, J. Am. Chem. Soc. 2002, 124, 4642–4646. 11971712 10.1021/ja016731w

[7a] P. Botti, M. R. Carrasco, S. B. H. Kent, Tetrahedron Lett. 2001, 42, 1831–1833;

[7b] D. Macmillan, D. W. Anderson, Org. Lett. 2004, 6, 4659–4662. 15575654 10.1021/ol048145o

[8a] Z. Harpaz, S. Loibl, O. Seitz, Bioorg. Med. Chem. Lett. 2016, 26, 1434–1437; 26838809 10.1016/j.bmcl.2016.01.060

[8b] S. F. Loibl, A. Dallmann, K. Hennig, C. Juds, O. Seitz, Chem. Eur. J. 2018, 24, 3623–3633. 29334413 10.1002/chem.201705927

[9] S. F. Loibl, Z. Harpaz, O. Seitz, Angew. Chem. Int. Ed. 2015, 54, 15055–15059; 10.1002/anie.201505274 26545341

[10] H. Yin, D. Lu, S. Wang, P. Wang, Org. Lett. 2019, 21, 5138–5142. 31247759 10.1021/acs.orglett.9b01737

[11] S. S. Kulkarni, E. E. Watson, B. Premdjee, K. W. Conde-Frieboes, R. J. Payne, Nat. Protoc. 2019, 14, 2229–2257. 31227822 10.1038/s41596-019-0180-4

[12] R. E. Thompson, B. Chan, L. Radom, K. A. Jolliffe, R. J. Payne, Angew. Chem. Int. Ed. 2013, 52, 9723–9727; 10.1002/anie.201304793 23893778

[13] E. C. B. Johnson, S. B. H. Kent, J. Am. Chem. Soc. 2006, 128, 6640–6646. 16704265 10.1021/ja058344i

[14] R. E. Thompson, X. Liu, N. Alonso-García, P. J. B. Pereira, K. A. Jolliffe, R. J. Payne, J. Am. Chem. Soc. 2014, 136, 8161–8164. 24873761 10.1021/ja502806r

[15] T. Durek, P. F. Alewood, Angew. Chem. Int. Ed. 2011, 50, 12042–12045; 10.1002/anie.201105512 21997950

[16] S. B. Pollock, S. B. H. Kent, Chem. Commun. 2011, 47, 2342–2344. 10.1039/c0cc04120c 21173985

[17] Y. Amamoto, Y. Aoi, N. Nagashima, H. Suto, D. Yoshidome, Y. Arimura, A. Osakabe, D. Kato, H. Kurumizaka, S. A. Kawashima, K. Yamatsugu, M. Kanai, J. Am. Chem. Soc. 2017, 139, 7568–7576. 28534629 10.1021/jacs.7b02138

[18] K. Ohkawachi, D. Kobayashi, K. Morimoto, A. Shigenaga, M. Denda, K. Yamatsugu, M. Kanai, A. Otaka, Org. Lett. 2020, 22, 5289–5293. 32396369 10.1021/acs.orglett.0c01383

[19] M. Reimann, M. Kaupp, J. Phys. Chem. A 2020, 124, 7439–7452. 32838530 10.1021/acs.jpca.0c06322

[20] C. Wang, Q.-X. Guo, Y. Fu, Chem. Asian J. 2011, 6, 1241–1251. 21365769 10.1002/asia.201000760

[21] D. S. Kemp, R. I. Carey, J. Org. Chem. 1993, 58, 2216–2222.

[22] F. Paoletti, A. Mocali, D. Aldinucci, Chem.-Biol. Interact. 1990, 76, 3–18. 2168295 10.1016/0009-2797(90)90030-q

[23] H. Gradišar, R. Jerala, J. Pept. Sci. 2011, 17, 100–106. 21234981 10.1002/psc.1331

[24a] K. Gröger, G. Gavins, O. Seitz, Angew. Chem. Int. Ed. 2017, 56, 14217–14221; 10.1002/anie.201705339 28913864

[24b] A. S. Eklund, M. Ganji, G. Gavins, O. Seitz, R. Jungmann, Nano Lett. 2020, 20, 6732–6737; 32787168 10.1021/acs.nanolett.0c02620 PMC7496730

[24c] G. C. Gavins, K. Gröger, M. D. Bartoschek, P. Wolf, A. G. Beck-Sickinger, S. Bultmann, O. Seitz, Nat. Chem. 2021, 13, 15–23; 33288896 10.1038/s41557-020-00584-z

[24d] U. Reinhardt, J. Lotze, S. Zernia, K. Mörl, A. G. Beck-Sickinger, O. Seitz, Angew. Chem. Int. Ed. 2014, 53, 10237–10241; 10.1002/anie.201403214 25081195

[25a] S. Futaki, K. Sogawa, J. Maruyama, T. Asahara, M. Niwa, H. Hojo, Tetrahedron Lett. 1997, 38, 6237–6240;

[25b] R. von Eggelkraut-Gottanka, A. Klose, A. G. Beck-Sickinger, M. Beyermann, Tetrahedron Lett. 2003, 44, 3551–3554.

[26] J. Taylor-Papadimitriou, J. Burchell, D. W. Miles, M. Dalziel, Biochim. Biophys. Acta Mol. Basis Dis. 1999, 1455, 301–313. 10.1016/s0925-4439(99)00055-1 10571020

[27] N. J. Mitchell, L. R. Malins, X. Liu, R. E. Thompson, B. Chan, L. Radom, R. J. Payne, J. Am. Chem. Soc. 2015, 137, 14011–14014. 26487084 10.1021/jacs.5b07237

[28] C. Bello, C. F. W. Becker, Bioorg. Med. Chem. 2017, 25, 5016–5021. 28579308 10.1016/j.bmc.2017.05.046

