# Supplementary material for: Enabling Cysteine‐Free Native Chemical Ligation at Challenging Junctions with a Ligation Auxiliary Capable of Base Catalysis
Source: Angew Chem Int Ed Engl. 2021 Jul 29;60(35):19483–90. doi: 10.1002/anie.202107158 (PMC8457107; doi:10.1002/anie.202107158)
Supplement: Supplementary file 1 — Supporting Information [file ANIE-60-19483-s001.pdf]

Angewandte Chemie
Eine Zeitschrift der Gesellschaft Deutscher Chemiker

Supporting Information

Enabling Cysteine-Free Native Chemical Ligation at Challenging Junctions with a Ligation Auxiliary Capable of Base Catalysis

*Olaf Fuchs+, Sebastian Trunschke+, Hendrik Hanebrink, Marc Reimann, and Oliver Seitz**

anie_202107158_sm_miscellaneous_information.pdf

Table of Contents

1 GENERAL INFORMATION 3

1.1 MATERIALS AND INSTRUMENTS 3

2 SYNTHESIS OF AUXILIARY PRECURSOR 4

3 NMR SPECTROSCOPY 6

4 SYNTHESIS OF PEPTIDES 11

4.1 FMOC-STRATEGY 11
4.2 BOC-STRATEGY FOR THE SYNTHESIS OF PEPTIDE THIOESTER 12
4.3 DETERMINATION OF PEPTIDE AND PROTEIN CONCENTRATION 13

5 SYNTHESIS OF PEPTIDE CHALKOESTER 14

5.1 PEPTIDE THIOESTER 14
5.2 PEPTIDE SELENOESTER 17

6 INTRODUCTION OF THE AUXILIARY 20

6.1 INTRODUCTION OF THE 2-MERCAPTO-2-(PYRIDIN-2-YL)ETHYL AUXILIARY DURING SPPS 20
6.2 STORAGE OF PURIFIED 2-MERCAPTO-2-(PYRIDIN-2-YL)ETHYL AUXILIARY PEPTIDES 26

7 NATIVE CHEMICAL LIGATION 27

7.1 GENERAL PROCEDURE FOR LIGATION OF MPYE-PEPTIDES WITH PEPTIDE THIOESTERS 27

8 COMPARISON BETWEEN MPYE- AND MPE-PEPTIDES IN LIGATION REACTIONS WITH PEPTIDE SELENOESTER 36

8.1 LIGATION OF MPYE-PEPTIDES WITH PEPTIDE SELENOESTERS 36

9 COMPARISON BETWEEN PROLINE SELENO- AND PROLINE THIOESTER IN LIGATION WITH A MPYE-PEPTIDE 40

10 AUXILIARY REMOVAL 41

10.1 GENERAL PROCEDURE FOR REMOVAL OF THE MPYE AUXILIARY 41

11 SYNTHESIS OF P3-P1-P3 51

12 SYNTHESIS OF MUC1 80MER 55

13 INDUCTIVELY COUPLED PLASMA ATOMIC EMISSION SPECTROSCOPY 59

14 SCOPE AND LIMITATIONS OF THE MPYE AUXILIARY 60

14.1 LIGATION AT DIFFICULT LIGATION SITES 60

15 QUANTUM CHEMICAL CALCULATION 61

16 REFERENCES 67

1 General Information

1.1 Materials and instruments

Commercially available compounds were used without further purification. Dry solvents were taken from a *MBraun* Solvent Purification System *SPS 800*. Purification of compounds by flash chromatography was done on silica gel (0.060-0.2 mm, 60 Å) from *Acros Organics* using technical grade solvents. TLC silica gel plates *60 F254* from *Merck* were used for thin-layer chromatography. NMR-spectra were recorded on a *Bruker Avance II* 500 MHz Spectrometer and referenced to the residual protonated solvent signal. Elemental analysis was carried out on a *HEKAtech Eurovector3000*.

Preparative HPLC purifications were carried out by using an *Agilent 1100 Series* system and a *Nucleodur C18 Gravity* column (250 mm x 21 mm, 5 µm) from *Macherey-Nagel* with a binary mixture of A (0.1 % TFA, 1 % ACN, 98.9 % H_2O) and B (0.1 % TFA, 1 % H_2O, 98.9 % ACN) as a mobile phase (flow = 15 mL/min) in a linear gradient as described. For semi-preparative HPLC-purifications a Polaris C18-A column (250 x 10.0 mm) from *Varian* was used with a binary mixture of A (0.1 % TFA, 1 % ACN, 98.9 % H_2O) and B (0.1 % TFA, 1 % H_2O, 98.9 % ACN) as mobile phase (flow = 6.0 mL/min) in a linear gradient as described.

UPLC-MS measurements were performed by using an *Acquity* system from *Waters* and a *BEH130* C18 column (2.1 x 50 mm, 1.7 µm; heater set on 50 °C) with a binary mixture of A (0.1 % TFA, 1 % ACN, 98.9 % H_2O) and B (0.1 % TFA, 1 % H_2O, 98.9 % ACN) as a mobile phase (flow = 0.5 mL/min) in a linear gradient as described.

High-resolution ESI-MS spectra were recorded on an *Agilent 6220 TOF Accurate Mass* coupled to an *Agilent 1200 LC* (*Agilent Technologies*, USA) and measured at 35 °C between 100 - 2000 m/z. An *Accucore RP-MS* (30 x 2.1 mm; 2.6 µm particle size) was used as stationary phase at a flow of 0.8 mL/min and the following gradient (A = water, B = acetonitrile): 95 % A + 5 % B for 0.2 min, then 95 % A + 5 % B to 1 % A + 99 % B to 1.1 min, then 1 % A + 99 % B to 2.5 min.

Inductively coupled plasma atomic emission spectroscopy (ICP-OES) analysis was performed using an *Optima 8300* optical emission spectrometer from *Perkin Elmer* in axial mode. For the evaluation, commercial multi-element standards (*Certipur*, *Merck*) were used. Data analysis was performed using the *Syngistix* software for ICP (*Perkin Elmer*).

2 Synthesis of auxiliary precursor

S-2,4,6-trimethoxybenzyl 4-methylbenzenesulfonothioate (23)

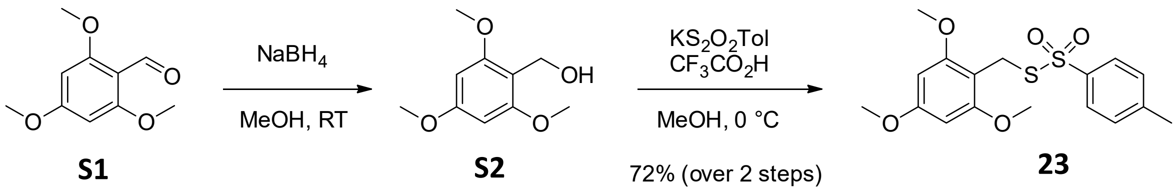

Alcohol **S2** and thiosulfonate **23** were synthesized according to a published procedure.[1] The NMR data obtained for **23** is in agreement with the literature data.

Ethyl 2-(pyridin-2-yl)-2-((2,4,6-trimethoxybenzyl)thio)acetate (24)

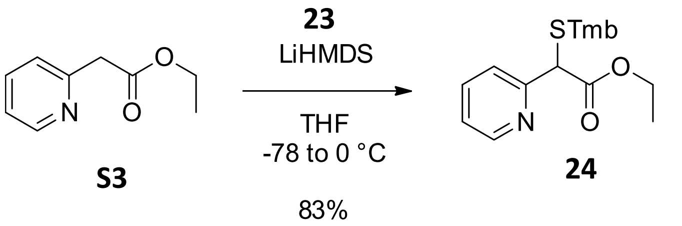

Under argon atmosphere, lithium hexamethyldisilazide (5.25 mL, 1.0 M in THF, 1.05 eq.) was added to a stirred solution of methyl 2-pyridylacetate (0.76 mL, 5.0 mmol) in 50 mL anhydrous THF over the course of 1 h at -78 °C. To this solution, thiosulfonate **23** (1.84 g, 5.0 mmol, 1.0 eq.) in 50 mL anhydrous THF was added at -78 °C over 1 h. After the reaction mixture was allowed to gradually warm to room temperature, H_2O was added. The resultant mixture was extracted with EtOAc (2x) and the combined organic layers were dried over $MgSO_4$, filtered, and concentrated under reduced pressure. The residue was purified by flash column chromatography on silica gel (hexane/EtOAc = 1:1 v/v) to afford **24** (1.56 g, 4.1 mmol) in 83 % yield as a white solid.

^{1}H NMR ($CDCl_3$, 500 MHz): δ [ppm] = 8.52 (d, J = 5.2, 1H), 7.64 (t, J = 7.8 Hz, 1H), 7.58 (d, J = 7.8 Hz, 1H), 7.15 (t, J = 5.2 Hz, 1H), 6.06 (s, 2H), 4.88 (s, 1H), 4.18 (q, J = 7.1 Hz, 2H), 3.89 (d, J = 12.3 Hz, 1H), 3.84 (d, J = 12.3 Hz, 1H), 3.78 (s, 3H), 3.76 (s, 6H), 1.24 (t, J = 7.1, 3H).
^{13}C NMR ($CDCl_3$, 126 MHz): δ [ppm] = 170.60, 160.63, 159.00, 157.10, 149.07, 136.76, 123.10, 122.53, 107.00, 90.56, 61.70, 55.80, 55.44, 54.90, 24.70, 14.20.

(*E*)-2-(pyridin-2-yl)-2-((2,4,6-trimethoxybenzyl)thio)ethanol (25)

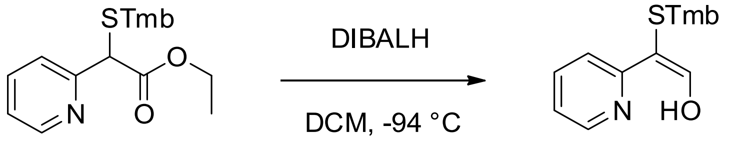

24 **25**

Under argon atmosphere ester **24** (1.55 g, 4.1 mmol) was dissolved in 20 mL anhydrous DCM and cooled to -94 °C. Diisobutylaluminium hydride (12.3 mL, 1.0 M in toluene, 3.0 eq.) was added carefully over the vessel wall over the course of 1 h. The reaction mixture was stirred for an additional 15 min at -94 °C. Subsequently, a mixture of DCM:MeOH (1 mL, 1:1 v/v) was added over the vessel wall over the course of 30 min at -94 °C. The reaction mixture was stirred at room temperature for 10 min and a solution of potassium sodium tartrate (20 mL saturated) was added. The resulting mixture was stirred at room temperature for 1 h and then extracted with DCM (3x). The combined organic layers were dried over $MgSO_4$, filtered and concentrated. The residue was purified by recrystallization (MeOH) to afford the enol **25** (1.12 g, 3.4 mmol, 82 %) as bright yellow crystals.

Elemental analysis: Anal. Calcd. for $C_{17}H_{19}NO_4S$: H, 5.74; C, 61.24; N, 4.20; S, 9.62. Found: H, 5.90; C, 61.56; N, 4.19; S, 9.47.

^{1}H NMR ($CDCl_3$, 500 MHz): δ [ppm] = 16.22 (s, 1H), 8.01 (ddd, J = 5.6, 1.7, 1.0 Hz, 1H), 7.89 (s, 1H), 7.78 (dt, J = 8.6, 1.0 Hz, 1H), 7.66 (ddd, J = 8.6, 7.1, 1.7 Hz, 1H), 6.93 (ddd, J = 7.1, 5.6, 1.0 Hz, 1H), 6.03 (s, 2H), 3.77 (s, 3H), 3.75 (s, 2H), 3.63 (s, 6H).

^{13}C NMR ($CDCl_3$, 126 MHz): δ [ppm] = 173.32, 160.39, 158.99, 139.93, 137.98, 121.54, 117.41, 107.42, 98.81, 90.44, 55.69, 55.43, 28.28.

Storage of Auxiliary Precursor

From the available NMR data, it can be concluded that the auxiliary precursor **25** is exclusively present in its enol form. The compound is stable and did not decompose over a period of 7 month (see figure S5).

3 NMR Spectroscopy

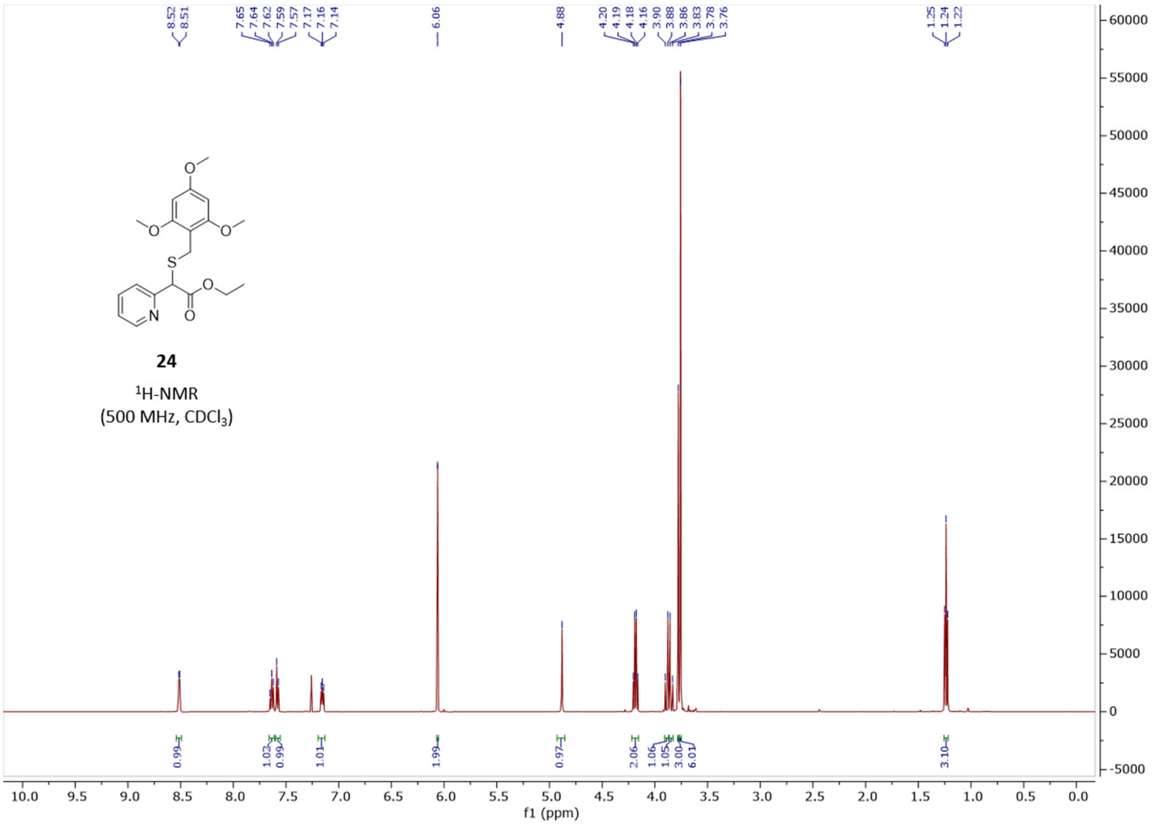

Figure S1: ^{1}H NMR spectrum (500 MHz) of ester **24** in $CDCl_3$.

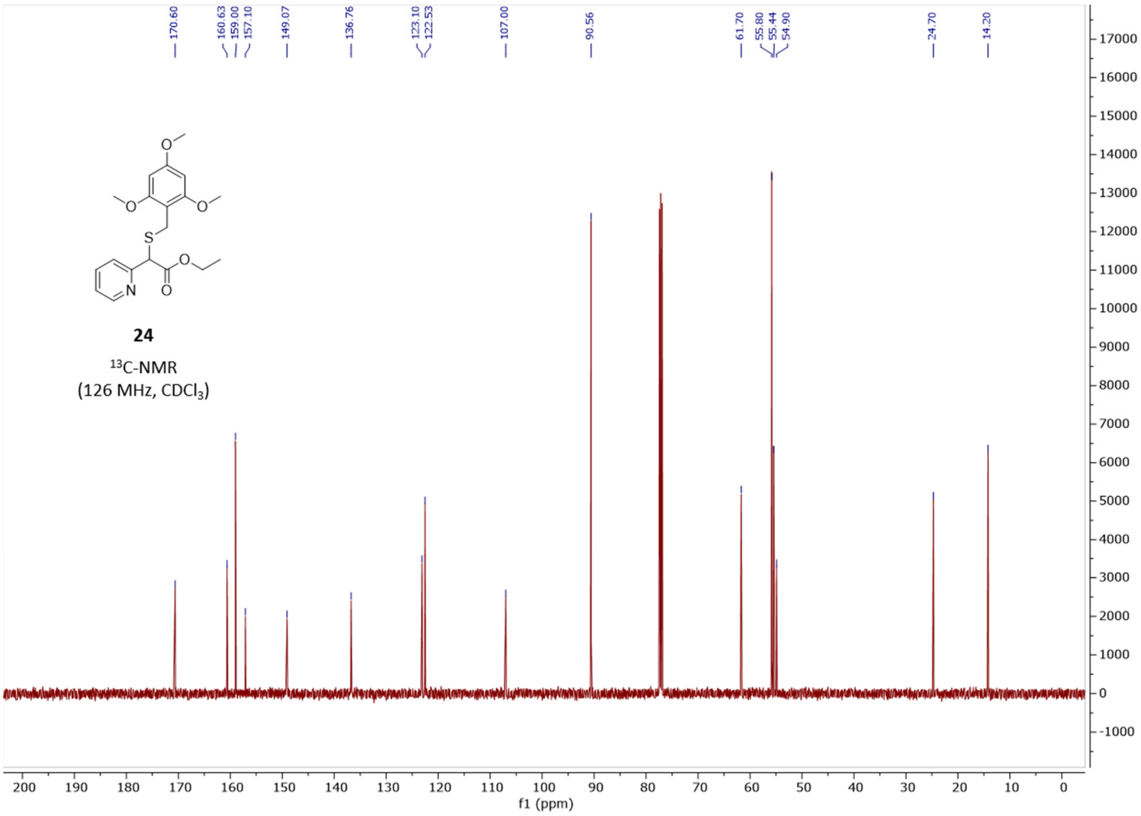

Figure S2: ^{13}C NMR spectrum (126 MHz) of ester **24** in $CDCl_3$.

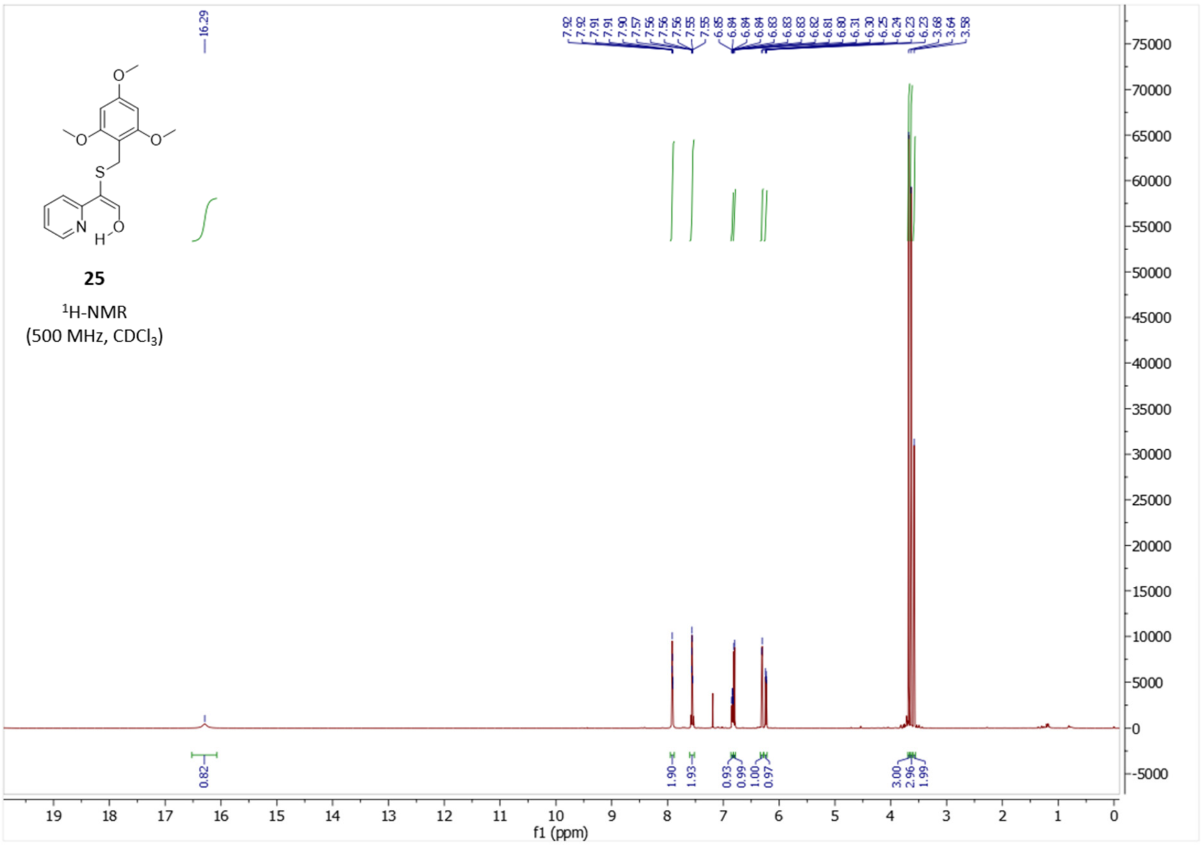

Figure S3: ^{1}H NMR spectrum (500 MHz) of enol **25** in $CDCl_3$ (0 - 20 ppm).

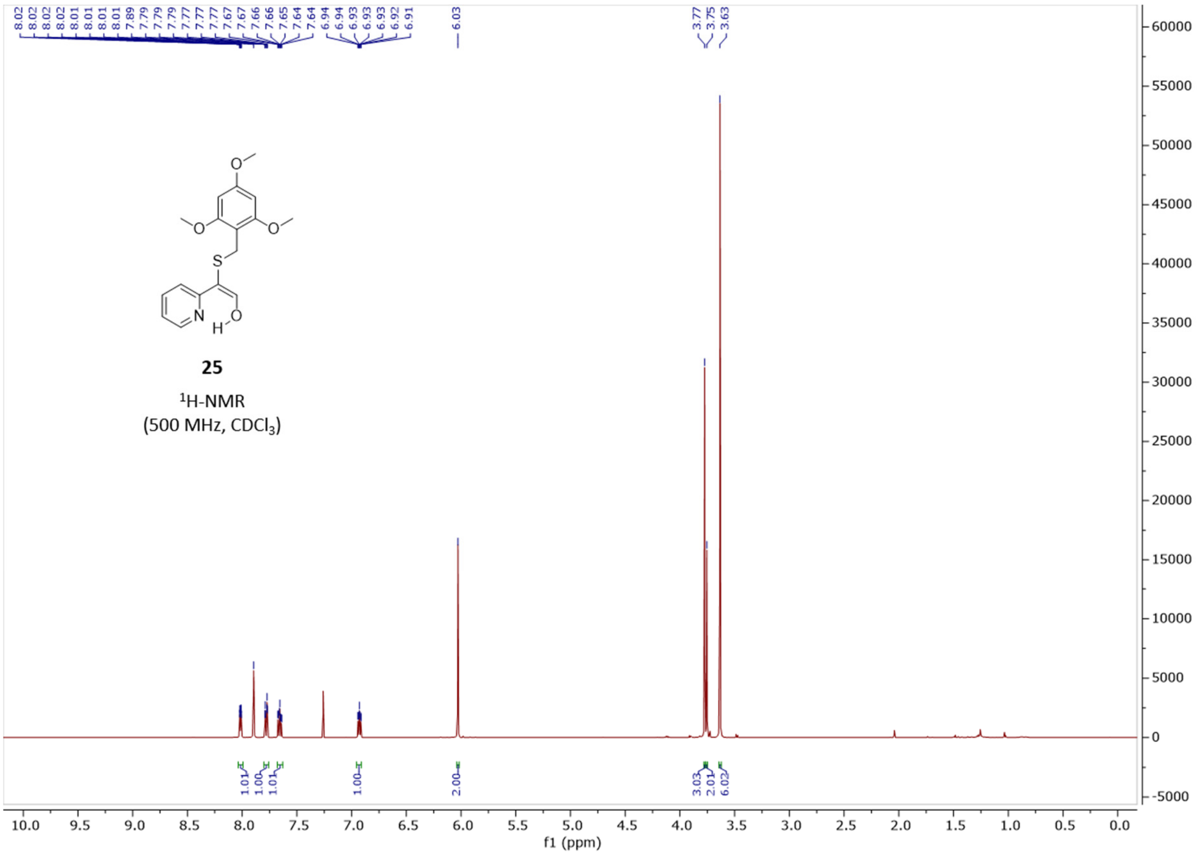

Figure S4: ^{1}H NMR spectrum (500 MHz) of enol **25** in $CDCl_3$ (0 - 10 ppm).

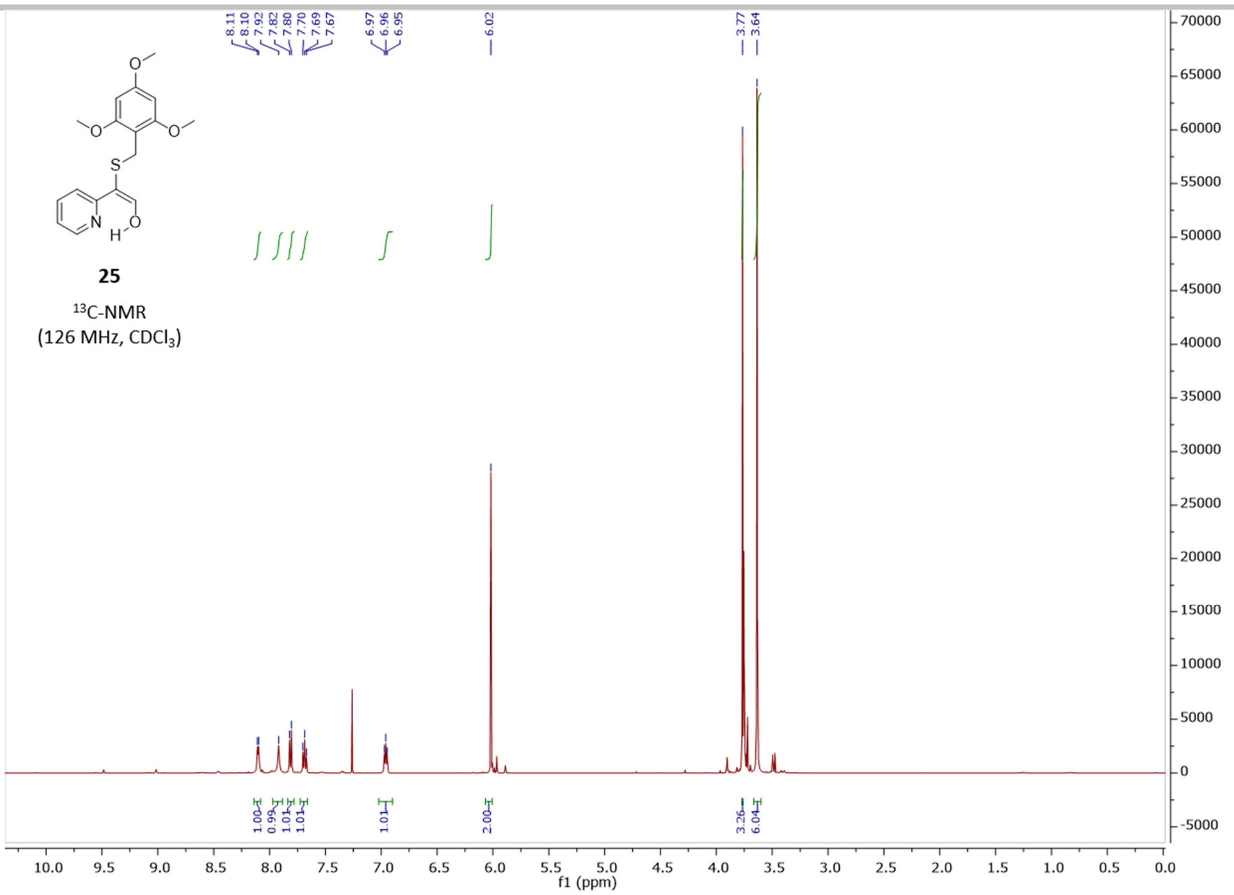

Figure S5: ^{1}H NMR spectrum (500 MHz) of enol **25** in $CDCl_3$ after 7-month storage time.

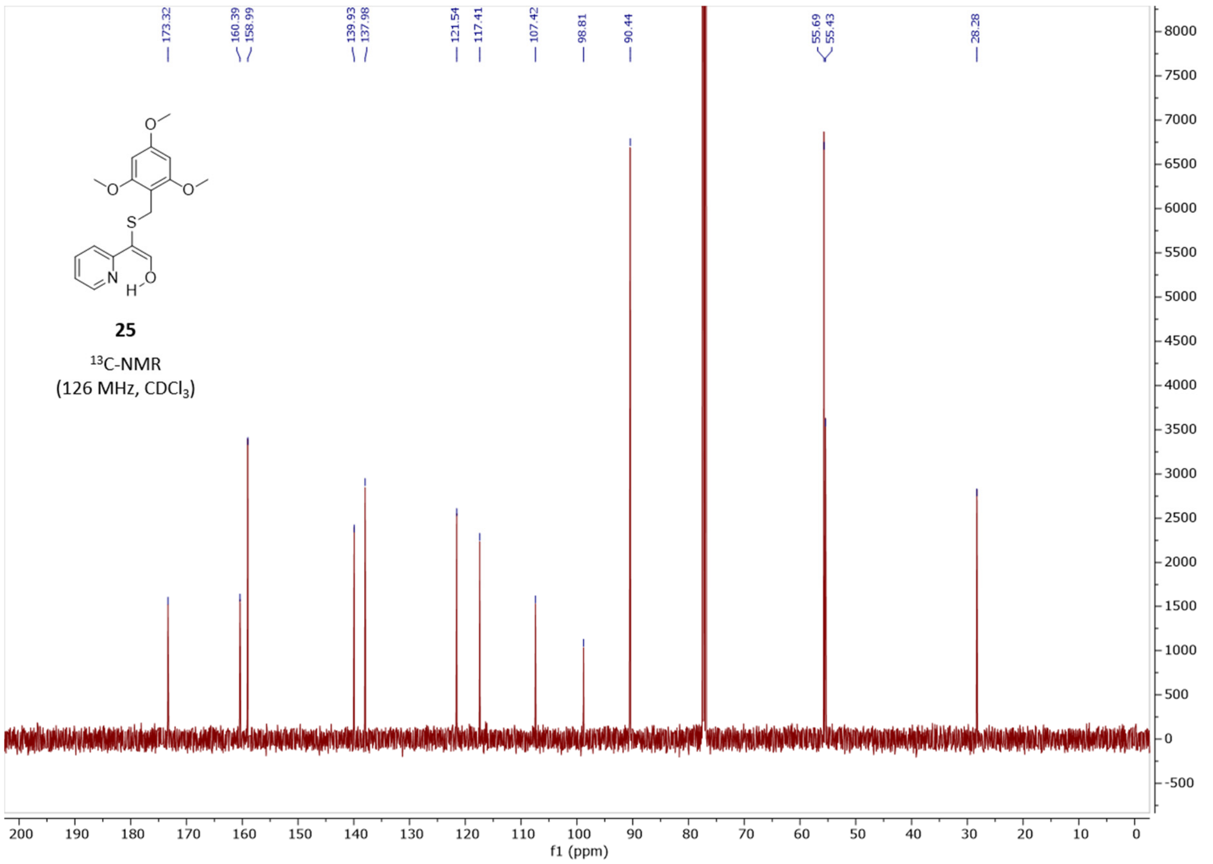

Figure S6: ^{13}C NMR spectrum (126 MHz) of ester **25** in $CDCl_3$.

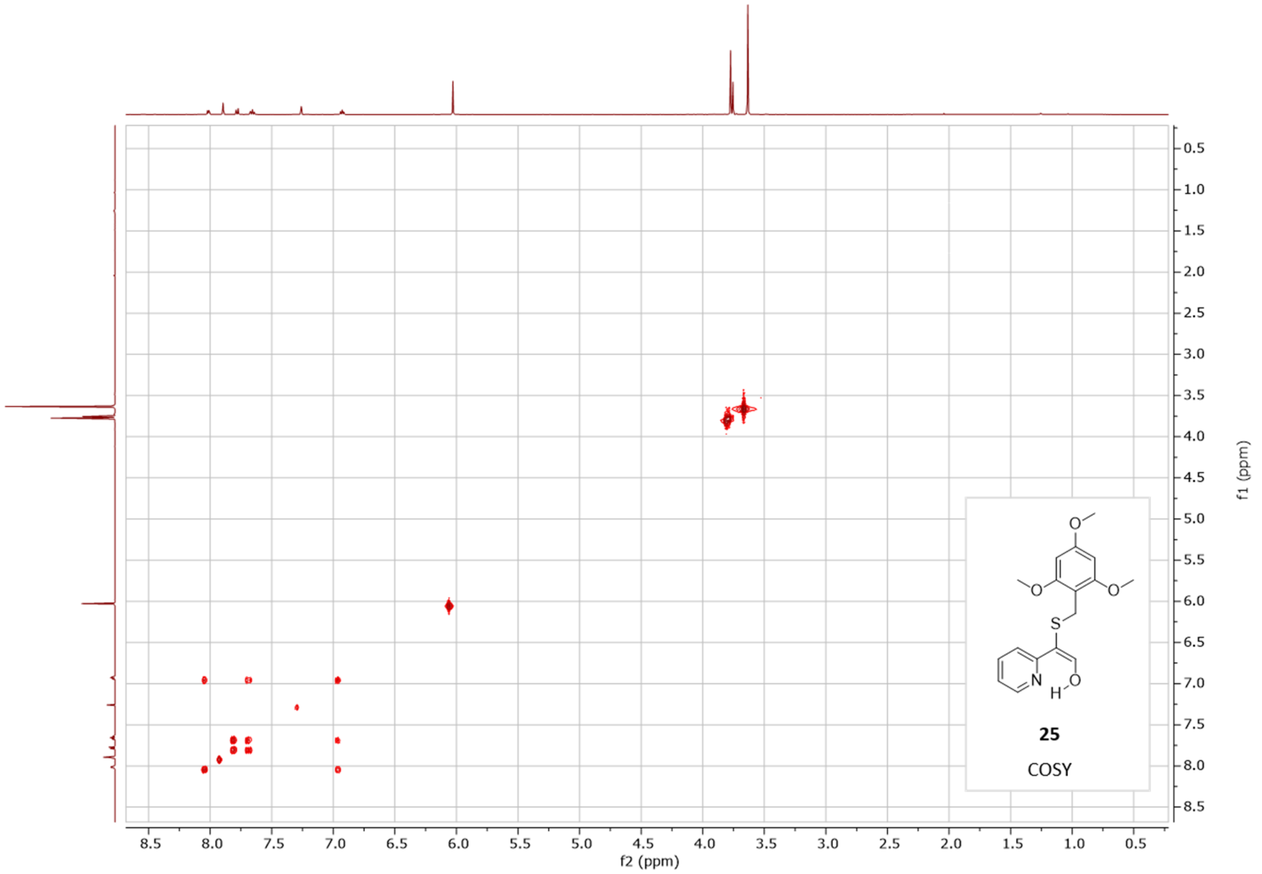

Figure S7: COSY NMR spectrum of enol **25** in $CDCl_3$.

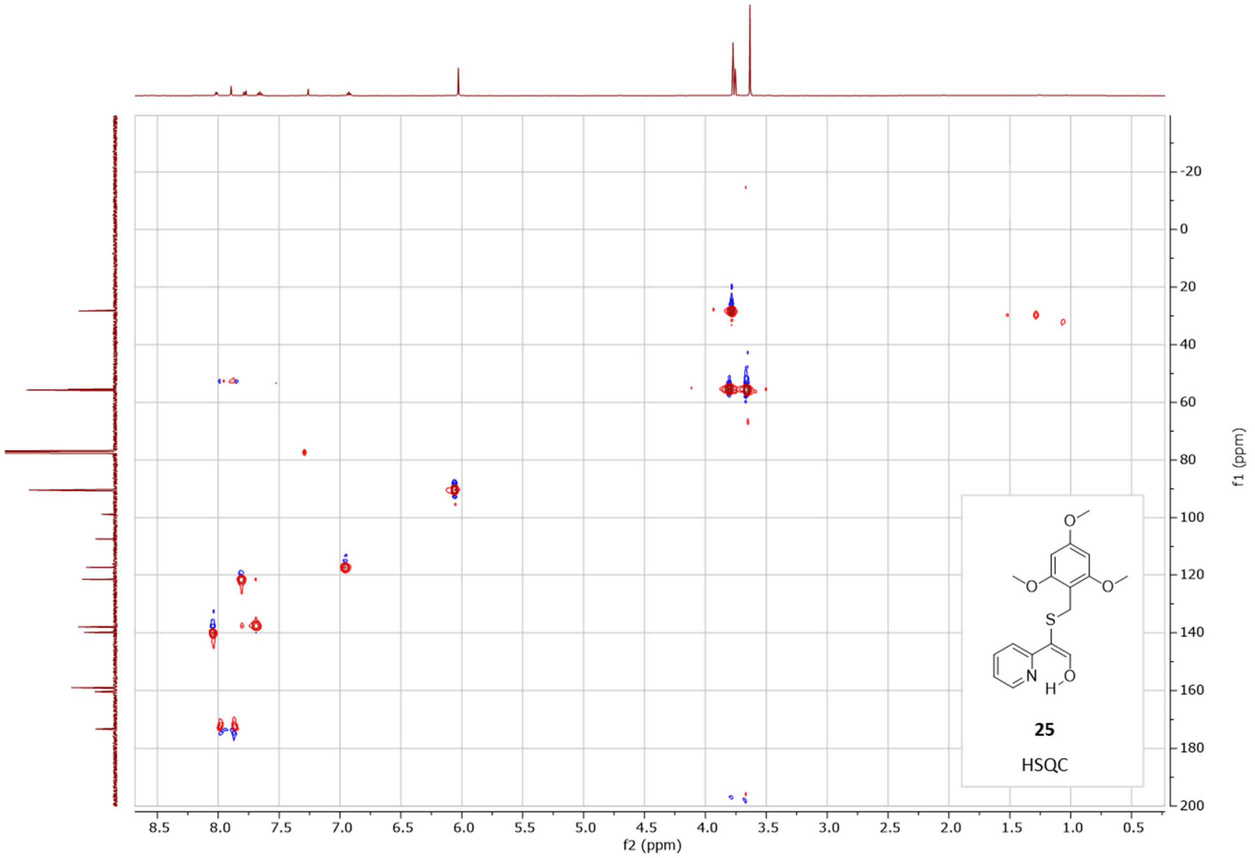

Figure S8: HSQC of enol **25** in $CDCl_3$.

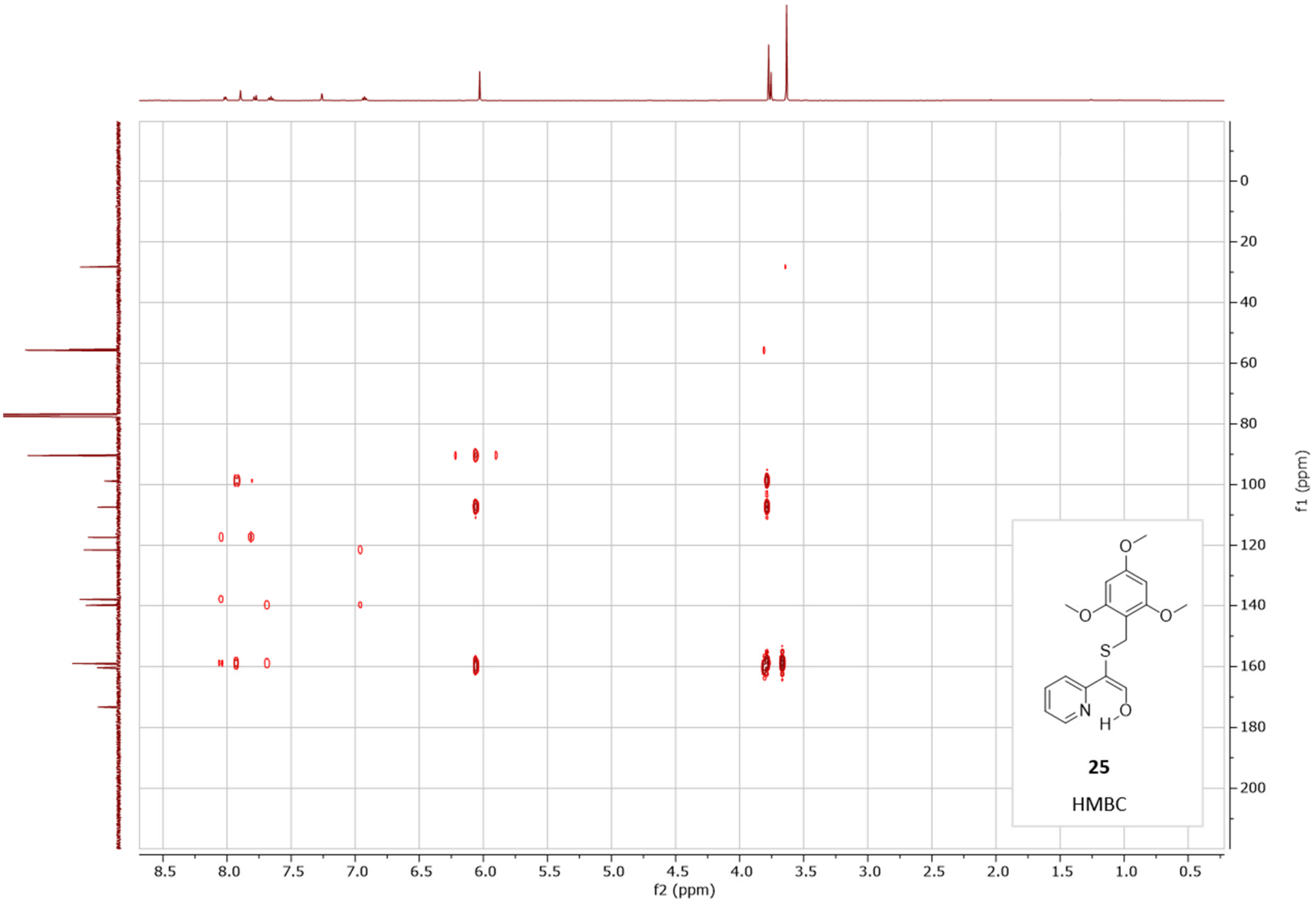

Figure S9: HMBC of enol **25** in $CDCl_3$.

4 Synthesis of peptides

4.1 Fmoc-strategy

Loading of Tentagel Rink Amide Resin:

The resin (~0.18 µmol/mg) was transferred into a syringe equipped with a filter frit and swollen in DMF (10 min). The Fmoc-group was removed by treatment of the resin with a solution of 20 % piperidine in DMF (2x 5 min). The resin was washed (3x DMF, 3x DCM, 3x DMF) and the Fmoc-protected amino acid (4 eq., c = 0.2 M in DMF) coupled in presence of PyBOP (4 eq.) and DIPEA (12 eq.). After 1 h the resin was washed (3x DMF, 3x DCM, 3x DMF), acetylated with DMF:Ac_2O:Lutidine (89:5:6 v/v/v, 5 min) and then washed (3x DMF, 3x DCM, 3x DMF). The Fmoc-group was removed by treatment of the resin with a solution of 20 % piperidine in DMF (2x 5 min) and the initial loading estimated by quantification of the piperidine-fulvene adduct (λ = 301 nm, ε = 7800 M^{-1} cm^{-1}). Afterwards the resin was washed (3x DMF, 3x DCM, 3x DMF).

Loading of Polystyrol 2-Chlorotrityl Resin: The resin (~1.02 µmol/mg) was transferred into a syringe equipped with a filter frit, allowed to swell in DCM (10 min) and the Fmoc-protected amino acid (4 eq, c = 0.1 M in DCM) was coupled in presence of DIPEA (20 eq.). After 1 h the resin was washed (10x DCM) capped with a solution of DIPEA (50eq.) in MeOH:DCM (1:3 v/v) and then washed again (5x DCM, 3x DMF). The Fmoc-group was removed by treatment of the resin with a solution of 20 % piperidine in DMF (2x 5 min) and the initial loading estimated by the UV-absorbance of the piperidine-fulvene adduct (λ = 301 nm, ε = 7800 M^{-1} cm^{-1}). Afterwards the resin was washed (3x DMF, 3x DCM, 3x DMF).

Automated Solid Phase Peptide Synthesis was performed by using a MultiPep RS from *Intavis*. Amino acids were dissolved in NMP or in magic mixture (DMF:NMP (2:1 v/v) with 1% Triton X100 and 2 M ethylencarbonate) for difficult sequences. Automated SPPS was executed using the following protocol:

- *Coupling:* Fmoc-protected amino acids (4 eq.) were activated with HCTU/OxymaPure (4 eq. each, c = 0.2 M) and DIPEA (10 eq.) and transferred to the resin (coupling time = 30 min).
- *Capping:* The resin was treated with DMF:Ac_2O:lutidine (89:5:6 v/v/v) for 5 min.
- *Deprotection:* The resin was treated with 20 % piperidine in DMF for 1x 8 min and 1x 6 min.
- *Final cleavage:* The resin was washed with DCM and dried under vacuum. Then a mixture of TFA:TIS:H_2O (95:2.5:2.5 v/v/v, 3 mL/10 µmol peptide) was added to the resin. After 2 h the cleavage cocktail was collected by filtration, the resin was washed with TFA (3x 0.5 mL) and the combined filtrates were concentrated (~ 1 mL) under argon flow.
- *Peptide precipitation:* To the remaining residue Et_2O (~ 8-10-fold volume) was added, the suspension was cooled (in dry ice ~30 min) and centrifuged (4000 rpm, 15 min, 4 °C). Afterwards the ether phase was decanted. The remaining peptide pellet was dissolved in H_2O:ACN:TFA (1:1:0.001 v/v/v) and purified by preparative HPLC as indicated.

Thioester Formation after SPPS on Trityl Resin:

After final Fmoc-deprotection the resin was treated with a solution of Boc-anhydride (50 eq., c = 0.4 M) and DIPEA (10 eq.) in DMF for 1 h to protect the N-terminal amino group. The peptides were cleaved from the trityl resin by treatment with 30 vol.% HFIP in DCM (2 mL/10 µmol peptide) for 2 h and the cleavage solution was collected by filtration and transferred into a round bottom flask. The resin was washed with DCM (2x 1 mL) and the combined filtrates were concentrated in vacuo. To the resulting residue, a mixture of *N*,*N'*-diisopropylcarbodiimide (30 eq., c = 0.15 M), OxymaPure (30 eq.) and DIPEA (40 eq.) in DMF was added. Thiophenol (30 eq.) was added and the mixture was heated to 55 °C under an argon atmosphere. After 30 min the solvent was removed via coevaporation with heptane and the residue dried at high vacuum overnight. The protecting groups were removed via treatment with TFA:TIS:H_2O (95:2.5:2.5 v/v/v; 3 mL/10 µmol peptide). After 1 h the cleavage cocktail was reduced under a stream of Argon. Et_2O (~8-10-fold volume) was added to the remaining solution which was subsequently cooled (in dry ice ~30 min), centrifuged (4000 rpm, 15 min, 4 °C) and the ether decanted. Afterwards the precipitate was suspended a second time in ether, centrifuged, decanted, and the pellet dissolved in H_2O:ACN:TFA (1:1:0.001 v/v/v) and purified by preparative HPLC as indicated.

Selenoester Formation after SPPS on 2-Chlorotrityl Resin:

After final Fmoc-deprotection the resin was treated with a solution of Boc-anhydride (50 eq.) and DIPEA (10 eq.) in DMF (0.4 M) for 1 h to protect the N-terminal amino group. The peptides were cleaved from the 2-chlorotrityl resin by treatment with 30 vol.% HFIP in DCM (2 mL/10 µmol peptide) for 2 h and the cleavage solution was collected by filtration and transferred into a round bottom flask. The resin was washed with DCM (2x 1 mL) and the combined filtrates were concentrated in vacuo. The resulting residue was dissolved in anhydrous DMF (300 µl/10 µmol peptide) and cooled to 0 °C. Diphenyldiselenide (30 eq., c = 1 M) in anhydrous DMF was added to the solution followed by $n$$Bu_3P$ (30 eq.). The reaction was allowed to warm to room temperature and proceed for 3 h, after which time the solvent was removed in vacuo. The crude material was cooled to 0 °C and the protecting groups removed via treatment with TFA:TIS:H_2O (95:2.5:2.5 v/v/v; 3 mL/10 µmol peptide). After 1 h the cleavage cocktail was reduced under a stream of Argon. Et_2O (~8-10-fold volume) was added to the remaining solution which was subsequently cooled (in dry ice ~30 min) and centrifuged (4000 rpm, 15 min, 4 °C) and the ether decanted. Afterwards the precipitate was suspended a second time in ether, centrifuged, decanted, and the pellet dissolved in H_2O:ACN:TFA (1:1:0.001 v/v/v) and purified by preparative HPLC as indicated.[2]

4.2 Boc-strategy for the Synthesis of Peptide Thioester

Loading of MBHA Resin with MPA-Gly

The resin (~0.67 µmol/mg) was transferred into a syringe equipped with a filter frit and allowed to swell in DCM (10 min). The resin was treated with a solution of DCM:DIPEA (9:1 v/v) and subsequently washed (10x DCM, 5x DMF). Fmoc-Gly-OH (4 eq., c = 0.2 M in DMF) was coupled in presence of PyBOP (4 eq.) and DIPEA (12 eq.). After 1 h the resin was washed (3x DMF, 3x DCM, 3x DMF), acetylated with DMF:Ac_2O:lutidine (89:5:6 v/v/v, 5 min) and washed (3x DMF, 3x DCM, 3x DMF). The Fmoc-group was removed by treatment of the resin with a solution of 20 % piperidine in DMF (2x 5 min) and the initial loading estimated by the UV-absorbance of the piperidine fulvene adduct (λ = 301 nm, ε = 7800 $M^{-1}cm^{-1}$). Afterwards the resin was washed (3x DMF, 3x DCM, 3x DMF). Then *S*-trityl-3-mercaptopropionic acid (4 eq., c = 0.2 M in DMF) was coupled in presence of PyBOP (4 eq.) and

DIPEA (12 eq.). After 1 h the resin was washed (3x DMF, 3x DCM, 3x DMF), acetylated with DMF:Ac_2O:lutidine (89:5:6 v/v/v, 5 min) and washed (5 x DMF, 10 x DCM). The trityl group was removed by treatment of the resin with a solution of TFA:TIS (95:5 v/v; 2 x 5 min). Afterwards the resin was washed (10x DCM, 5x DMF) and the first Boc-protected amino acid (4 eq., c = 0.2 M in DMF) was coupled in presence of PyBOP (4 eq.) and DIPEA (12 eq.) for one hour. The subsequent synthesis was done, as described below.

Manual Solid Phase Peptide Synthesis (Boc-strategy) was performed by using the following protocol:

- *Coupling*: Boc-protected amino acids (4 eq.) were activated with HCTU/OxymaPure (4 eq. each), DIPEA (12 eq.) in DMF (final concentration = 0.2 M) and transferred to the resin (coupling time = 20 min).
- *Capping*: The resin was treated with DMF:Ac_2O:lutidine (89:5:6 v/v/v, 5 min) for 3 min.
- *Deprotection*: The resin was treated 5 minutes with TFA:*m*Cresol (95:5 v/v).
- *Final cleavage*: The resin was washed with DCM and dried under vacuum. Then a mixture of TFA:TFMSA:*m*Cresol (16:3:1 v/v/v; 3 mL/10 µmol peptide) was added to the resin. After 2 h the cleavage cocktail was collected by filtration, the resin washed with TFA (3x 0.5 mL) and the combined filtrates were concentrated (~ 1 mL).
- *Peptide precipitation*: To the remaining residue Et_2O (~8-10-fold volume) was added, the suspension was cooled (in dry ice ~30 min) and centrifuged (4000 rpm, 15 min, 4 °C). Afterwards the ether phase was decanted, the remaining peptide was dissolved in H_2O/ACN/TFA (1:1:0.001 v/v/v) and purified by preparative HPLC as indicated.

4.3 Determination of Peptide and Protein Concentration

Spectroscopic determination: Concentrations of peptides containing a Tyr (ε_{280} = 1490 M^{-1} cm^{-1})- or Trp (ε_{280} = 5690 M^{-1} cm^{-1})-residue were determined by dissolving the peptides in a defined volume of H_2O/ACN/TFA (1:1:0.001 v/v/v) and measuring the absorbance (λ = 280 nm) on a *NanoDrop ND-1000* spectrometer considering the molar extinction coefficient of the peptide. In cases of auxiliary-modified peptides, the absorbance of the 2-mercaptopyridine moiety was determined as ε_{280} = 1750 M^{-1} cm^{-1}.

Gravimetric determination: Concentrations of peptides bearing no Tyr- or Trp-residue were determined by weighing of the lyophilized peptide and dissolving it afterwards in a certain volume. Basic amino acid residues (Arg, Lys, His) and free N-terminus were assumed to be present in their corresponding TFA salts and considered for the MW of the weighted peptide

.

5 Synthesis of Peptide Chalkoester

5.1 Peptide Thioester

LYRAA-S(CH_2)$_2$(CO)-Gly **(28A)**

The synthesis was carried out on a MBHA resin (~0.67 µmol/mg) in a 25 µM scale following the manual Boc-strategy protocol for the synthesis of peptide thioester (see 4.2). After cleavage from the resin and peptide precipitation, the crude peptide thioester was purified by preparative HPLC using a linear gradient from 3-30 % B in 40 min. The desired peptide thioester **28A** was isolated as a white solid after lyophilization (see figure S10), which was dissolved in H_2O/ACN/TFA (1:1:0.001 v/v/v) for a spectroscopic determination of the synthesis yield (A_{280} = 0.51, V = 2.0 mL, 6.9 µmol, 28 %).

UPLC-MS: t_R = 1.71 min (3-30 % B in 4 min); m/z = 737.5 ($C_{32}H_{52}N_{10}O_8S$ $(M+H)^+$, calcd.: 737.4), 369.2. ($C_{32}H_{52}N_{10}O_8S$ $(M+2H)^{2+}$, calcd.: 369.2); $C_{32}H_{52}N_{10}O_8S$ (MW = 736.4 g· mol^{-1}).

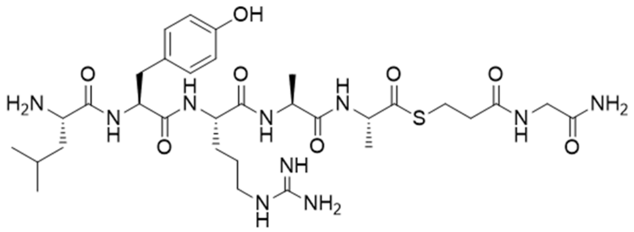

28A

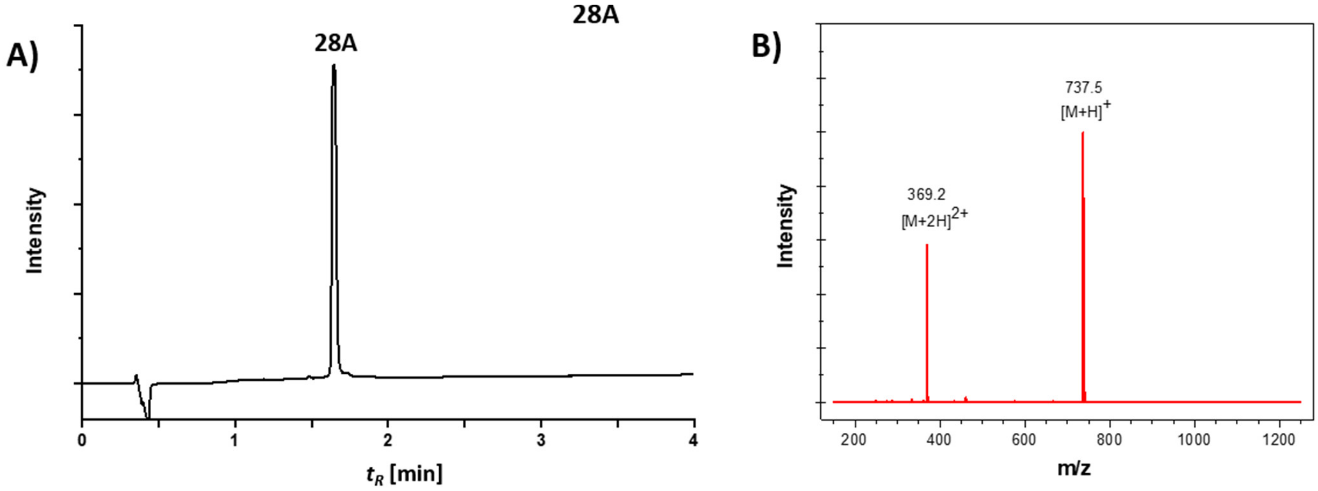

Figure S10: UPLC-trace A) and ESI-MS spectrum B) of purified peptide thioester **28A**. UPLC analysis: 3 - 30 % in 4 min, λ = 210 nm.

LYRAL-S(CH_2)$_2$(CO)-Gly **(28L)**

The synthesis was carried out on a MBHA resin (~0.67 µmol/mg) in a 25 µM scale following the manual Boc-strategy protocol for the synthesis of peptide thioester (see 4.2). After cleavage from the resin and peptide precipitation, the crude peptide thioester was purified by preparative HPLC using a linear gradient from 3-30 % B in 40 min. The desired peptide thioester **28L** was isolated as a white solid after lyophilization (see figure S11), which was dissolved in H_2O/ACN/TFA (1:1:0.001 v/v/v) for a spectroscopic determination of the synthesis yield (A_{280} = 1.14, V = 2.0 mL, 15.3 µmol, 61 %).

UPLC-MS: t_R = 2.79 min (3-30 % B in 4 min); m/z = 779.5 ($C_{35}H5_8N_{10}O_8S$ $(M+H)^+$, calcd.: 779.4), 390.3. ($C_{35}H5_8N_{10}O_8S$ $(M+2H)^{2+}$, calcd.: 390.2); $C_{35}H5_8N_{10}O_8S$ (MW = 778.4 g· mol^{-1}).

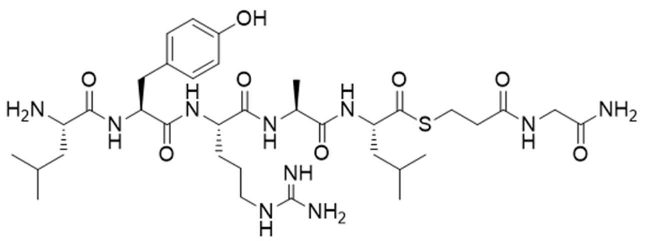

28L

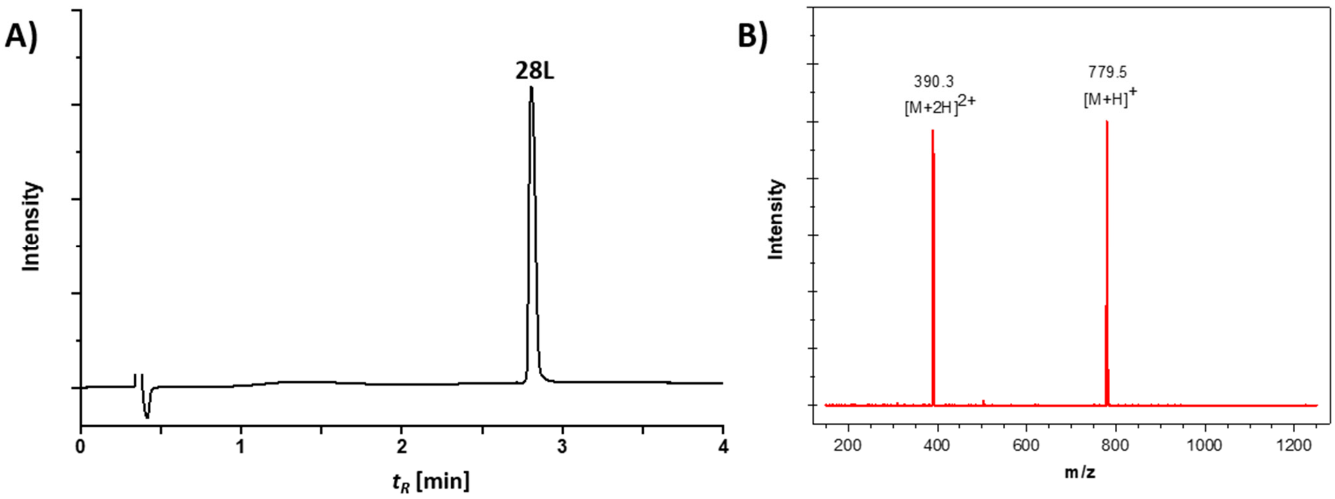

Figure S11: UPLC-trace A) and ESI-MS spectrum B) of purified peptide thioester **28L**. UPLC analysis: 3 - 30 % in 4 min, λ = 210 nm.

LYRAP-S(CH_2)$_2$(CO)-Gly **(28P)**

The synthesis was carried out on a MBHA resin (~0.67 µmol/mg) in a 10 µM scale following the manual Boc-strategy protocol for the synthesis of peptide thioester (see 4.2). After cleavage from the resin and peptide precipitation, the crude peptide thioester was purified by preparative HPLC using a linear gradient from 3-30 % B in 40 min. The desired peptide thioester **28P** was isolated as a white solid after lyophilization (see figure S12), which was dissolved in H_2O/ACN/TFA (1:1:0.001 v/v/v) for a spectroscopic determination of the synthesis yield. (A_{280} = 0.42, V = 1.0 mL, 2.8 µmol, 28 %)

UPLC-MS: t_R = 2.68 min (3-30 % B in 6 min); m/z = 763.6 ($C_{34}H_{54}N_{10}O_8S$ $(M+H)^+$, calcd.: 763.4), 390.3 ($C_{34}H_{54}N_{10}O_8S$ $(M+2H)^{2+}$, 382.4 calcd.: 382.2); $C_{34}H_{54}N_{10}O_8S$ (MW = 762.4 g· mol^{-1}).

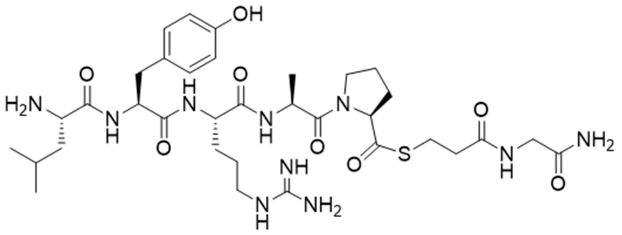

28P

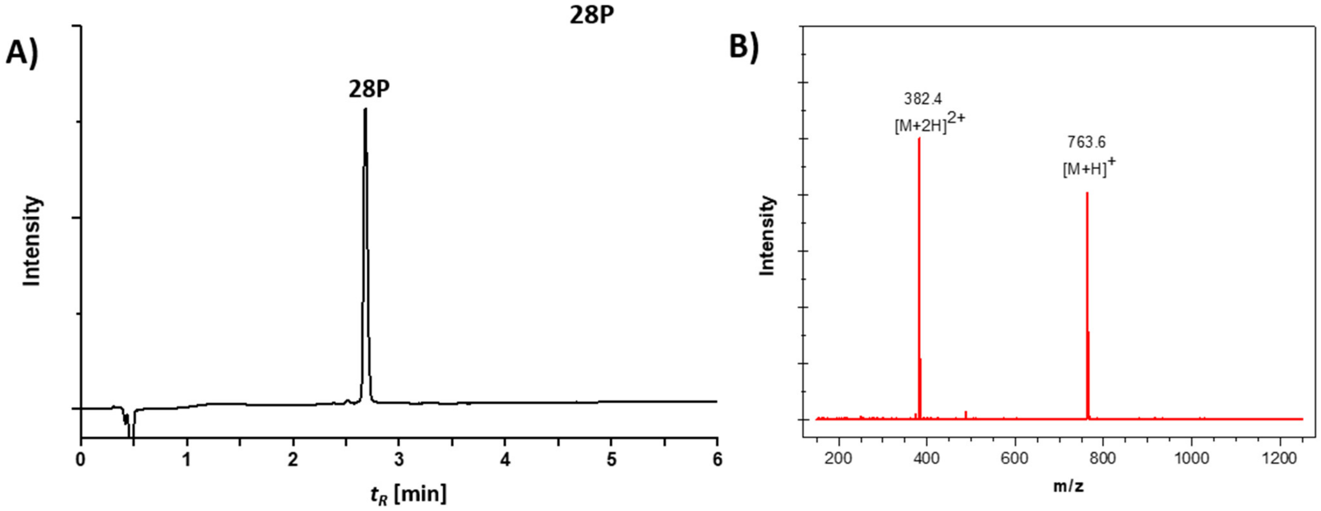

Figure S12: UPLC-trace A) and ESI-MS spectrum B) of purified peptide thioester **28P**. UPLC analysis: 3 - 30 % in 6 min, λ = 210 nm.

5.2 Peptide Selenoester

LYRAA-SePh **(31A)**

The synthesis was carried out on a polystyrol 2-chlorotrityl resin (~1.02 µmol/mg) in a 22 µM scale. After the initial loading step the synthesis followed the automated Fmoc-strategy protocol with subsequent selenoester formation (see 4.1). After removal of all protecting groups and peptide precipitation, the crude peptide selenoester was purified by preparative HPLC using a linear gradient from 3-30 % B in 40 min. The desired peptide selenoester **31A** was isolated as a white solid after lyophilization (see figure S13), which was dissolved in H_2O/ACN/TFA (1:1:0.001 v/v/v) for a spectroscopic determination of the synthesis yield (A_{280} = 0.83, V = 2.0 mL, 11.1 µmol, 50 %).

UPLC-MS: t_R = 2.68 min (3-50 % B in 4 min); m/z = 733.5 ($C_{33}H_{48}N_8O_6Se$ $(M+H)^+$, calcd.: 733.3), 367.5 ($C_{33}H_{48}N_8O_6Se$ (M+2H)2+, calcd.: 367.1); $C_{33}H_{48}N_8O_6Se$ (MW = 732.3 g· mol^{-1}).

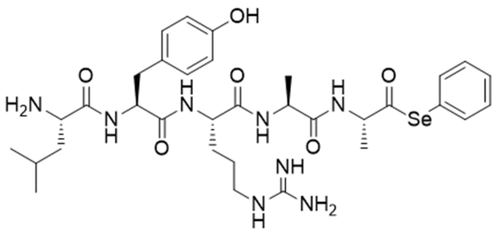

31A

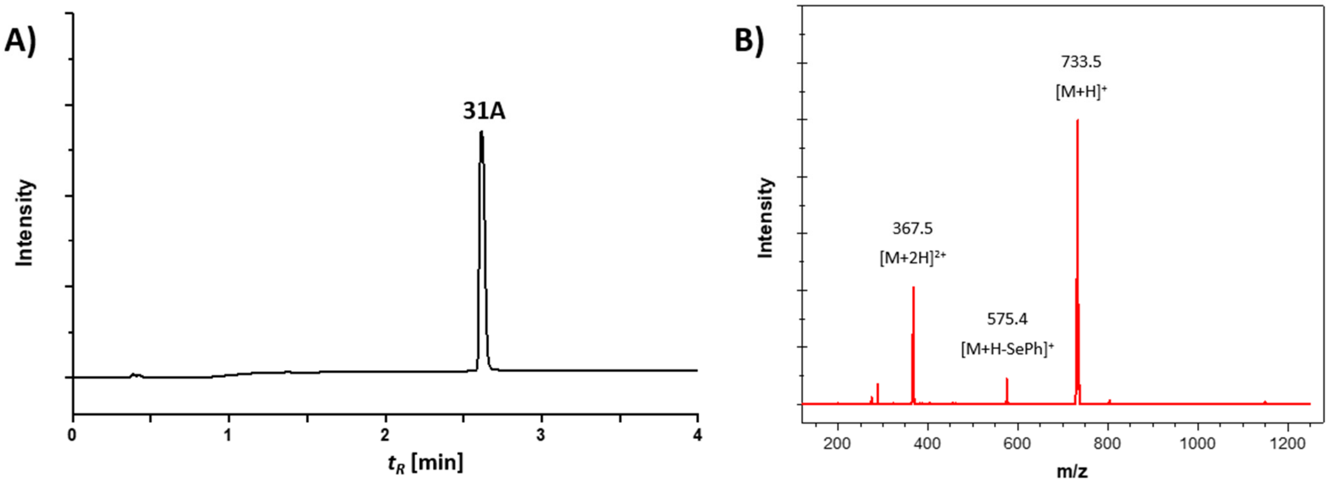

Figure S13: UPLC-trace A) and ESI-MS spectrum B) of purified peptide selenoester **31A**. UPLC analysis: 3 - 50 % in 4 min, λ = 210 nm.

LYRAL-SePh **(31L)**

The synthesis was carried out on a polystyrol 2-chlorotrityl resin (~1.02 µmol/mg) in a 20 µM scale. After the initial loading step the synthesis followed the automated Fmoc-strategy protocol with subsequent selenoester formation (see 4.1). After removal of all protecting groups and peptide precipitation, the crude peptide selenoester was purified by preparative HPLC using a linear gradient from 3-30 % B in 40 min. The desired peptide selenoester **31L** was isolated as a white solid after lyophilization (see figure S14), which was dissolved in H_2O/ACN/TFA (1:1:0.001 v/v/v) for a spectroscopic determination of the synthesis yield (A_{280} = 0.48, V = 2.0 mL, 6.4 µmol, 32 %).

UPLC-MS: t_R = 5.39 min (3-35 % B in 6 min); m/z = 775.5 ($C_{36}H_{54}N_8O_6Se$ $(M+H)^+$, calcd.: 775.3), 388.4 ($C_{36}H_{54}N_8O_6Se$ (M+2H)2^+, calcd.: 388.2); $C_{36}H_{54}N_8O_6Se$ (MW = 774.3 g· mol^{-1}).

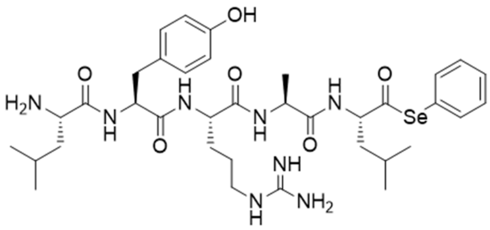

31L

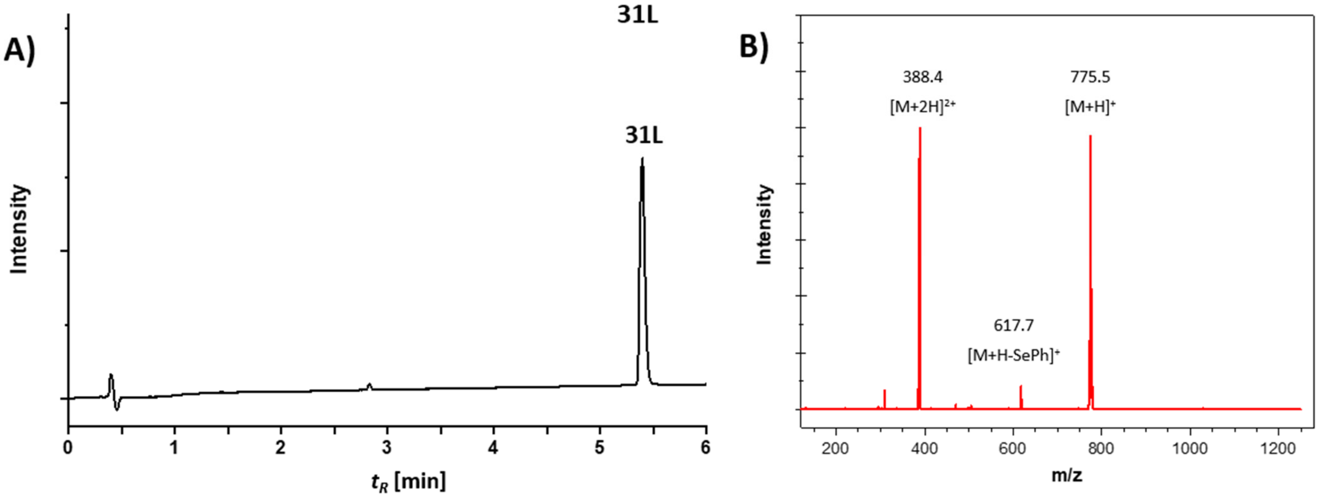

Figure S14: UPLC-trace A) and ESI-MS spectrum B) of purified peptide selenoester **31L**. UPLC analysis: 3 - 35 % in 6 min, λ = 210 nm.

LYRAP-SePh **(31P)**

The synthesis was carried out on a polystyrol 2-chlorotrityl resin (~1.02 µmol/mg) in a 15 µM scale. After the initial loading step the synthesis followed the automated Fmoc-strategy protocol with subsequent selenoester formation (see 4.1). After removal of all protecting groups and peptide precipitation, the crude peptide selenoester was purified by preparative HPLC using a linear gradient from 3-30 % B in 40 min. The desired peptide selenoester **31P** was isolated as a white solid after lyophilization (see figure S15), which was dissolved in H_2O/ACN/TFA (1:1:0.001 v/v/v) for a spectroscopic determination of the synthesis yield (A_{280} = 0.45, V = 1.0 mL, 3.0 µmol, 20 %).

UPLC-MS: t_R = 5.39 min (3-30 % B in 6 min); m/z = 759.5 ($C_{35}H_{50}N_8O_6Se$ (M+H)+, calcd.: 759.3), 380.3 ($C_{35}H_{50}N_8O_6Se$ (M+2H)2+, calcd.: 380.2); $C_{35}H_{50}N_8O_6Se$ (MW = 758.3 g· mol^{-1}).

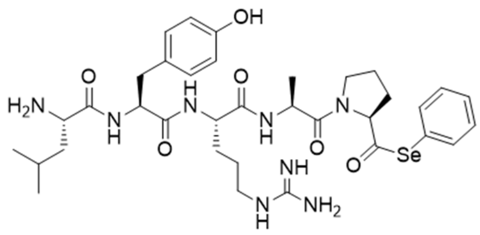

31P

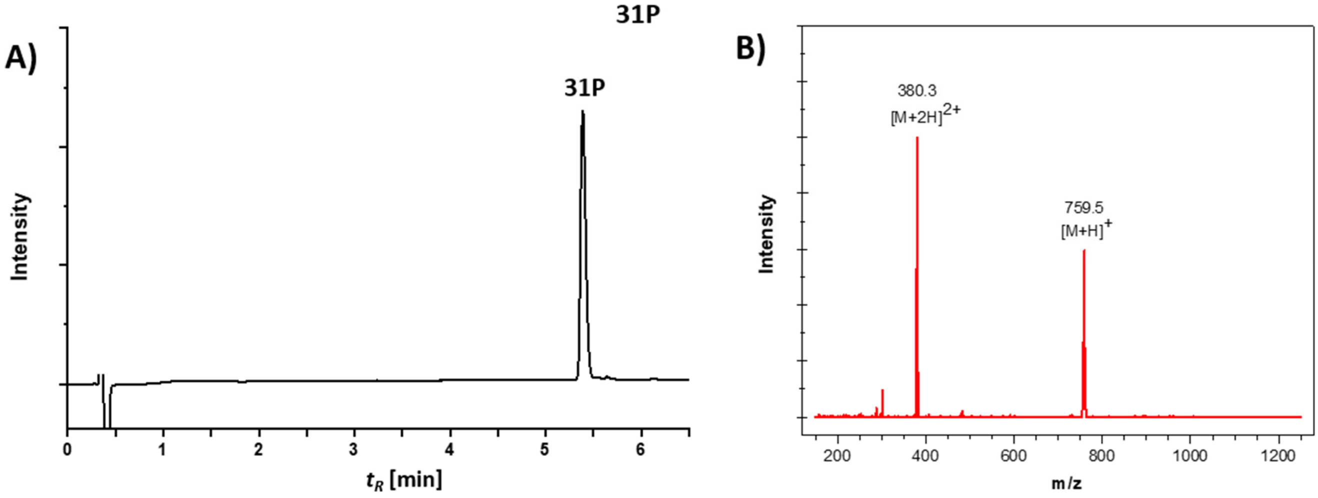

Figure S15: UPLC-trace A) and ESI-MS spectrum B) of purified peptide selenoester **31P**. UPLC analysis: 3 - 30 % in 6 min, λ = 210 nm.

6 Introduction of the Auxiliary

6.1 Introduction of the 2-Mercapto-2-(pyridin-2-yl)ethyl Auxiliary during SPPS

The peptidyl-resin was allowed to swell in MeOH:NMP:AcOH (3:1:0.02 v/v/v) for 15 min and then treated with a mixture of the enol **25** and $NaCNBH_3$ (15 eq. each, c = 0.4 M) in MeOH:NMP:AcOH (3:1:0.02 v/v/v) for 2 - 21 h. Afterwards the resin was washed with DCM (5x), MeOH (5x) and DCM (5x) and dried. Finally, the peptide was deprotected and cleaved of the resin by addition of a mixture of TFA:TIS:H_2O (95:2.5:2.5 v/v/v, 3 mL/10 µmol peptide). After 24 h the cleavage cocktail was collected by filtration, the resin was washed with TFA (3x 0.5 mL) and the filtrates were combined. Alternatively, the peptide could be cleaved off by addition of a mixture of TFA:TIS:H_2O (95:2.5:2.5 v/v/v, 3 mL/10 µmol peptide) for 2 h and subsequent treatment with TFMSA:*m*Cresol:Thioanisole:TFA (1:1:1:7 v/v/v, 3 mL/10 µmol peptide) for 2 h. The resin was washed with TFA (3x 0.5 mL) and the filtrates were combined. In both cleavage methods the filtrates were concentrated and Et_2O (~8-10-fold volume) was added. The suspension was cooled in dry ice and centrifuged (4000 rpm, 15 min, 4 °C). Afterwards the ether phase was decanted. The remaining peptide was dissolved in H_2O:ACN:TFA (1:1:0.001 v/v/v) and purified by preparative HPLC as indicated.

MPyE-**GRAEYSGLG (27G)**

Synthesis was achieved by automated solid phase peptide synthesis following the protocol described in 4.1. After removal of the last Fmoc-group (loading 20.0 µmol) the auxiliary was introduced by reductive alkylation for 2 h (see 6.1). Complete conversion was verified by UPLC-MS analysis of test cleavage reaction (see figure S16). After cleavage from the resin and peptide precipitation, the crude peptide was purified by preparative HPLC using a linear gradient from 3-30 % B in 40 min. The desired auxiliary peptide **27G** was isolated as a white solid after lyophilization, which was dissolved in H_2O/ACN/TFA (1:1:0.001 v/v/v) for a spectroscopic determination of the synthesis yield (A_{280} = 1.18, V = 2.0 mL, 7.3 µmol, 36 %).

UPLC-MS: t_R = 2.73 min (3-30 % B in 4 min); m/z = 1045.6 ($C_{45}H_{68}N_{14}O_{13}S$ $(M+H)^+$, calcd.: 1045.5), 523.3 ($C_{45}H_{68}N_{14}O_{13}S$ $(M+2H)^{2+}$, calcd.: 523.2); $C_{45}H_{68}N_{14}O_{13}S$ (MW = 1044.5 g·mol^{-1}).

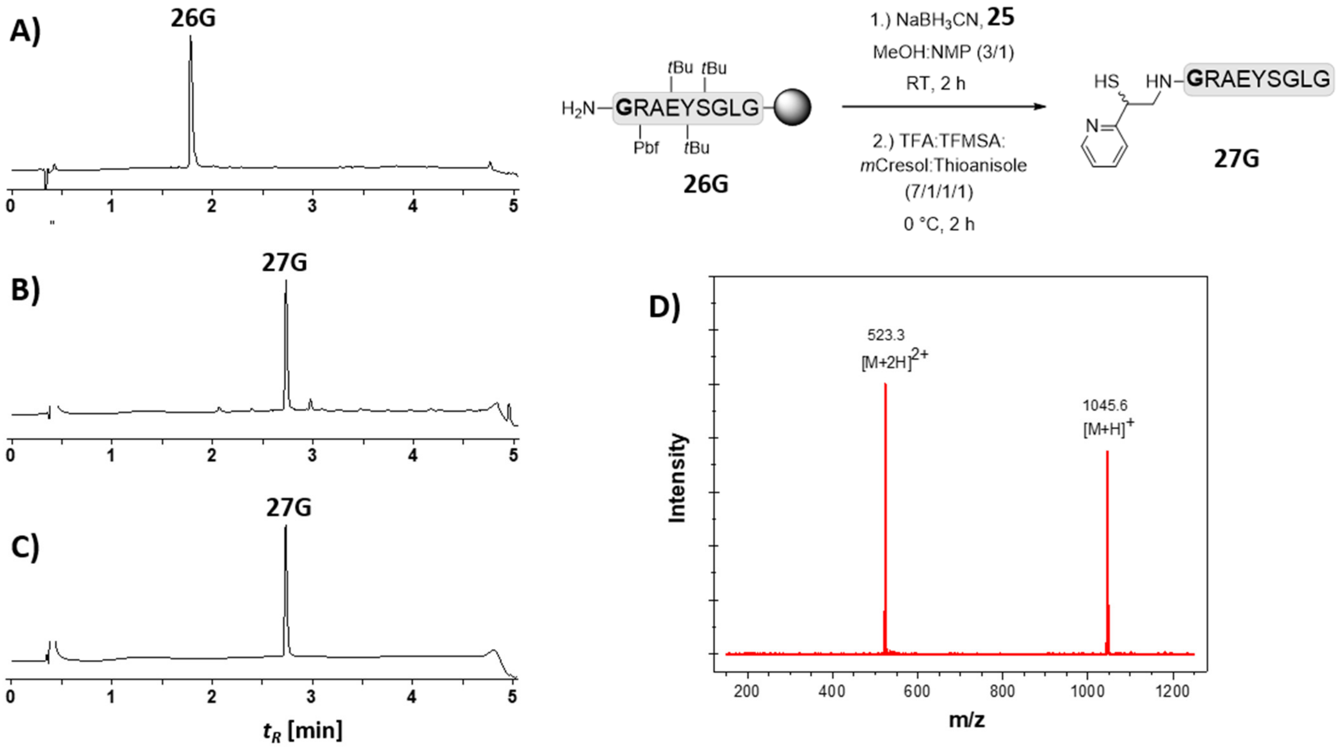

Figure S16: UPLC analysis of crude peptide obtained A) before and B) after reductive alkylation of **26G**. C) UPLC and D) MS analysis of purified product. UPLC analysis: 3 - 30 % in 4 min, λ = 210 nm.

MPyE-**NRAEYSGLG (27N)**

Synthesis was achieved by automated solid phase peptide synthesis following the protocol described in 4.1. After removal of the last Fmoc-group (loading 20.0 µmol) the auxiliary was introduced by reductive alkylation for 18 h (see 6.1). Complete conversion was verified by UPLC-MS analysis of test cleavage reaction (see figure S17). After cleavage from the resin and peptide precipitation, the crude peptide was purified by preparative HPLC using a linear gradient from 3-30 % B in 40 min. The desired auxiliary peptide **27N** was isolated as a white solid after lyophilization, which was dissolved in H_2O/ACN/TFA (1:1:0.001 v/v/v) for a spectroscopic determination of the synthesis yield (A_{280} = 1.36, V = 2.0 mL, 8.4 µmol, 42 %).

UPLC-MS: t_R = 1.97 min (3-30 % B in 4 min); m/z = 1102.5 ($C_{47}H_{71}N_{15}O_{14}S$ $(M+H)^+$, calcd.: 1102.5), 551.8 ($C_{47}H_{71}N_{15}O_{14}S$ $(M+2H)^{2+}$, calcd.: 551.7); $C_{47}H_{71}N_{15}O_{14}S$ (MW = 1101.5 g·mol^{-1}).

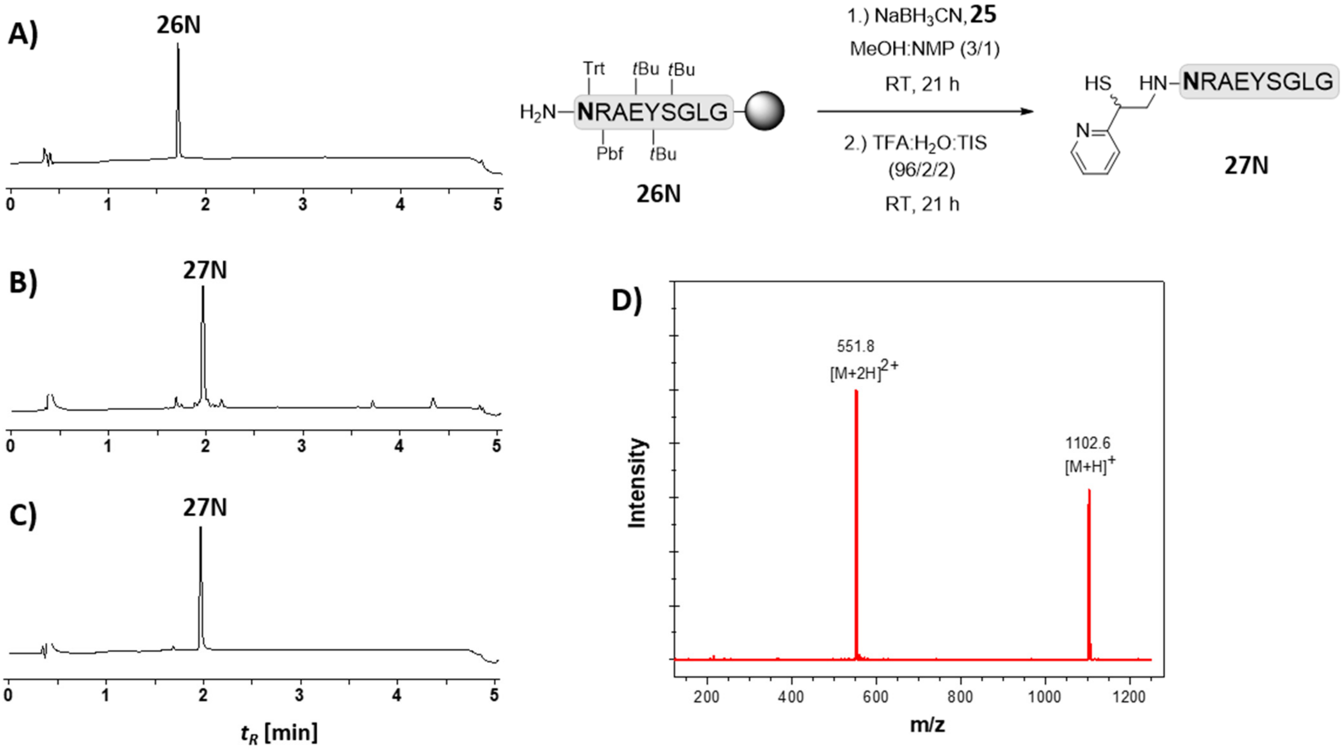

Figure S17: UPLC analysis of crude peptide obtained A) before and B) after reductive alkylation of **26N**. C) UPLC and D) MS analysis of purified product. UPLC analysis: 3 - 30 % in 4 min, λ = 210 nm.

MPyE-**RRAEYSGLG (27R)**

Synthesis was achieved by automated solid phase peptide synthesis following the protocol described in 4.1. After removal of the last Fmoc-group (loading 20.0 µmol) the auxiliary was introduced by reductive alkylation for 18 h (see 6.1). Complete conversion was verified by UPLC-MS analysis of test cleavage reaction (see figure S18). After cleavage from the resin and peptide precipitation, the crude peptide was purified by preparative HPLC using a linear gradient from 3-30 % B in 40 min. The desired auxiliary peptide **27R** was isolated as a white solid after lyophilization, which was dissolved in H_2O/ACN/TFA (1:1:0.001 v/v/v) for a spectroscopic determination of the synthesis yield (A_{280} = 1.34, V = 2.0 mL, 8.3 µmol, 41 %).

UPLC-MS: t_R = 3.02 min (3-30 % B in 6 min); m/z = 1144.7 ($C_{49}H_{77}N_{17}O_{13}S$ $(M+H)^+$, calcd.: 1144.6), 572.8 ($C_{49}H_{77}N_{17}O_{13}S$ $(M+2H)^{2+}$, calcd.: 572.8); $C_{49}H_{77}N_{17}O_{13}S$ (MW = 1143.6 g·mol^{-1}).

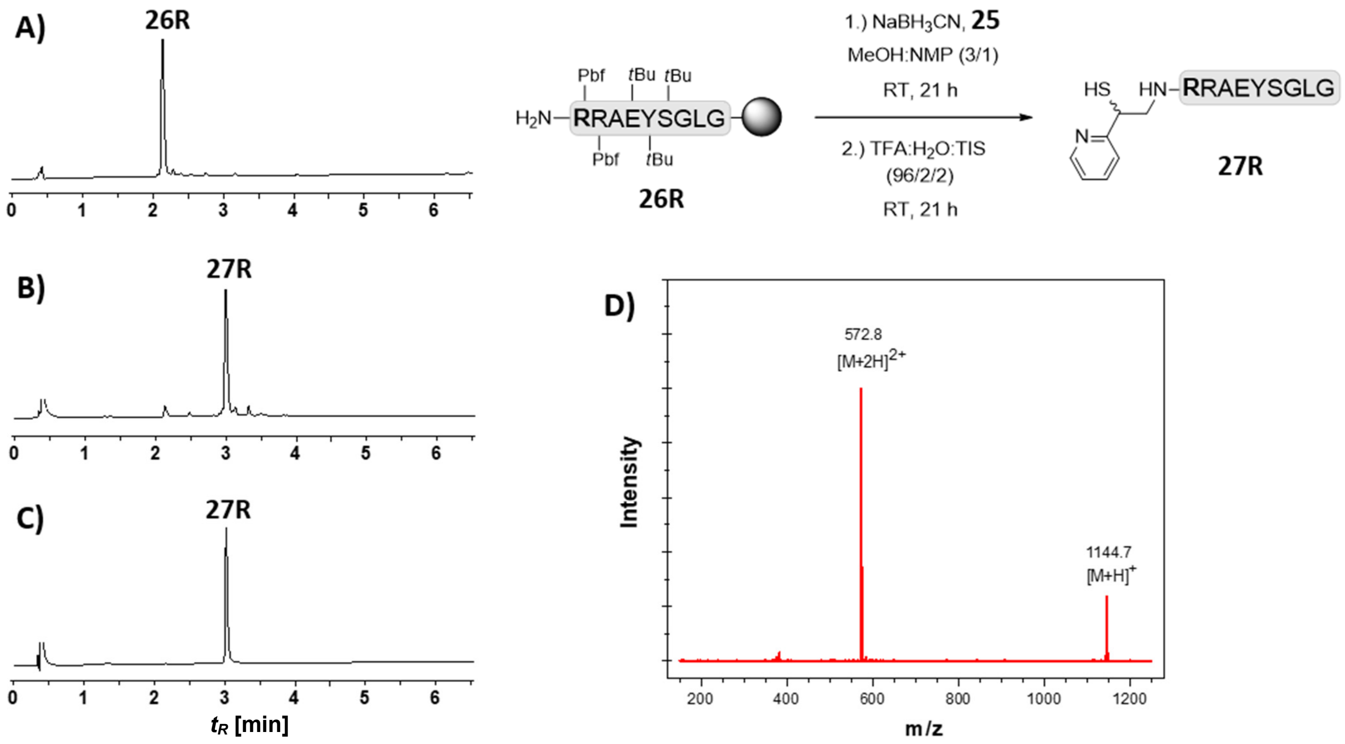

Figure S18: UPLC analysis of crude peptide obtained A) before and B) after reductive alkylation of **26R**. C) UPLC and D) MS analysis of purified product. UPLC analysis: 3 - 30 % in 6 min, λ = 210 nm.

MPyE-**VRAEYSGLG (27V)**

Synthesis was achieved by automated solid phase peptide synthesis following the protocol described in 4.1. After removal of the last Fmoc-group (loading 20.8 µmol) the auxiliary was introduced by reductive alkylation for 18 h (see 6.1). Complete conversion was verified by UPLC-MS analysis of test cleavage reaction (see figure S19). After cleavage from the resin and peptide precipitation, the crude peptide was purified by preparative HPLC using a linear gradient from 3-30 % B in 40 min. The desired auxiliary peptide **27V** was isolated as a white solid after lyophilization, which was dissolved in H_2O/ACN/TFA (1:1:0.001 v/v/v) for a spectroscopic determination of the synthesis yield (A_{280} = 1.31, V = 2.0 mL, 8.1 µmol, 39 %).

UPLC-MS: t_R = 3.66 min (3-30 % B in 6 min); m/z = 1087.7 ($C_{48}H_{74}N_{14}O_{13}S$ $(M+H)^+$, calcd.: 1087.6), 544.3 ($C_{48}H_{74}N_{14}O_{13}S$ $(M+2H)^{2+}$, calcd.: 544.3); $C_{48}H_{74}N_{14}O_{13}S$ (MW = 1086.5 g·mol^{-1}).

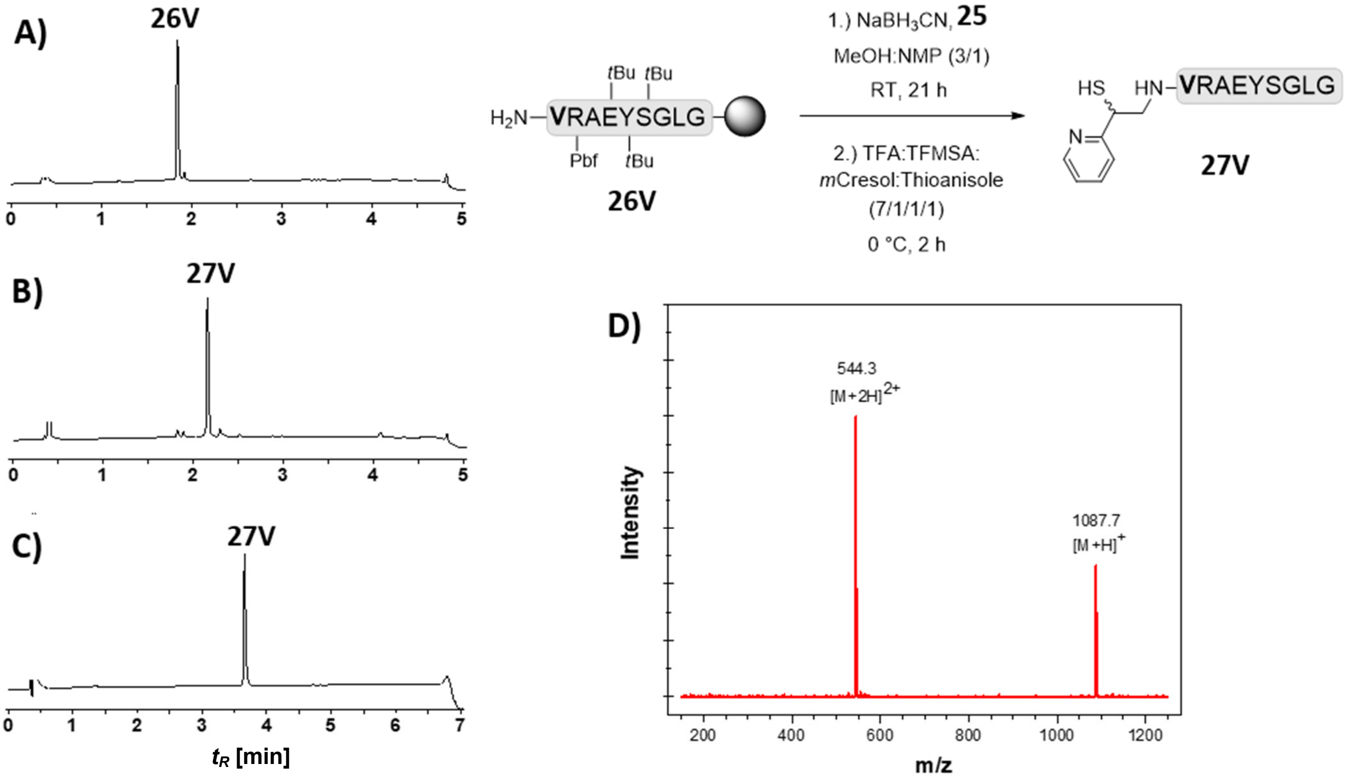

Figure S19: UPLC analysis of crude peptide obtained A) before and B) after reductive alkylation of **26V**. C) UPLC and D) MS analysis of purified product. UPLC analysis: A) and B) 3 - 30 % in 4 min, λ = 210 nm; C) 3 - 30 % in 6 min, λ = 210 nm.

MPyE-**LRAEYSGLG (27L)**

Synthesis was achieved by automated solid phase peptide synthesis following the protocol described in 4.1. After removal of the last Fmoc-group (loading 21.0 µmol) the auxiliary was introduced by reductive alkylation for 18 h (see 6.1). Complete conversion was verified by UPLC-MS analysis of test cleavage reaction (see figure S20). After cleavage from the resin and peptide precipitation, the crude peptide was purified by preparative HPLC using a linear gradient from 3-30 % B in 40 min. The desired auxiliary peptide **27L** was isolated as a white solid after lyophilization, which was dissolved in H_2O/ACN/TFA (1:1:0.001 v/v/v) for a spectroscopic determination of the synthesis yield (A_{280} = 1.31, V = 2.0 mL, 8.1 µmol, 38 %).

UPLC-MS: t_R = 2.31 min (3-30 % B in 4 min); m/z = 1101.6 ($C_{49}H_{76}N_{14}O_{13}S$ $(M+H)^+$, calcd.: 1101.5), 551.3 ($C_{49}H_{76}N_{14}O_{13}S$ $(M+2H)^{2+}$, calcd.: 551.3); $C_{49}H_{76}N_{14}O_{13}S$ (MW = 1100.5 g·mol^{-1}).

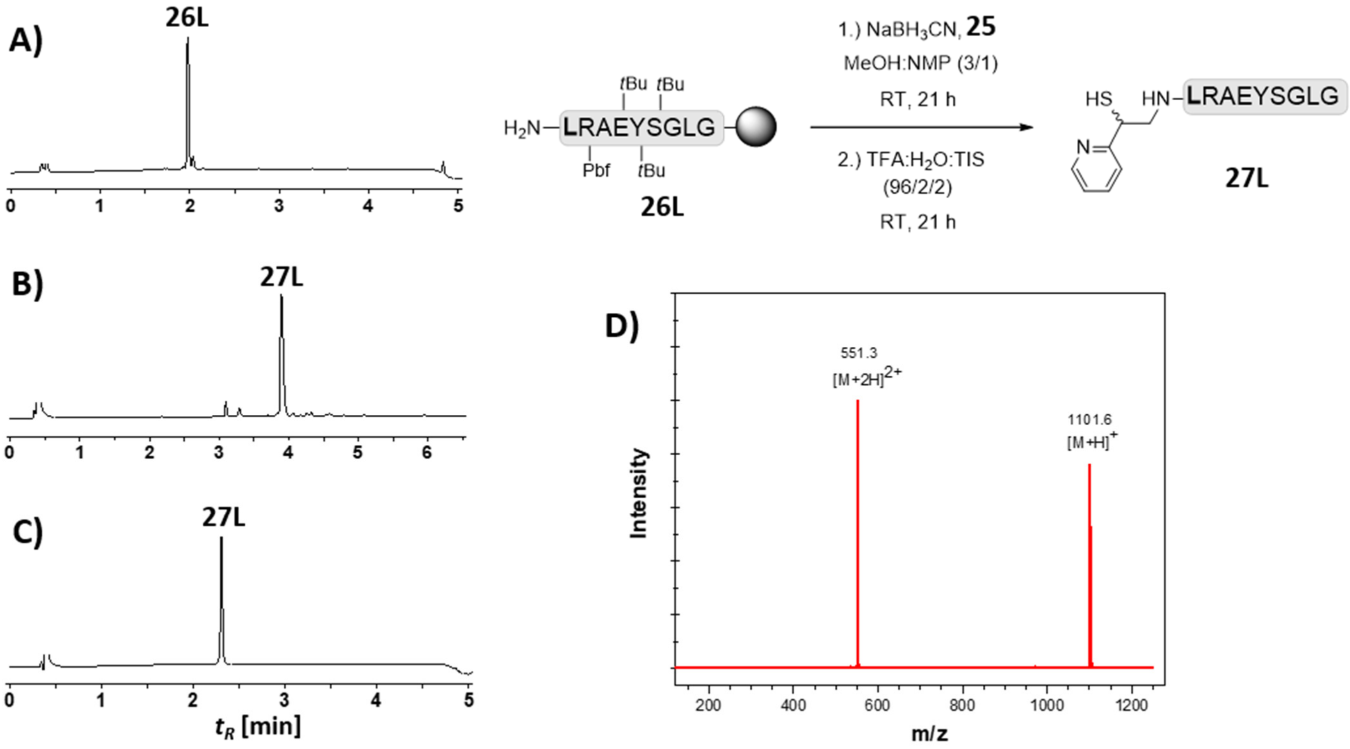

Figure S20: UPLC analysis of crude peptide obtained A) before and B) after reductive alkylation of **26L**. C) UPLC and D) MS analysis of purified product. UPLC analysis: A) and C) 3 - 30 % in 4 min, λ = 210 nm; B) 3 - 30 % in 6 min, λ = 210 nm.

6.2 Storage of Purified 2-Mercapto-2-(pyridin-2-yl)ethyl Auxiliary Peptides

MPyE auxiliary peptides decompose when stored in aqueous solution at pH range 2 – 6 at room temperature (see figure S21).[3] Preferably, the MPyE-peptides before ligation are stored in solution at -80 °C or as a lyophilizate at -20 °C or below.

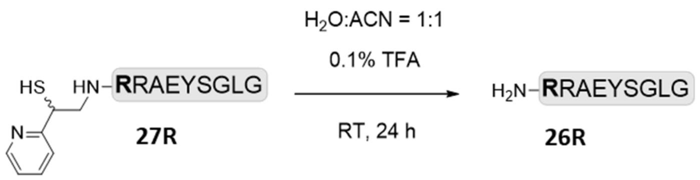

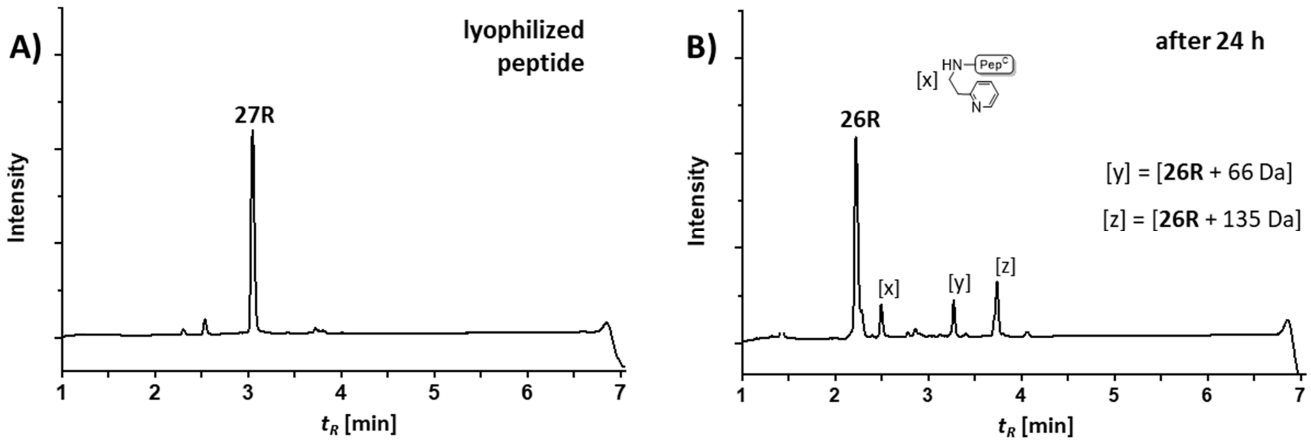

Figure S21: Auxiliary cleavage from peptide **27R** in acidic aqueous medium (pH ≃ 2). UPLC analysis: 3 - 30 % in 6 min, λ = 210 nm.

Auxiliary decomposition is not observed at pH < 1 or pH > 7. A suggested mechanism for this reaction is shown in figure S22.

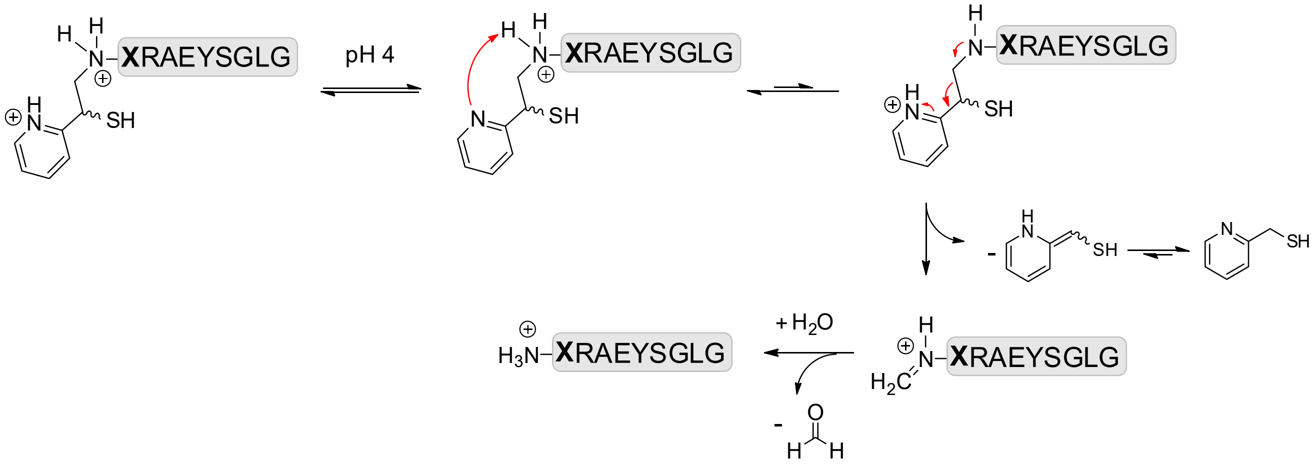

Figure S22: Postulated mechanism for the decomposition of MPyE-peptides at room temperature with pH 4.

7 Native Chemical Ligation

7.1 General Procedure for Ligation of MPyE-Peptides with Peptide Thioesters

Peptide thioester (1.5 eq.) and auxiliary peptide (1 eq.) were dissolved in the ligation buffer (200 mM sodium hydrogen phosphate, 6 M Gdn HCl, 20 mM TCEP, 3 vol.% thiophenol, pH = 7.5) to a final concentration of 5 mM auxiliary peptide. An argon atmosphere was applied to the reaction vessel. To monitor the progress of the reaction, aliquots were withdrawn from the ligation mixture, quenched with an aqueous solution of 0.1 % TFA or 0.1 % TFA, 2.5 % hydrazine, 30 mM TCEP and analyzed by UPLC-MS. The progress of the ligation reaction was assessed by integration of the corresponding peak areas. After completion of the ligation hydrazine and TCEP were added to the ligation solution which was subsequently purified by preparative HPLC as indicated. Some Peptides showed partial rearrangement back to the thioester intermediate due to the acidic conditions used during purification. To minimize rearrangement swift lyophilization after purification is advised.

Synthesis of Ligation Product AG (29AG)

500 nmol of MPyE-peptide **27G** and 1 µmol of peptide thioester **28A** were dissolved in 100 µL ligation buffer (see 7.1) and allowed to shake under argon atmosphere until completion of the ligation reaction (see figure S23). The crude ligation product was purified by preparative HPLC using a linear gradient from 3-30 % B in 40 min. The desired ligation product **29AG** was isolated as a white solid after lyophilization, which was dissolved in H_2O/ACN/TFA (1:1:0.001 v/v/v) for a spectroscopic determination of the synthesis yield (A_{280} = 0.39, V = 0.5 mL, 408 nmol, 82 %).

UPLC-MS: t_R = 2.89 min (3-30 % B in 4 min); m/z = 810.6 ($C_{72}H_{110}N_{22}O_{19}S$ $(M+2H)^{2+}$, calcd.: 810.4), 540.7 ($C_{72}H_{110}N_{22}O_{19}S$ $(M+3H)^{3+}$, calcd.: 540.6); $C_{72}H_{110}N_{22}O_{19}S$ (MW = 1618.8 g·mol^{-1}).

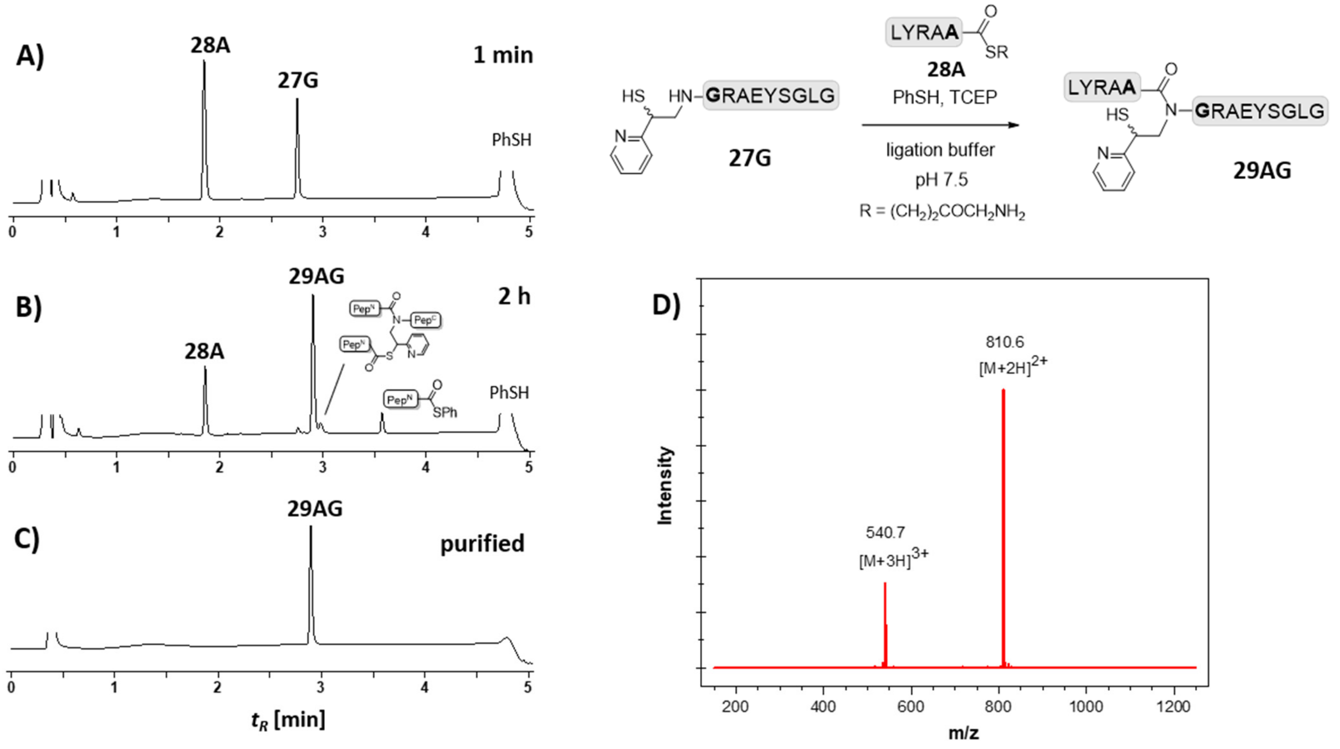

Figure S23: UPLC analysis of aliquots withdrawn at A) t = 1 min and B) t = 2 h (aliquots quenched with 0.1% TFA in H_2O). C) UPLC- and D) MS analysis of purified ligation product **29AG**. UPLC analysis: 3 - 30 % ACN in 4 min, λ = 210 nm. Pep^N = N-terminal fragment; Pep^C = C-terminal fragment.

Synthesis of Ligation Product LG (29LG)

1 µmol of MPyE-peptide **27G** and 2 µmol of peptide thioester **28L** were dissolved in 200 µL ligation buffer (see 7.1) and allowed to shake under argon atmosphere until completion of the ligation reaction (see figure S24). After quenching, the crude ligation product was purified by preparative HPLC using a linear gradient from 3-30 % B in 40 min. The desired ligation product **29LG** was isolated as a white solid after lyophilization, which was dissolved in H_2O/ACN/TFA (1:1:0.001 v/v/v) for a spectroscopic determination of the synthesis yield (A_{280} = 0.74, V = 0.5 mL, 782 nmol, 78 %).

UPLC-MS: t_R = 4.17 min (3-30 % B in 4 min); m/z = 831.6 ($C_{75}H_{116}N_{22}O_{19}S$ $(M+2H)^{2+}$, calcd.: 831.4), 554.7 ($C_{75}H_{116}N_{22}O_{19}S$ $(M+3H)^{3+}$, calcd.: 554.6); $C_{75}H_{116}N_{22}O_{19}S$ (MW = 1660.8 g·mol^{-1}).

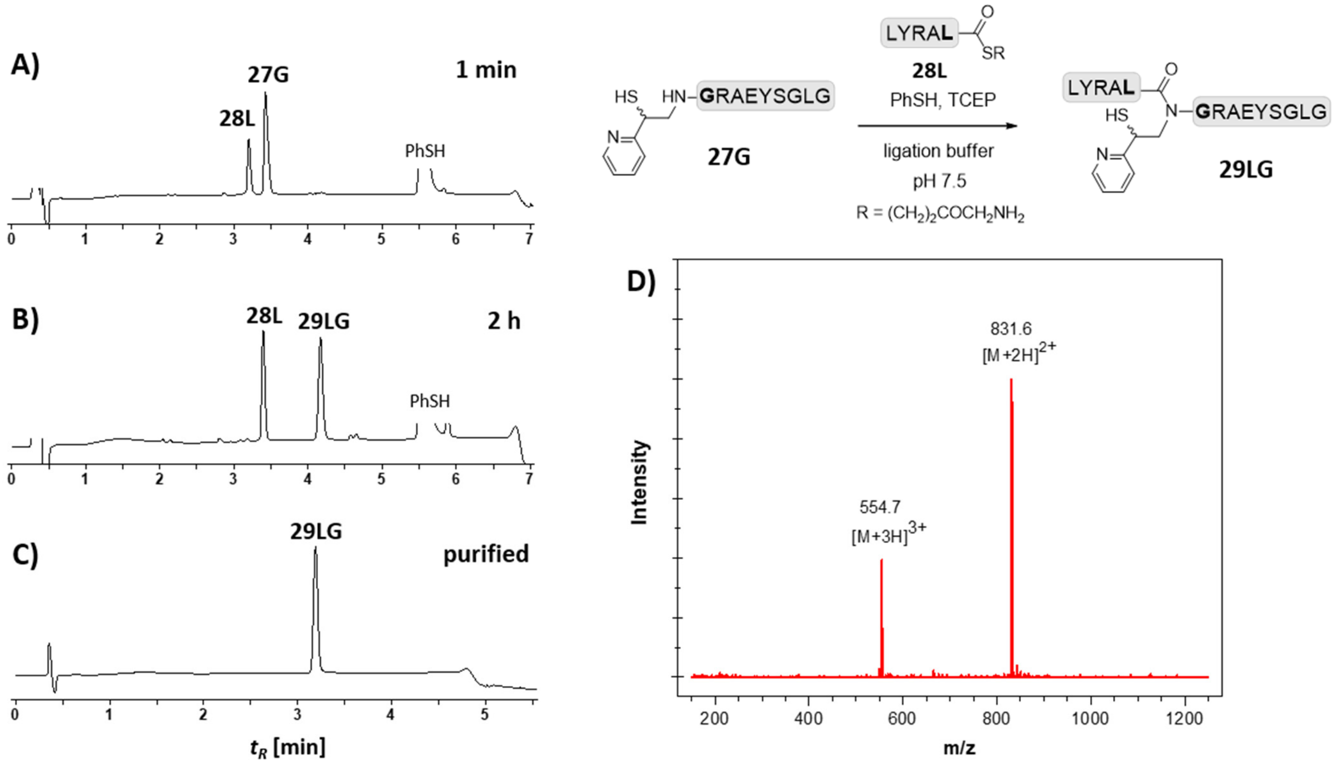

Figure S24: UPLC analysis of aliquots withdrawn at A) t = 1 min and B) t = 2 h (aliquots quenched with 0.1% TFA in H_2O). C) UPLC- and D) MS analysis of purified ligation product **29LG**. UPLC analysis: A) and B) 3 - 30 % ACN in 6 min, λ = 210 nm; C) 3 - 30 % ACN in 4 min, λ = 210 nm.

Synthesis of Ligation Product AN (29AN)

2 µmol of MPyE-peptide **27N** and 3 µmol of peptide thioester **28A** were dissolved in 400 µL ligation buffer (see 7.1) and allowed to shake under argon atmosphere until completion of the ligation reaction (see figure S25). The reaction was quenched by addition of 20 µL of 51 % aqueous hydrazine solution and subsequently 20 µL of 1 M TCEP solution. After quenching, the crude ligation product was purified by preparative HPLC using a linear gradient from 3-30 % B in 40 min. The desired ligation product **29AN** was isolated as a white solid after lyophilization, which was dissolved in H_2O/ACN/TFA (1:1:0.001 v/v/v) for a spectroscopic determination of the synthesis yield (A_{280} = 0.73, V = 1.0 mL, 1.55 µmol, 78 %).

UPLC-MS: t_R = 2.84 min (3-30 % B in 4 min); m/z = 839.2 ($C_{74}H_{113}N_{23}O_{20}S$ $(M+2H)^{2+}$, calcd.: 838.9), 559.8 ($C_{74}H_{113}N_{23}O_{20}S$ $(M+3H)^{3+}$, calcd.: 559.6); $C_{74}H_{113}N_{23}O_{20}S$ (MW = 1675.8 g·mol^{-1}).

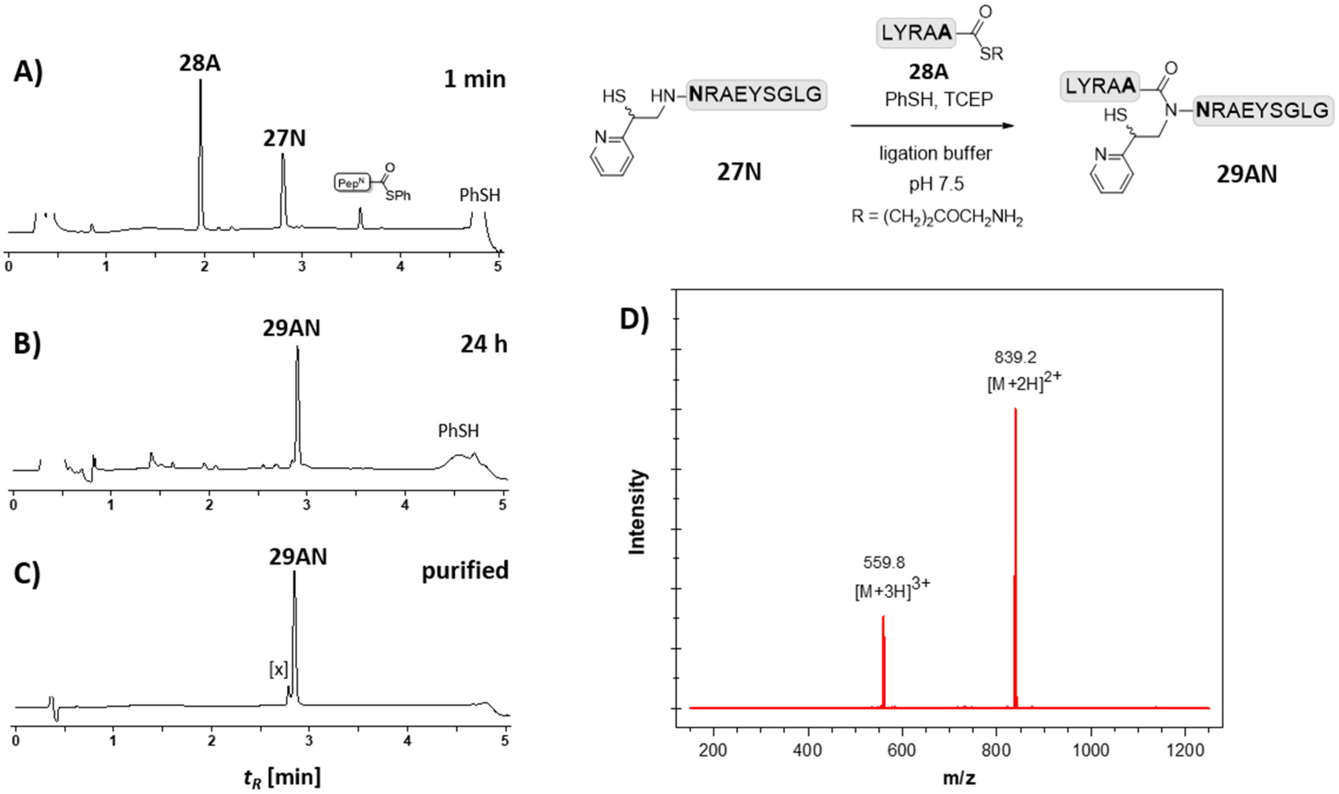

Figure S25: UPLC analysis of aliquots withdrawn at A) t = 1 min and B) t = 24 h (aliquots quenched with 0.1 % TFA, 2.5 % hydrazine and 30 mM TCEP in H_2O). C) UPLC- and D) MS analysis of purified ligation product **29AN**. UPLC analysis: 3 - 30 % ACN in 4 min, λ = 210 nm. Pep^N = N-terminal fragment. [x] thioester formed by N→S rearrangement.

Synthesis of Ligation Product LN (29LN)

2 µmol of MPyE-peptide **27N** and 3 µmol of peptide thioester **28L** were dissolved in 400 µL ligation buffer (see 7.1) and allowed to shake under argon atmosphere until completion of the ligation reaction (see figure S26). The reaction was quenched by addition of 20 µL of 51 % aqueous hydrazine solution and subsequently 20 µL of 1 M TCEP solution. After quenching, the crude ligation product was purified by preparative HPLC using a linear gradient from 3-30 % B in 40 min. The desired ligation product **29LN** was isolated as a white solid after lyophilization, which was dissolved in H_2O/ACN/TFA (1:1:0.001 v/v/v) for a spectroscopic determination of the synthesis yield (A_{280} = 0.67, V = 1.0 mL, 1.43 µmol, 72 %).

UPLC-MS: t_R = 3.20 min (3-30 % B in 4 min); m/z = 860.1 ($C_{77}H_{119}N_{23}O_{20}S$ $(M+2H)^{2+}$, calcd.: 859.9), 573.7 ($C_{77}H_{119}N_{23}O_{20}S$ $(M+3H)^{3+}$, calcd.: 573.6); $C_{77}H_{119}N_{23}O_{20}S$ (MW = 1717.9 g·mol^{-1}).

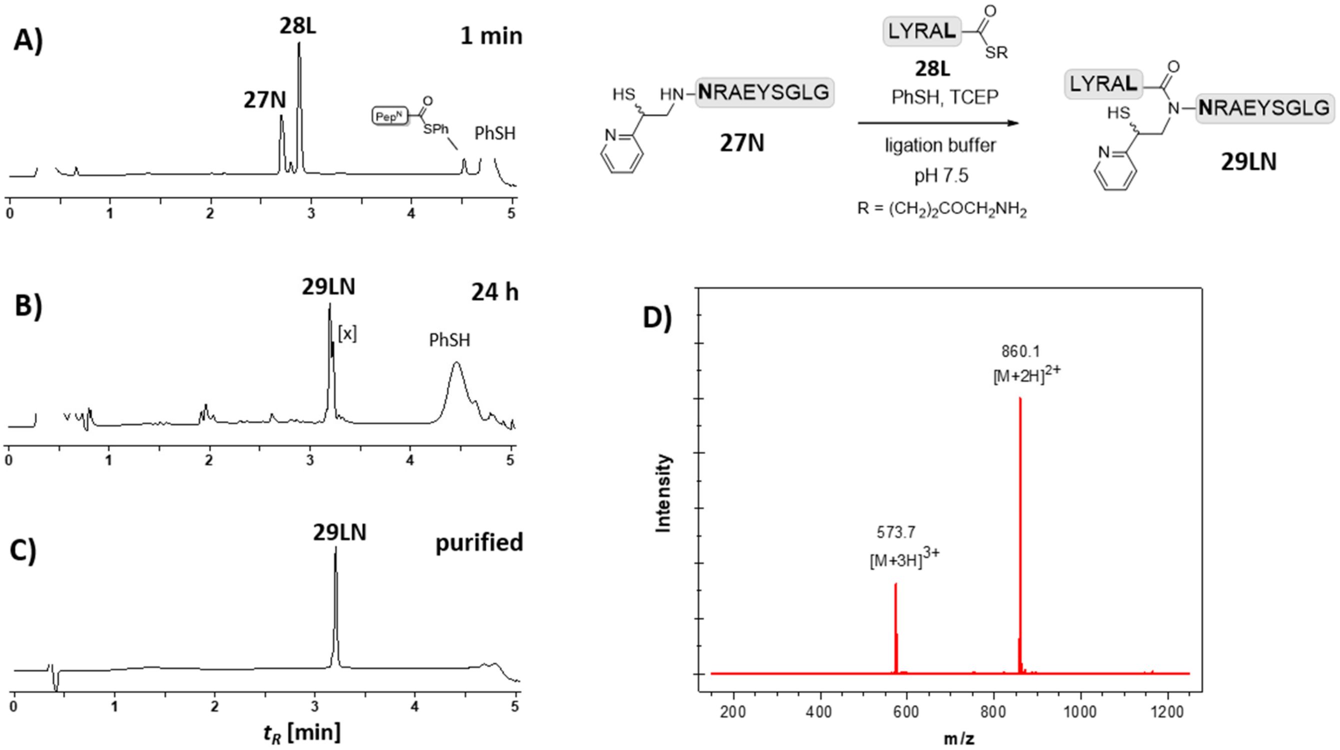

Figure S26: UPLC analysis of aliquots withdrawn at A) t = 1 min and B) t = 24 h (aliquots quenched with 0.1 % TFA, 2.5 % hydrazine and 30 mM TCEP in H_2O). C) UPLC- and D) MS analysis of purified ligation product **29LN**. UPLC analysis: 3 - 30 % ACN in 4 min, λ = 210 nm. Pep^N = N-terminal fragment. [x] stereoisomer of **29LN**.

Synthesis of Ligation Product AR (29AR)

2 µmol nmol of MPyE-peptide **27R** and 3 µmol of peptide thioester **28A** were dissolved in 400 µL ligation buffer (see 7.1) and allowed to shake under argon atmosphere until completion of the ligation reaction (see figure S27). The reaction was quenched by addition of 20 µL of 51 % aqueous hydrazine solution and subsequently 20 µL of 1 M TCEP solution. After quenching, the crude ligation product was purified by preparative HPLC using a linear gradient from 3-30 % B in 40 min. The desired ligation product **29AR** was isolated as a white solid after lyophilization, which was dissolved in H_2O/ACN/TFA (1:1:0.001 v/v/v) for a spectroscopic determination of the synthesis yield (A_{280} = 0.70, V = 1.0 mL, 1.47 µmol, 73 %).

UPLC-MS: t_R = 2.08 min (3-30 % B in 4 min); m/z = 860.4 ($C_{76}H_{119}N_{25}O_{19}S$ $(M+2H)^{2+}$, calcd.: 859.9), 573.9 ($C_{76}H_{119}N_{25}O_{19}S$ $(M+3H)^{3+}$, calcd.: 573.6); $C_{76}H_{119}N_{25}O_{19}S$ (MW = 1717.9 g·mol^{-1}).

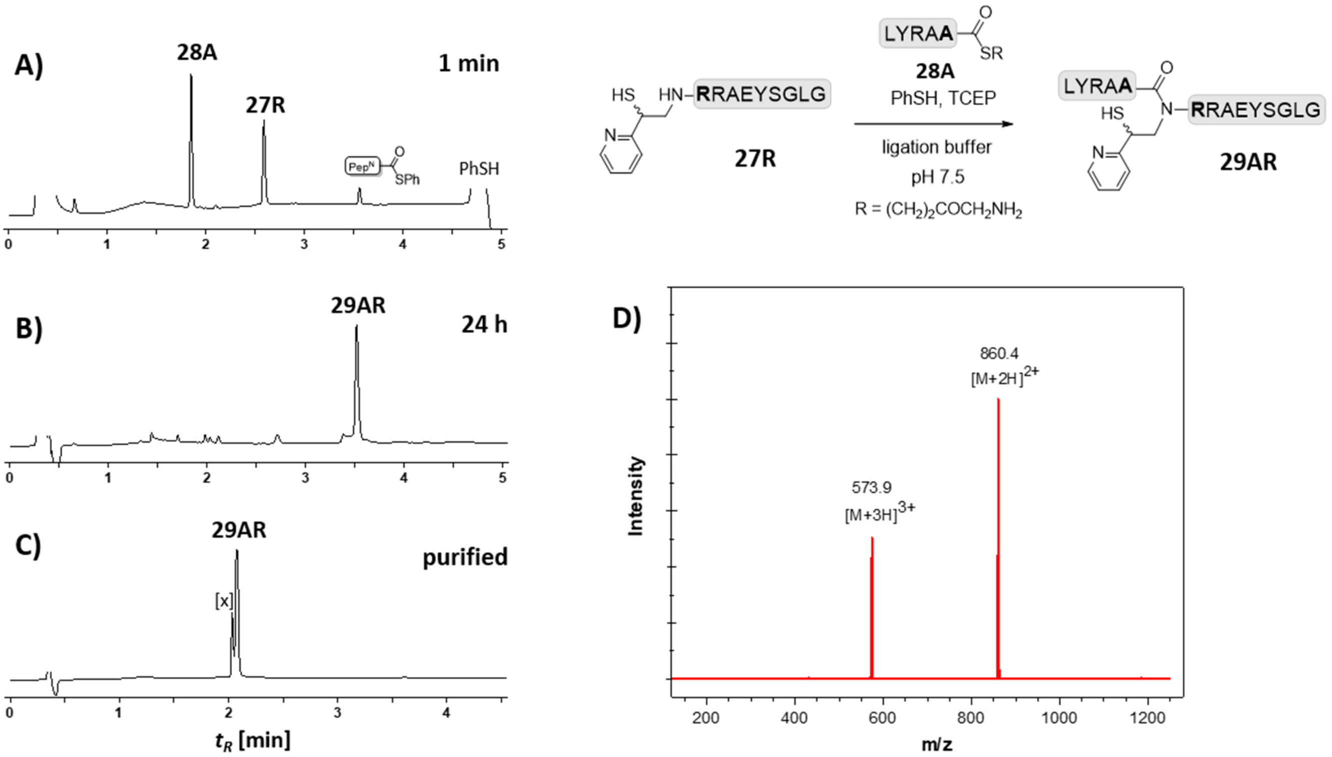

Figure S27: UPLC analysis of aliquots withdrawn at A) t = 1 min and B) t = 24 h (aliquots quenched with 0.1 % TFA, 2.5 % hydrazine and 30 mM TCEP in H_2O). C) UPLC- and D) MS analysis of purified ligation product **29AR**. UPLC analysis: A) and B) 3 - 30 % ACN in 6 min, λ = 210 nm; C) 3 - 30 % ACN in 4 min, λ = 210 nm. Pep^N = N-terminal fragment. [x] thioester formed by N→S rearrangement.

Synthesis of Ligation Product LR (29LR)

2 µmol of MPyE-peptide **27R** and 3 µmol of peptide thioester **28L** were dissolved in 400 µL ligation buffer (see 7.1) and allowed to shake under argon atmosphere until completion of the ligation reaction (see figure S28). The reaction was quenched by addition of 20 µL of 51 % aqueous hydrazine solution and subsequently 20 µL of 1 M TCEP solution. After quenching, the crude ligation product was purified by preparative HPLC using a linear gradient from 3-30 % B in 40 min. The desired ligation product **29LR** was isolated as a white solid after lyophilization, which was dissolved in H_2O/ACN/TFA (1:1:0.001 v/v/v) for a spectroscopic determination of the synthesis yield (A_{280} = 0.62, V = 1.0 mL, 1.32 µmol, 66 %).

UPLC-MS: t_R = 3.08 min (3-30 % B in 4 min); m/z = 881.5 ($C_{79}H_{125}N_{25}O_{19}S$ $(M+2H)^{2+}$, calcd.: 880.9), 588.0 ($C_{79}H_{125}N_{25}O_{19}S$ $(M+3H)^{3+}$, calcd.: 587.6); $C_{79}H_{125}N_{25}O_{19}S$ (MW = 1759.9 g·mol^{-1}).

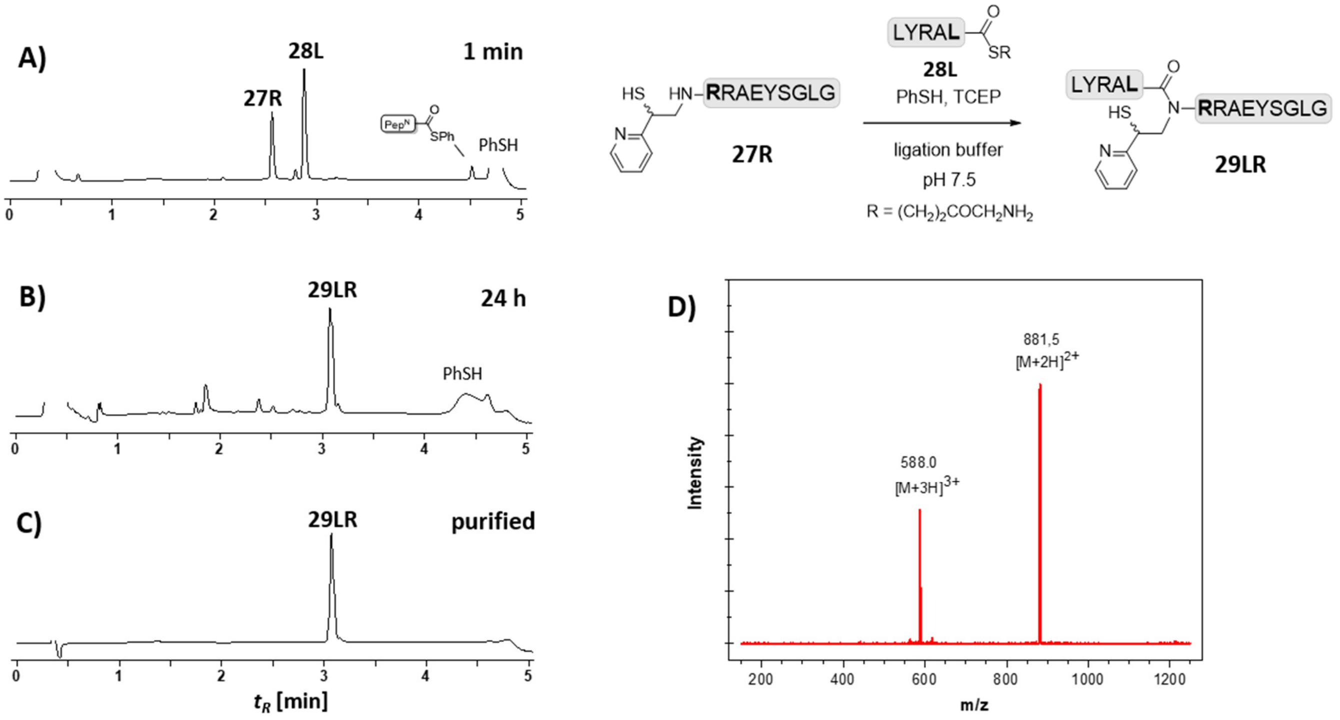

Figure S28: UPLC analysis of aliquots withdrawn at A) t = 1 min and B) t = 24 h (aliquots quenched with 0.1 % TFA, 2.5 % hydrazine and 30 mM TCEP in H_2O). C) UPLC- and D) MS analysis of purified ligation product **29LR**. UPLC analysis: 3 - 30 % ACN in 4 min, λ = 210 nm. Pep^N = N-terminal fragment.

Synthesis of Ligation Product AV (29AV)

2 µmol of MPyE-peptide **27V** and 3 µmol of peptide thioester **28A** were dissolved in 400 µL ligation buffer (see 7.1) and allowed to shake under argon atmosphere until completion of the ligation reaction (see figure S29). The reaction was quenched by addition of 20 µL of 51 % aqueous hydrazine solution and subsequently 20 µL of 1 M TCEP solution. After quenching, the crude ligation product was purified by preparative HPLC using a linear gradient from 3-30 % B in 40 min. The desired ligation product **29AV** was isolated as a white solid after lyophilization, which was dissolved in H_2O/ACN/TFA (1:1:0.001 v/v/v) for a spectroscopic determination of the synthesis yield (A_{280} = 0.55, V = 1.0 mL, 1.16 µmol, 58 %).

UPLC-MS: t_R = 2.32 min (3-30 % B in 4 min); m/z = 831.6 ($C_{75}H_{116}N_{22}O_{19}S$ $(M+2H)^{2+}$, calcd.: 831.4), 554.7 ($C_{75}H_{116}N_{22}O_{19}S$ $(M+3H)^{3+}$, calcd.: 554.6); $C_{79}H_{125}N_{25}O_{19}S$ (MW = 1660.8 g·mol^{-1}).

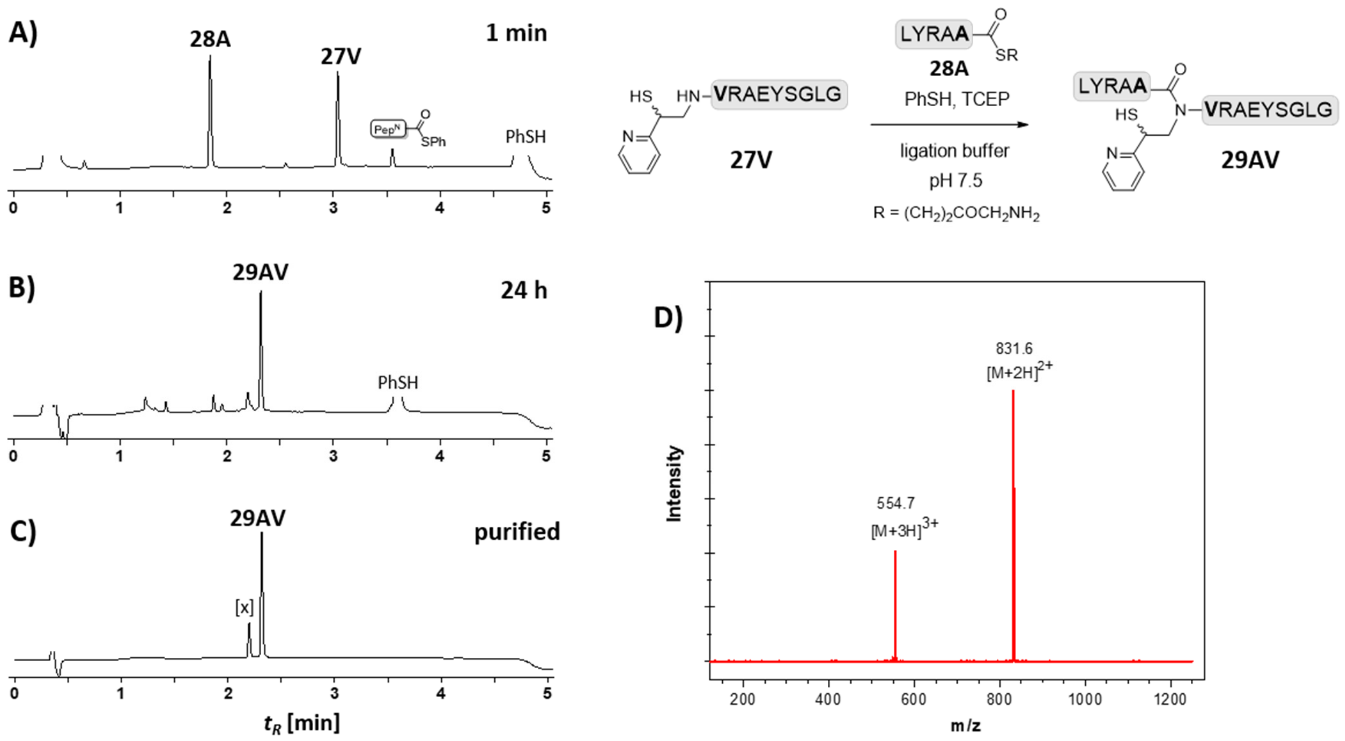

Figure S29: UPLC analysis of aliquots withdrawn at A) t = 1 min and B) t = 24 h (aliquots quenched with 0.1 % TFA, 2.5 % hydrazine and 30 mM TCEP in H_2O). C) UPLC- and D) MS analysis of purified ligation product **29AV**. UPLC analysis: 3 - 30 % ACN in 4 min, λ = 210 nm. Pep^N = N-terminal fragment. [x] thioester formed by N→S rearrangement.

Synthesis of Ligation Product AL (29AL)

1 µmol of MPyE-peptide **27L** and 1.5 µmol of peptide thioester **28A** were dissolved in 200 µL ligation buffer (see 7.1) and allowed to shake under argon atmosphere until completion of the ligation reaction (see figure S30). The reaction was quenched by addition of 10 µL of 51 % aqueous hydrazine solution and subsequently 10 µL of 1 M TCEP solution. After quenching, the crude ligation product was purified by preparative HPLC using a linear gradient from 3-30 % B in 40 min. The desired ligation product **29AL** was isolated as a white solid after lyophilization, which was dissolved in H_2O/ACN/TFA (1:1:0.001 v/v/v) for a spectroscopic determination of the synthesis yield (A_{280} = 0.40, V = 0.5 mL, 421 nmol, 42 %).

UPLC-MS: t_R = 4.85 min (3-30 % B in 6 min); m/z = 838.7 ($C_{76}H_{118}N_{22}O_{19}S$ $(M+2H)^{2+}$, calcd.: 838.4), 559.4 ($C_{76}H_{118}N_{22}O_{19}S$ $(M+3H)^{3+}$, calcd.: 559.3); $C_{76}H_{118}N_{22}O_{19}S$ (MW = 1674.9 g·mol^{-1}).

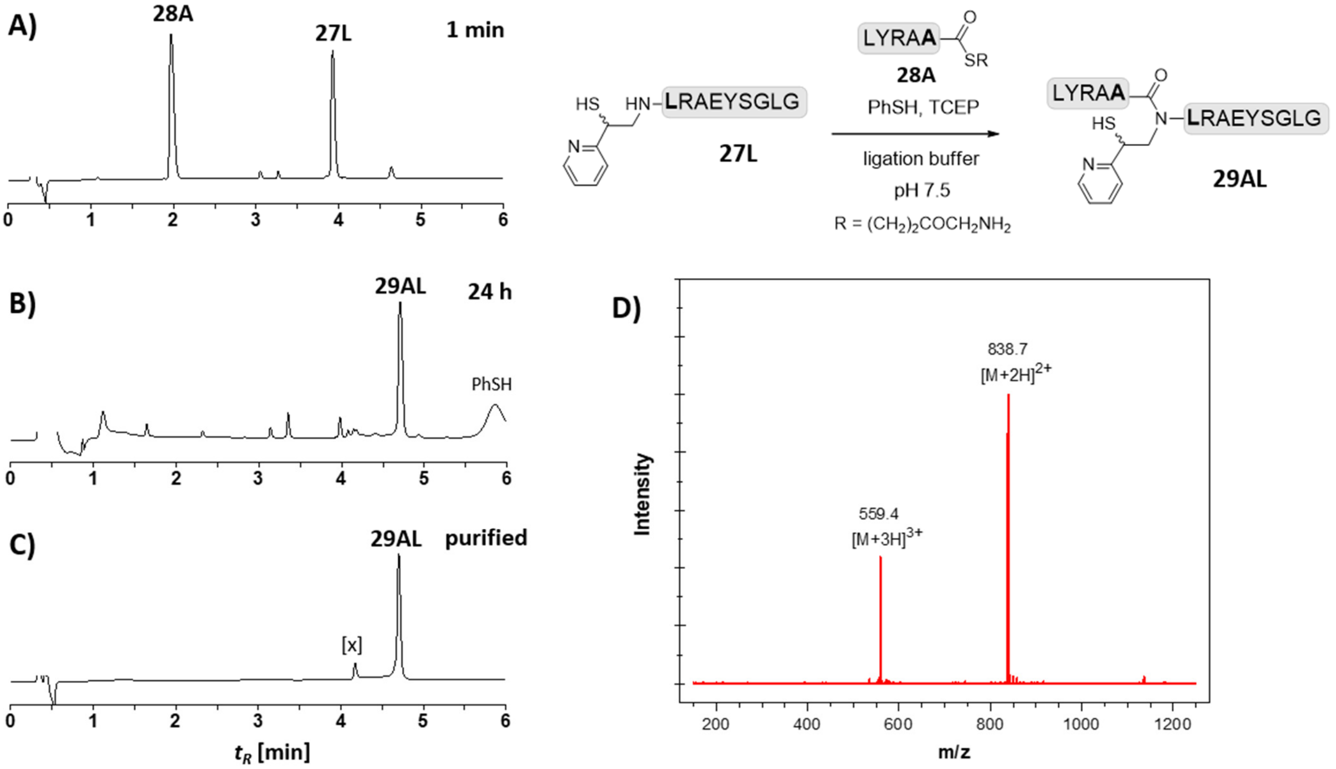

Figure S30: UPLC analysis of aliquots withdrawn at A) t = 1 min and B) t = 24 h (aliquots quenched with 0.1 % TFA, 2.5 % hydrazine and 30 mM TCEP in H_2O). C) UPLC- and D) MS analysis of purified ligation product **29AL**. UPLC analysis: 3 - 30 % ACN in 6 min, λ = 210 nm. [x] thioester formed by N→S rearrangement.

8 Comparison between MPyE- and MPE-peptides in ligation reactions with peptide selenoester

8.1 Ligation of MPyE-peptides with Peptide Selenoesters

1 μmol MPyE-peptide and 2 μmol of peptide selenoester were dissolved in the ligation buffer (200 mM sodium hydrogen phosphate, 6 M Gdn HCl, 100 mM TCEP, 28 mM DPDS, pH = 6.2) to a final concentration of 5 mM in relation to the MPyE-peptide. A light argon atmosphere was applied to the reaction vessel. To monitor the progress of the reaction, aliquots were withdrawn from the ligation mixture, quenched with an aqueous solution of 0.1 % TFA or 0.1 % TFA, 2.5 % hydrazine, 30 mM TCEP and analyzed by UPLC-MS.

Please note: reactions described in 7.1 were performed in order to assess mechanistic details. For preparative ligations with peptide selenosters the reaction time should be optimized.

Comparison at the AN Junction

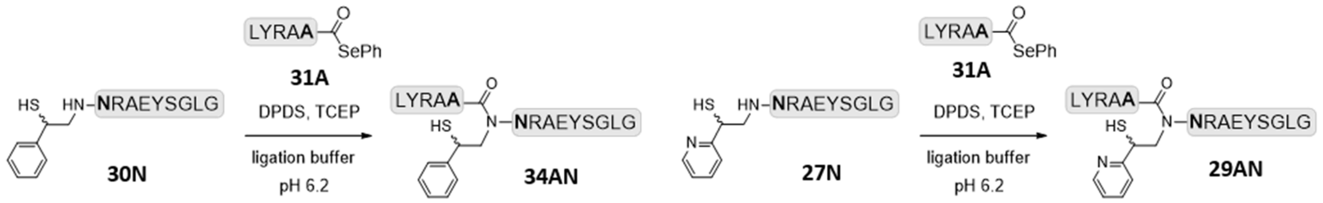

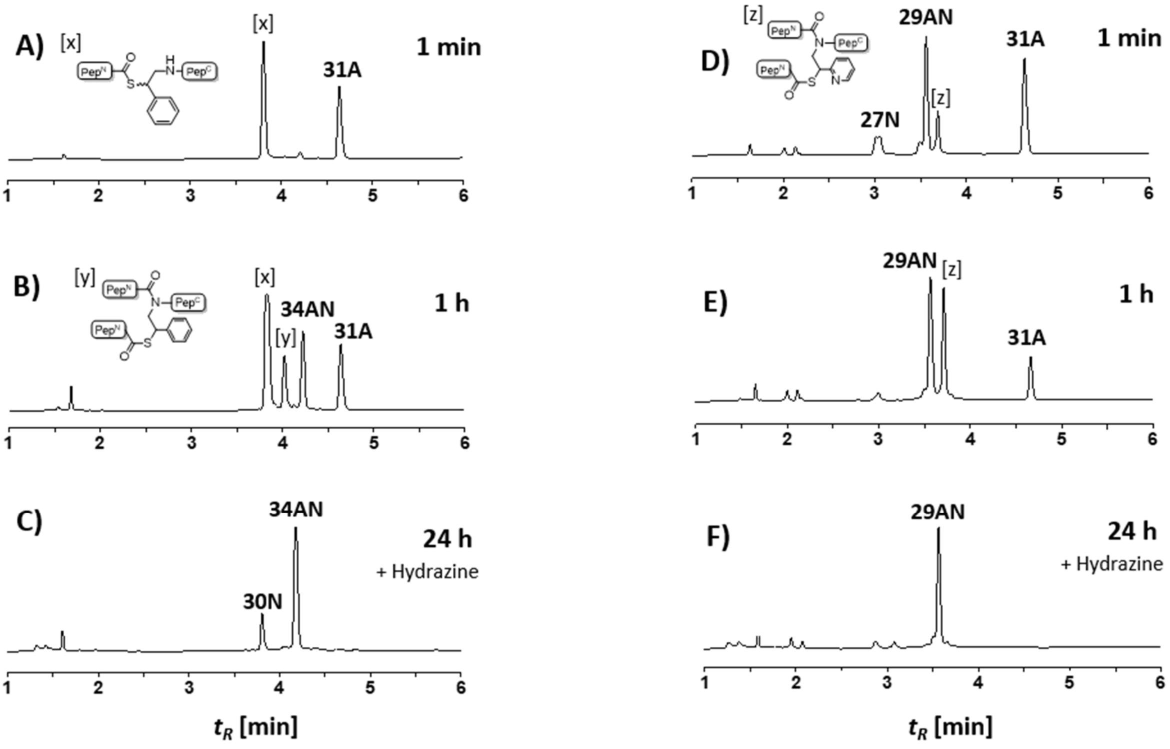

Figure S31: Ligation at the AN-junction with A), B), C) MPE-peptide or D), E), F) MPyE-peptide at A), D) t = 1 min, B), E) t = 1 h and after C), F) t = 24 h (aliquots quenched with 0.1 % TFA, 2.5 % hydrazine and 30 mM TCEP in H_2O). UPLC analysis: 3 - 30 % ACN in 6 min, λ = 210 nm. Pep^N = N-terminal fragment; Pep^C = C-terminal Fragment.

Comparison at the LN Junction

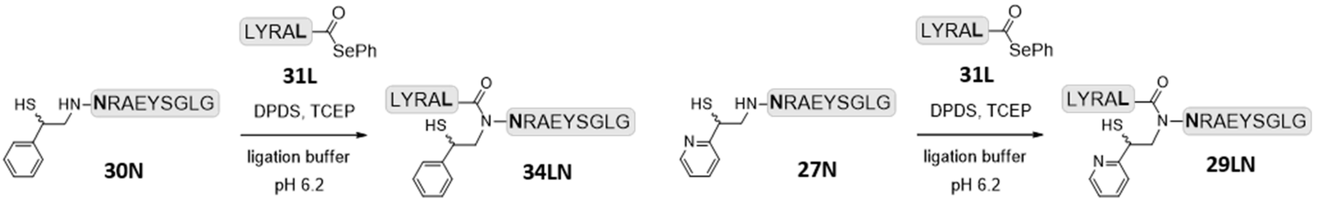

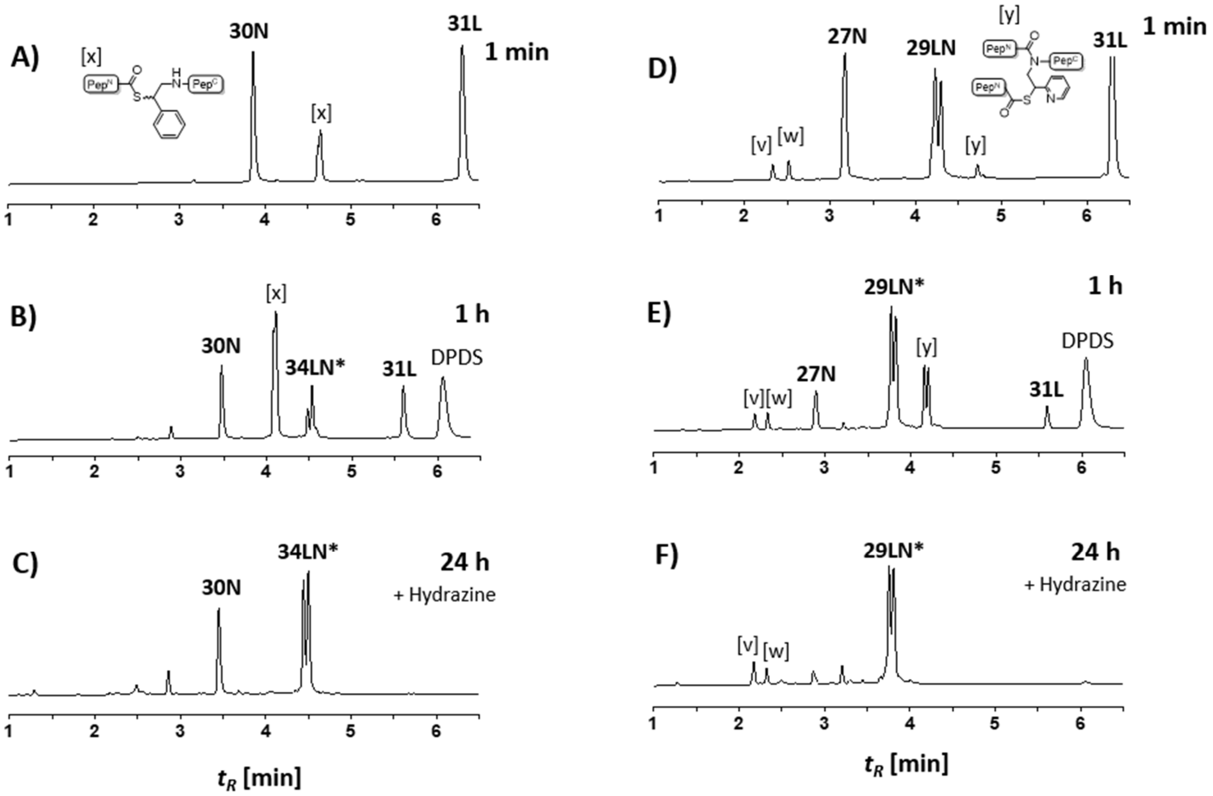

Figure S32: Ligation at the LN-junction with A), B), C) MPE-peptide or D), E), F) MPyE-peptide at A), D) t = 1 min, B), E) t = 1 h and after C), F) t = 24 h (aliquots quenched with 0.1 % TFA, 2.5 % hydrazine and 30 mM TCEP in H_2O). UPLC analysis: A) und D) 3 - 30 % ACN in 6 min, λ = 210 nm; B), C), E), F) 3 - 35 % ACN in 6 min, λ = 210 nm. Pep^N = N-terminal fragment; Pep^C = C-terminal Fragment. [v] desulfurized **27N**; [w] **27N** with cleaved auxiliary. *the two auxiliary stereoisomers elute as a double peak.

Comparison at the LV Junction

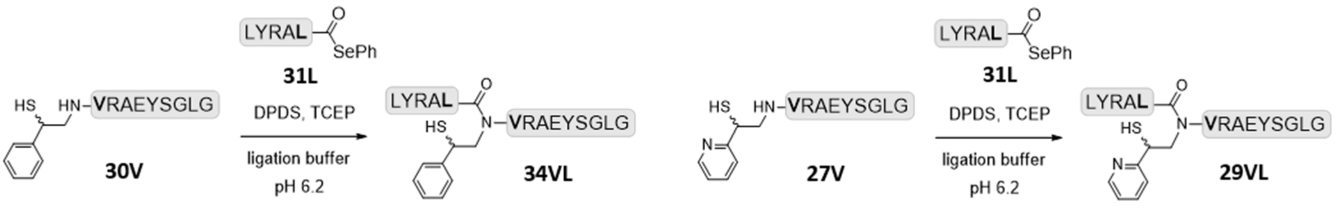

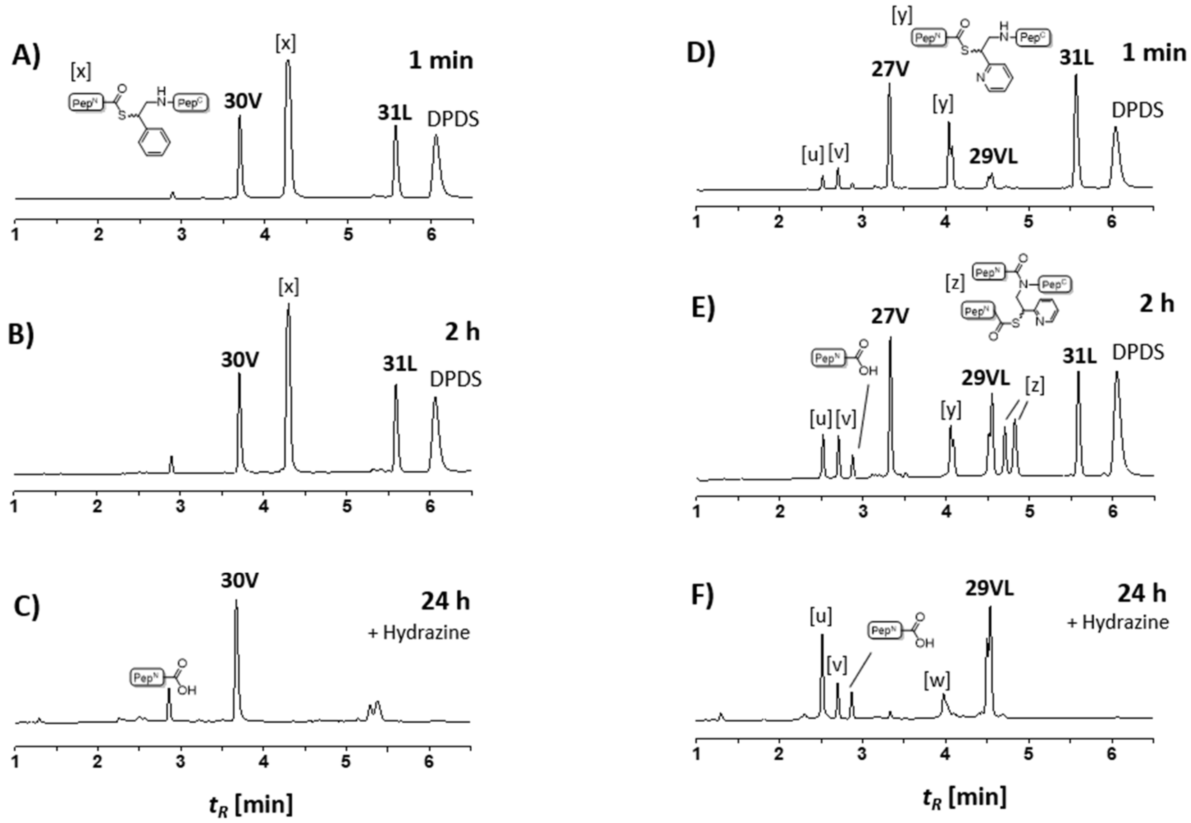

Figure S33: Ligation at the LV-junction with A), B), C) MPE-peptide or D), E), F) MPyE-peptide at A), D) t = 1 min, B), E) t = 2 h and after C), F) t = 24 h (aliquots quenched with 0.1 % TFA, 2.5 % hydrazine and 30 mM TCEP in H_2O). UPLC analysis: 3 - 35 % ACN in 6 min, λ = 210 nm. Pep^N = N-terminal fragment; Pep^C = C-terminal Fragment. [v] desulfurized **27V**; [w] **27V** with cleaved auxiliary; [x] the two stereoisomers of elute as a double peak.

9 Comparison between Proline Seleno- and Proline Thioester in Ligation with a MPyE-Peptide

The MPyE-peptide **27R** was reacted with peptide thioester following the general procedure 7.1 and with the peptide selenoester **31P** following the general procedure 8.1.

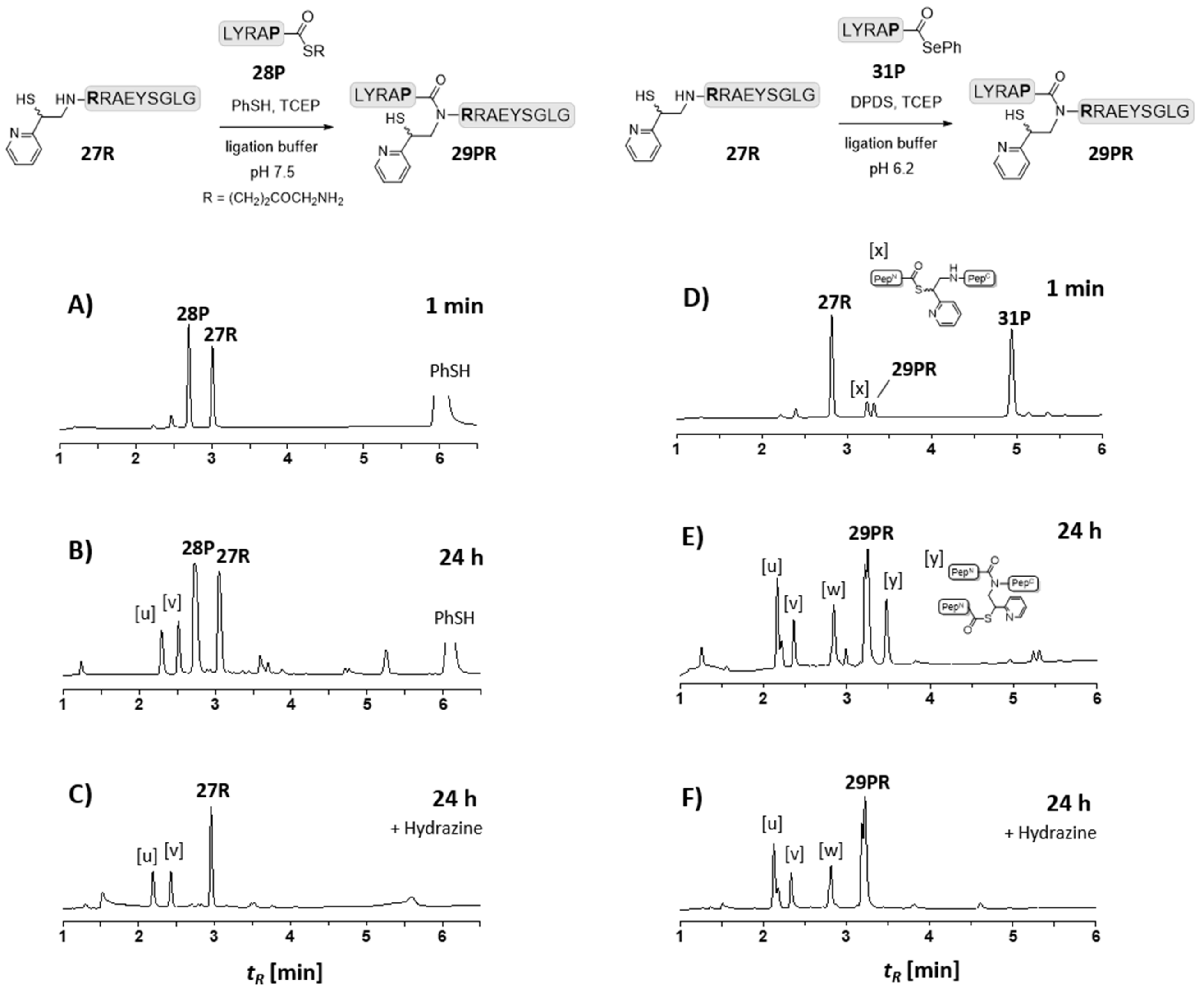

Figure S34: Ligation at the PR-junction with A), B), C) proline thioester or D), E), F) proline selenoester at A), D) t = 1 min, B), E) t = 2 h and after C), F) t = 24 h (aliquots quenched with 0.1 % TFA, 2.5 % hydrazine and 30 mM TCEP in H_2O). UPLC analysis: 3 - 30 % ACN in 6 min, λ = 210 nm. Pep^N = N-terminal fragment; Pep^C = C-terminal Fragment. [u] **27R** with cleaved auxiliary; [v] desulfurized **27R**; [w] desulfurized **29PR**.

10 Auxiliary Removal

10.1 General Procedure for Removal of the MPyE Auxiliary

The lyophilized ligation product was dissolved in the auxiliary cleavage mixture (0.5 M TCEP, 2 M amine base, 5 mM $MnCl_2$, 0.5 M 2-acetylpyridine, pH = 8,5) to a final concentration of 1 mM. For compounds containing a Gly at the junction, piperazine was chosen as the amine base. For all other ligation sites, morpholine was chosen as the corresponding base. The reaction mixture was shaken in an Eppendorf tube. The closed lid was punctured to allow supply of oxygen. To monitor the progress of the reaction, aliquots were withdrawn from the ligation mixture and analyzed by UPLC-MS. The progress of the ligation reaction was assessed by integration of the corresponding peak areas. After completion, the reaction product was purified by preparative HPLC as indicated.

We assume that the mechanism of cleavage is comparable to that of the MPE auxiliary (see figure S35).

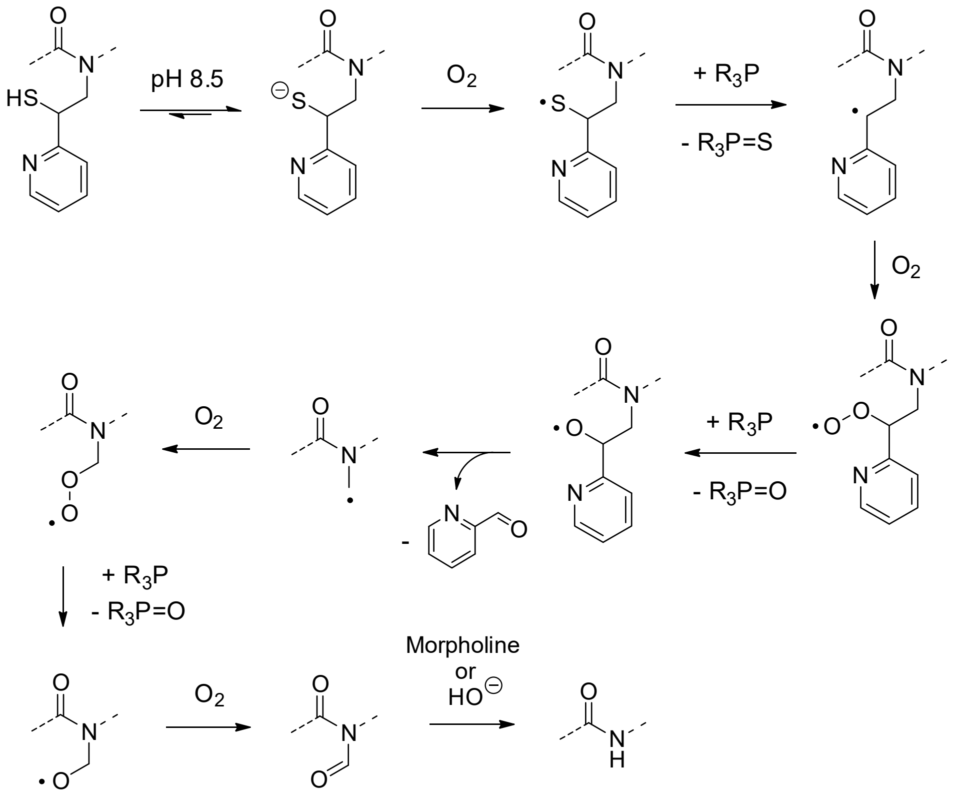

Figure S35: Putative reaction mechanism for the cleavage of MPyE auxiliary from a ligated peptide.

Removal of Auxiliary from Ligation Product AG (37AG)

To 315 nmol of ligated peptide **29AG,** 315 µL auxiliary cleavage mixture was added (see 10.1). The mixture was shaken for 3 h and the crude ligation product was purified by preparative HPLC rinsing initially for 10 minutes at 15 % B and subsequently using a linear gradient from 15-30 % B in 30 min. The desired auxiliary removal product **37AG** was isolated as a white solid after lyophilization (see figure S36), which was dissolved in H_2O/ACN/TFA (1:1:0.001 v/v/v) for a spectroscopic determination of the synthesis yield (A_{280} = 0.17, V = 0.5 mL, 279 nmol, 88 %).

UPLC-MS: t_R = 2.43 min (3-30 % B in 4 min); m/z = 742.2 ($C_{65}H_{103}N_{21}O_{19}$ $(M+2H)^{2+}$, calcd.: 741.9), 495.2 ($C_{65}H_{103}N_{21}O_{19}$ $(M+3H)^{3+}$, calcd.: 494.9); $C_{65}H_{103}N_{21}O_{19}$ (MW = 1481.8 g· mol^{-1}).

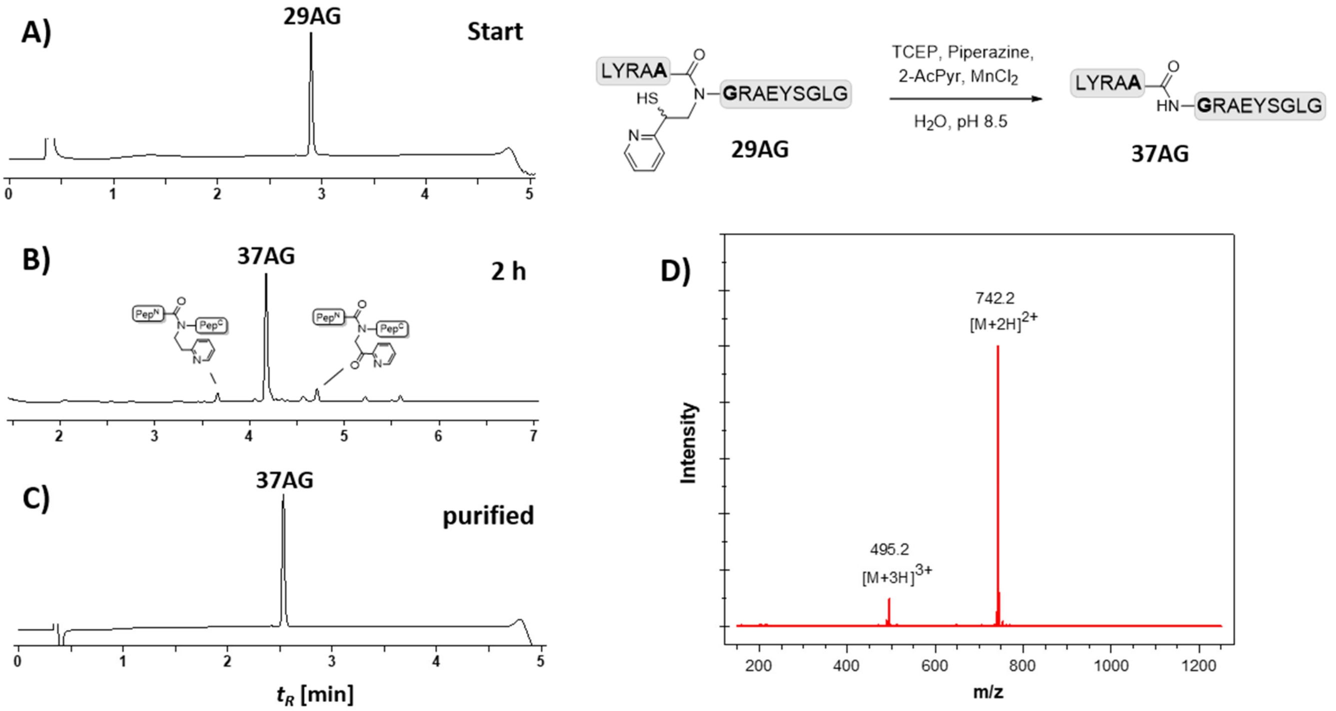

Figure S36: UPLC analysis of auxiliary removal. A) ligation product **29AG**, B) crude material after 2 h reaction and C) purified peptide **37AG**. D) ESI-MS of purified **37AG**. UPLC analysis: A) and C) 3 - 30 % ACN in 4 min, λ = 210 nm; B) 3 % ACN for 1 min, then 3 - 30 % in 6 min, λ = 210 nm. Pep^N = N-terminal fragment; Pep^C = C-terminal fragment.

Removal of Auxiliary from Ligation Product LG (37LG)

To 255 nmol of ligated peptide **29LG,** 255 µL auxiliary cleavage mixture was added (see 10.1). The mixture was shaken for 3 h and the crude ligation product was purified by preparative HPLC using a linear gradient from 3-30 % B in 40 min. The desired auxiliary removal product **37LG** was isolated as a white solid after lyophilization (see figure S37), which was dissolved in H_2O/ACN/TFA (1:1:0.001 v/v/v) for a spectroscopic determination of the synthesis yield (A_{280} = 0.08, V = 0.5 mL, 138 nmol, 54 %).

UPLC-MS: t_R = 3.02 min (3-30 % B in 4 min); m/z = 763.3 ($C_{68}H_{109}N_{21}O_{19}$ $(M+2H)^{2+}$, calcd.: 762.9), 509.2 ($C_{68}H_{109}N_{21}O_{19}$ $(M+3H)^{3+}$, calcd.: 508.9); $C_{68}H_{109}N_{21}O_{19}$ (MW = 1523.8 g· mol^{-1}).

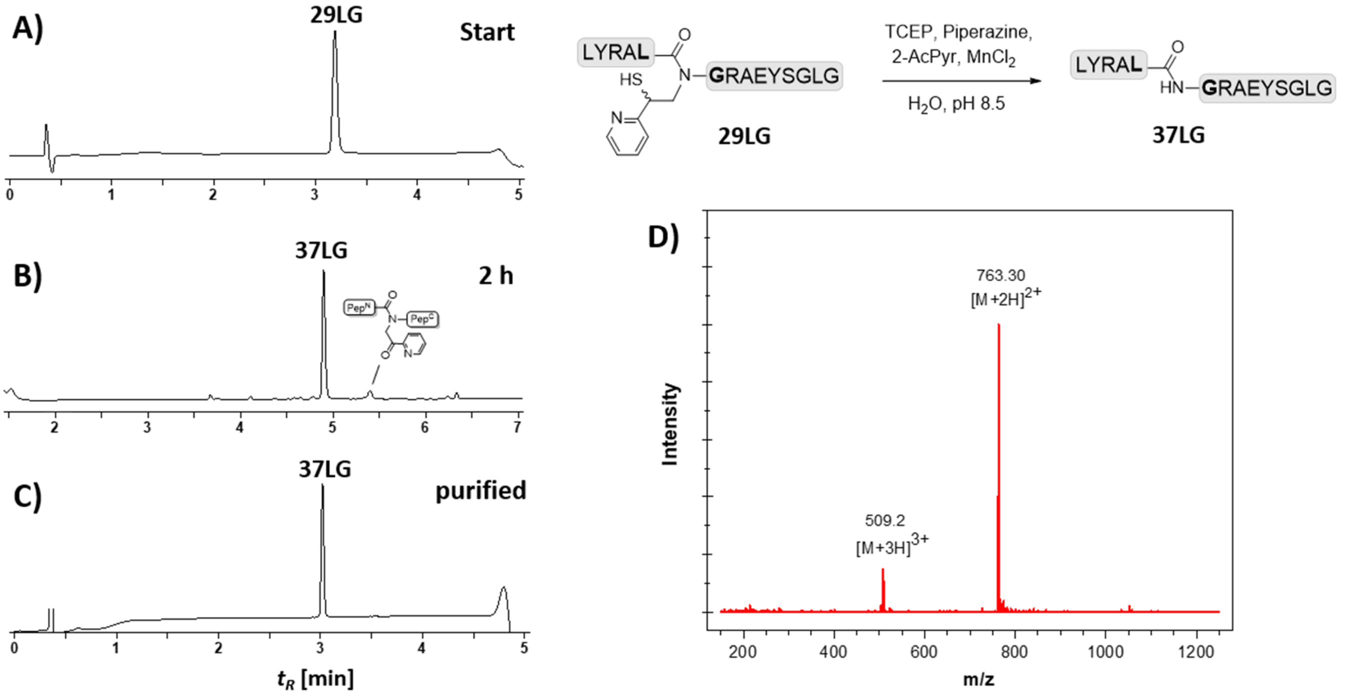

Figure S37: UPLC analysis of auxiliary removal. A) ligation product **29LG**, B) crude material after 2 h reaction and C) purified peptide **37LG**. D) ESI-MS of purified **37LG**. UPLC analysis: A) and C) 3 - 30 % ACN in 4 min, λ = 210 nm; B) 3 % ACN for 1 min, then 3 - 30 % in 6 min, λ = 210 nm. Pep^N = N-terminal fragment; Pep^C = C-terminal fragment.

Removal of Auxiliary from Ligation Product AN (37AN)

To 175 nmol of ligated peptide **29AN,** 175 µL auxiliary cleavage mixture was added (see 10.1). The mixture was shaken for 24 h and the crude ligation product was purified by preparative HPLC rinsing initially for 10 minutes at 15 % B and subsequently using a linear gradient from 15-30 % B in 30 min. The desired auxiliary removal product **37AN** was isolated as a white solid after lyophilization (see figure S38), which was dissolved in H_2O/ACN/TFA (1:1:0.001 v/v/v) for a spectroscopic determination of the synthesis yield (A_{280} = 0.06, V = 0.5 mL, 97 nmol, 55 %).

UPLC-MS: t_R = 2.51 min (3-30 % B in 4 min); m/z = 770.2 ($C_{67}H_{106}N_{22}O_{20}$ $(M+2H)^{2+}$, calcd.: 770.4), 514.3 ($C_{67}H_{106}N_{22}O_{20}$ $(M+3H)^{3+}$, calcd.: 513.9); $C_{67}H_{106}N_{22}O_{20}$ (MW = 1538.8 g· mol^{-1}).

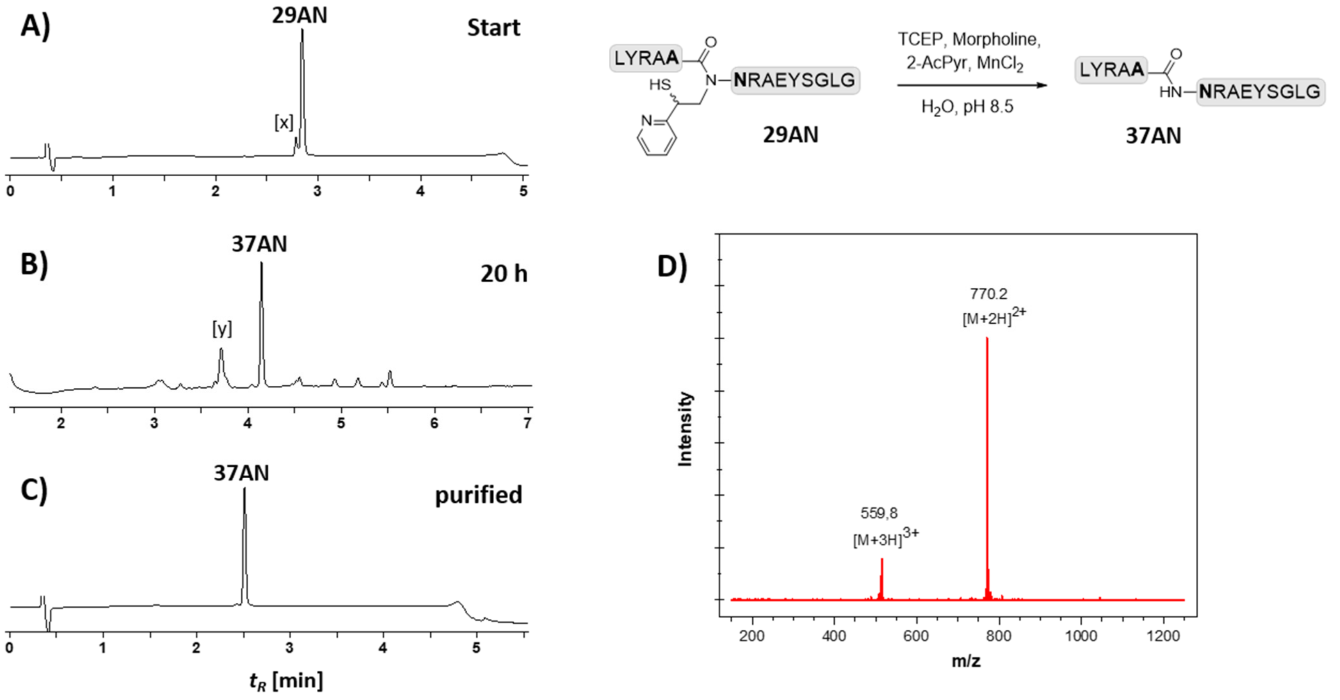

Figure S38: UPLC analysis of auxiliary removal. A) ligation product **29AN**, B) crude material after 20 h reaction and C) purified peptide **37AN**. D) ESI-MS of purified **37AN**. UPLC analysis: A) and C) 3 - 30 % ACN in 4 min, λ = 210 nm; B) 3 % ACN for 1 min, then 3 - 30 % in 6 min, λ = 210 nm. [x] thioester form by N→S rearrangement; [y] nonpeptidic material (m/z = 475).

Removal of Auxiliary from Ligation Product AR (37AR)

To 315 nmol of ligated peptide **29AR,** 315 µL auxiliary cleavage mixture was added (see 10.1). The mixture was shaken for 24 h and the crude ligation product was purified by preparative HPLC rinsing initially for 10 minutes at 15 % B and subsequently using a linear gradient from 15-30 % B in 30 min. The desired auxiliary removal product **37AR** was isolated as a white solid after lyophilization (see figure S39), which was dissolved in H_2O/ACN/TFA (1:1:0.001 v/v/v) for a spectroscopic determination of the synthesis yield (A_{280} = 0.09, V = 0.5 mL, 149 nmol, 47 %).

UPLC-MS: t_R = 2.44 min (3-30 % B in 4 min); m/z = 791.6 ($C_{69}H_{112}N_{24}O_{19}$ $(M+2H)^{2+}$, calcd.: 791.4), 528.1 ($C_{69}H_{112}N_{24}O_{19}$ $(M+3H)^{3+}$, calcd.: 527.9); $C_{69}H_{112}N_{24}O_{19}$ (MW = 1580.8 g· mol^{-1}).

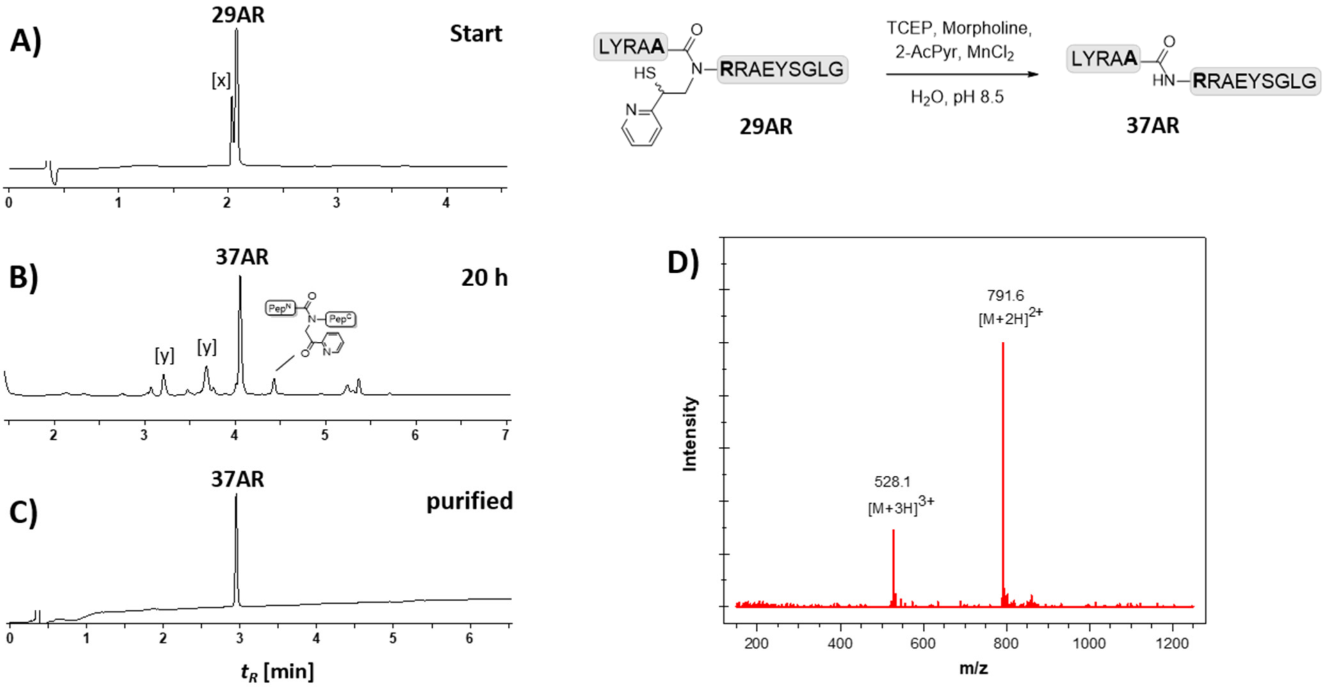

Figure S39: UPLC analysis of auxiliary removal. A) ligation product **29AR**, B) crude material after 20 h reaction and C) purified peptide **37AR**. D) ESI-MS of purified **37AR**. UPLC analysis: A) 3 - 30 % ACN in 4 min, λ = 210 nm; B) 3 % ACN for 1 min, then 3 - 30 % in 6 min, λ = 210 nm; C) 3 - 30 % ACN in 6 min, λ = 210 nm. Pep^N = N-terminal fragment; Pep^C = C-terminal fragment. [x] thioester formed by N→S rearrangement; [y] nonpeptidic material.

Removal of Auxiliary from Ligation Product AV (37AV)

To 510 nmol of ligated peptide **29AV,** 510 µL auxiliary cleavage mixture was added (see 10.1). The mixture was shaken for 24 h and the crude ligation product was purified by preparative HPLC rinsing initially for 10 minutes at 15 % B and subsequently using a linear gradient from 15-30 % B in 30 min. The desired auxiliary removal product **37AV** was isolated as a white solid after lyophilization (see figure S40), which was dissolved in H_2O/ACN/TFA (1:1:0.001 v/v/v) for a spectroscopic determination of the synthesis yield (A_{280} = 0.07, V = 0.5 mL, 111 nmol, 22 %).

UPLC-MS: t_R = 1.49 min (3-30 % B in 6 min); m/z = 763.1 ($C_{68}H_{109}N_{21}O_{19}$ $(M+2H)^{2+}$, calcd.: 762.9), 509.1 ($C_{68}H_{109}N_{21}O_{19}$ $(M+3H)^{3+}$, calcd.: 508.9); $C_{68}H_{109}N_{21}O_{19}$ (MW = 1523.8 g· mol^{-1}).

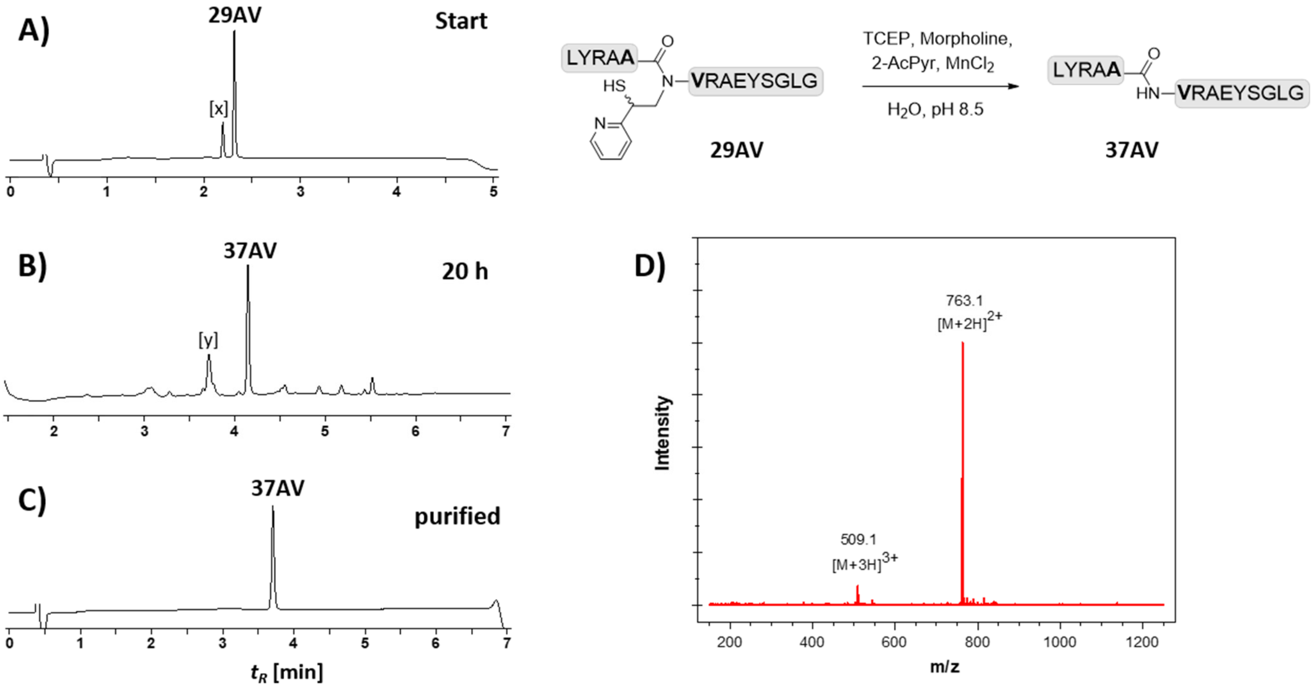

Figure S40: UPLC analysis of auxiliary removal. A) ligation product **29AV**, B) crude material after 20 h reaction and C) purified peptide **37AV**. D) ESI-MS of purified **37AV**. UPLC analysis: A) 3 - 30 % ACN in 4 min, λ = 210 nm; B) 3 % ACN for 1 min, then 3 - 30 % in 6 min, λ = 210 nm; C) 3 - 30 % ACN in 6 min, λ = 210 nm. [x] thioester form by N→S rearrangement**;** [y] nonpeptidic material (m/z = 475).

Removal of Auxiliary from Ligation Product LN (37LN)

To 190 nmol of ligated peptide **29LN,** 190 µL auxiliary cleavage mixture was added (see 10.1). The mixture was shaken for 24 h and the crude ligation product was purified by preparative HPLC using a linear gradient from 3-30 % B in 40 min. The desired auxiliary removal product **37LN** was isolated as a white solid after lyophilization (see figure S41), which was dissolved in H_2O/ACN/TFA (1:1:0.001 v/v/v) for a spectroscopic determination of the synthesis yield (A_{280} = 0.04, V = 0.5 mL, 74 nmol, 39 %).

UPLC-MS: t_R = 2.96 min (3-30 % B in 4 min); m/z = 791.7 ($C_{70}H_{112}N_{22}O_{20}$ $(M+2H)^{2+}$, calcd.: 791.4), 528.1 ($C_{70}H_{112}N_{22}O_{20}$ $(M+3H)^{3+}$, calcd.: 527.9); $C_{70}H_{112}N_{22}O_{20}$ (MW = 1580.8 g· mol^{-1}).

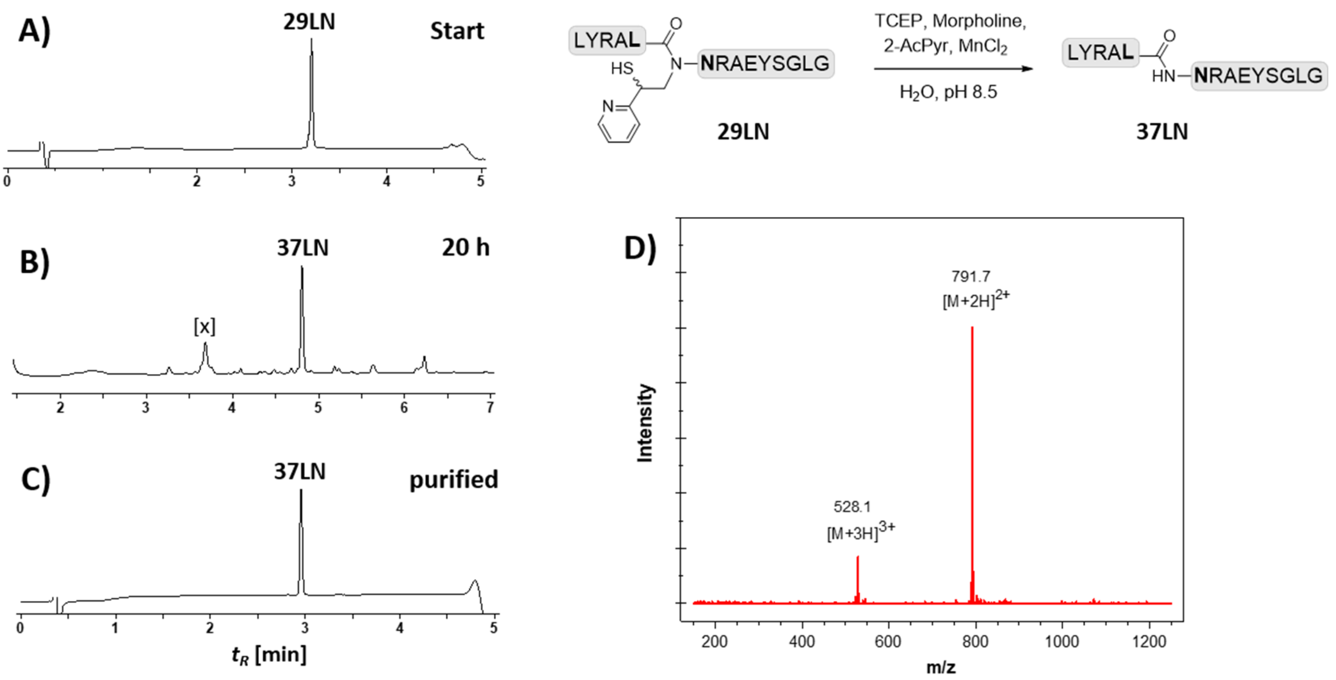

Figure S41: UPLC analysis of auxiliary removal. A) ligation product **29LN**, B) crude material after 20 h reaction and C) purified peptide **37LN**. D) ESI-MS of purified **37LN**. UPLC analysis: A) and C) 3 - 30 % ACN in 4 min, λ = 210 nm; B) 3 % ACN for 1 min, then 3 - 30 % in 6 min, λ = 210 nm. [x] nonpeptidic material (m/z = 475).

Removal of Auxiliary from Ligation Product LR (37LR)

To 190 nmol of ligated peptide **29LR,** 190 µL auxiliary cleavage mixture was added (see 10.1). The mixture was shaken for 24 h and the crude ligation product was purified by preparative HPLC using a linear gradient from 3-30 % B in 40 min. The desired auxiliary removal product **37LR** was isolated as a white solid after lyophilization (see figure S42), which was dissolved in H_2O/ACN/TFA (1:1:0.001 v/v/v) for a spectroscopic determination of the synthesis yield (A_{280} = 0.06, V = 0.5 mL, 109 nmol, 58 %).

UPLC-MS: t_R = 2.90 min (3-30 % B in 4 min); m/z = 812.9 ($C_{72}H_{118}N_{24}O_{19}$ $(M+2H)^{2+}$, calcd.: 812.4), 542.3 ($C_{72}H_{118}N_{24}O_{19}$ $(M+3H)^{3+}$, calcd.: 542.0); $C_{72}H_{118}N_{24}O_{19}$ (MW = 1622.9 g· mol^{-1}).

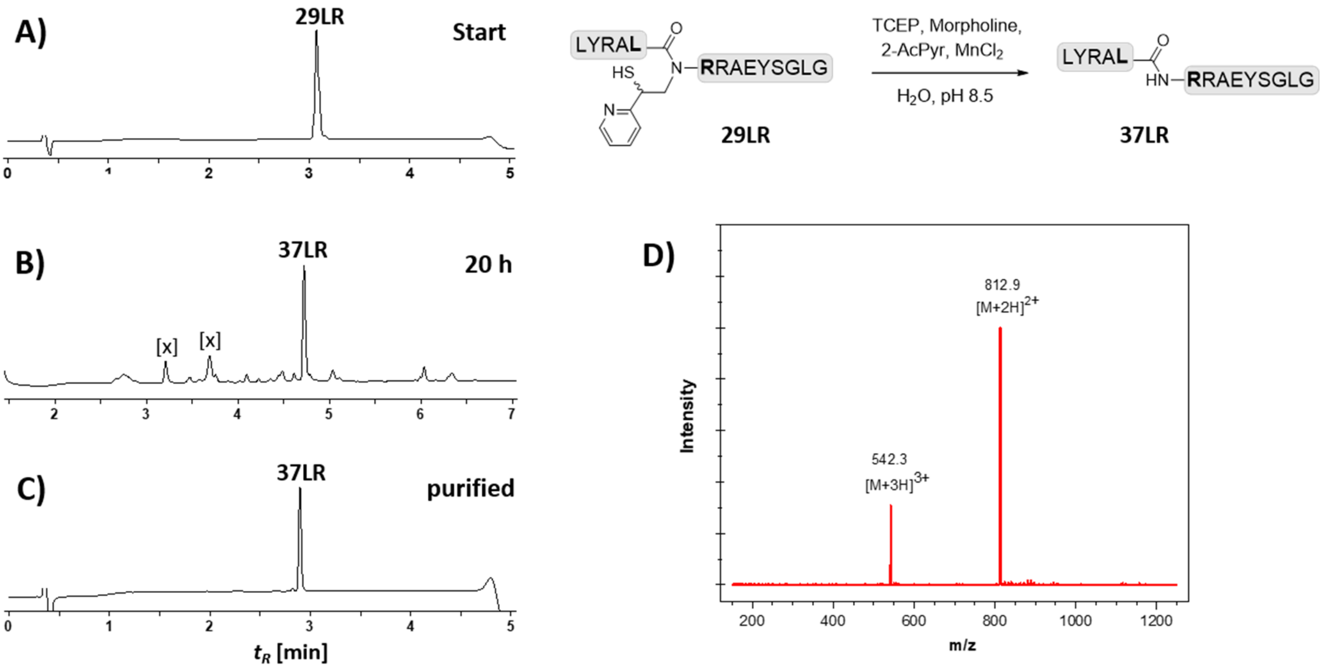

Figure S42: UPLC analysis of auxiliary removal. A) ligation product **29LR**, B) crude material after 20 h reaction and C) purified peptide **37LR**. D) ESI-MS of purified **37LR**. UPLC analysis: A) and C) 3 - 30 % ACN in 4 min, λ = 210 nm; B) 3 % ACN for 1 min, then 3 - 30 % in 6 min, λ = 210 nm. [x] nonpeptidic material.

Removal of Auxiliary from Ligation Product AL (37AL)

To 420 nmol of ligated peptide **29AL,** 420 µL auxiliary cleavage mixture was added (see 10.1). The mixture was shaken for 24 h and the crude ligation product was purified by preparative HPLC rinsing initially for 10 minutes at 15 % B and subsequently using a linear gradient from 15-30 % B in 30 min. The desired auxiliary removal product **37AL** was isolated as a white solid after lyophilization (see figure S43), which was dissolved in H_2O/ACN/TFA (1:1:0.001 v/v/v) for a spectroscopic determination of the synthesis yield (A_{280} = 0.07, V = 0.5 mL, 124 nmol, 29 %).

UPLC-MS: t_R = 3.18 min (3-30 % B in 4 min); m/z = 770.1 ($C_{69}H_{111}N_{21}O_{19}$ $(M+2H)^{2+}$, calcd.: 769.9), 513.9 ($C_{69}H_{111}N_{21}O_{19}$ $(M+3H)^{3+}$, calcd.: 513.6); $C_{69}H_{111}N_{21}O_{19}$ (MW = 1537.8 g· mol^{-1}).

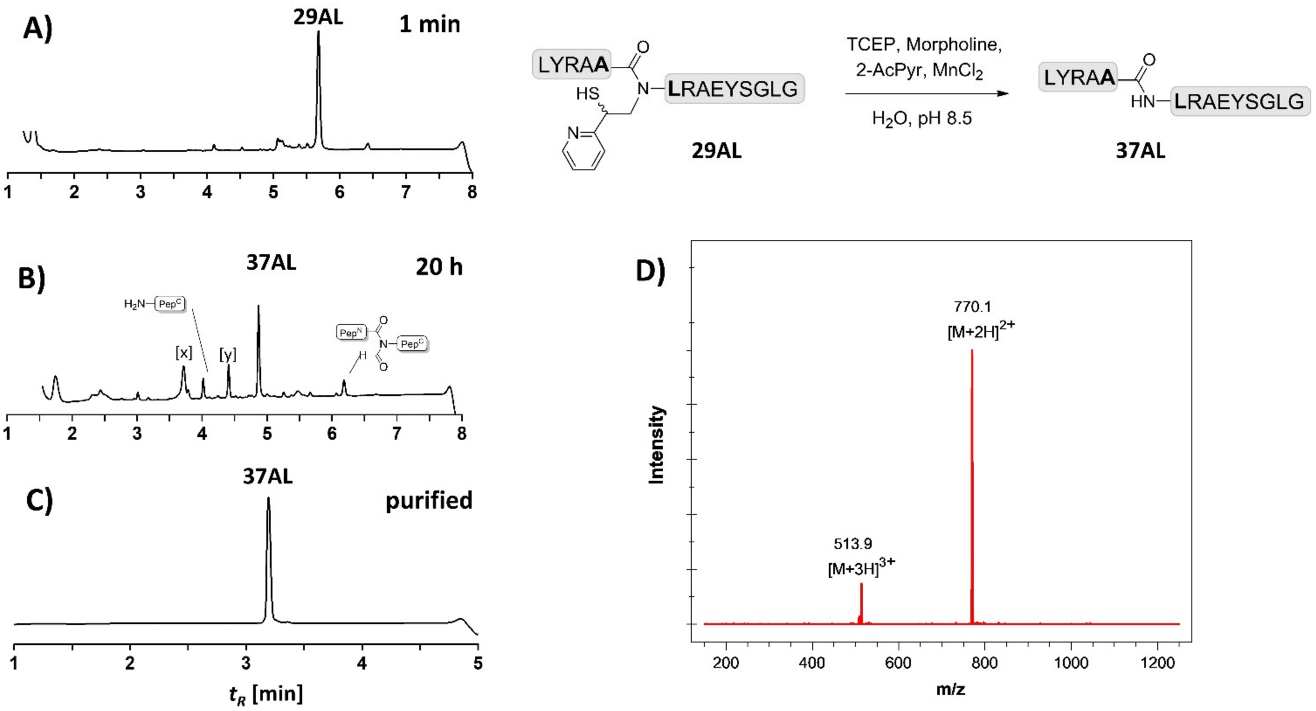

Figure S43: UPLC analysis of auxiliary removal. A) ligation product **29AL**, B) crude material after 20 h reaction and C) purified peptide **37AL**. D) ESI-MS of purified **37AL**. UPLC analysis: A) and B) 3 % ACN for 1 min, then 3 - 30 % in 6 min, λ = 210 nm; C) 3 - 30 % ACN in 4 min, λ = 210 nm. [x] non-peptidic material. [y] m/z = [**37AL**+H$^+$+18]$^+$.

Product Formed by Removal Mixture

Incubation of the removal cocktail over a period of 24 h leads to the formation of a non-peptidic product with a m/z of 475. This not yet identified side product forms regardless of the presence of peptide (see figure S44) and has not been observed to be detrimental for removal reaction. During HPLC purification, overlap with the product peaks of polar peptides (**37AN**, **37AG**, **37AV**, **37AR**) could be avoided by rinsing with 15 % ACN before applying the gradient for purification.

Conditions: 0.5 M TCEP, 2 M morpholine, 5 mM MnCl2, 0.5 M 2-acetylpyridine, pH = 8,5

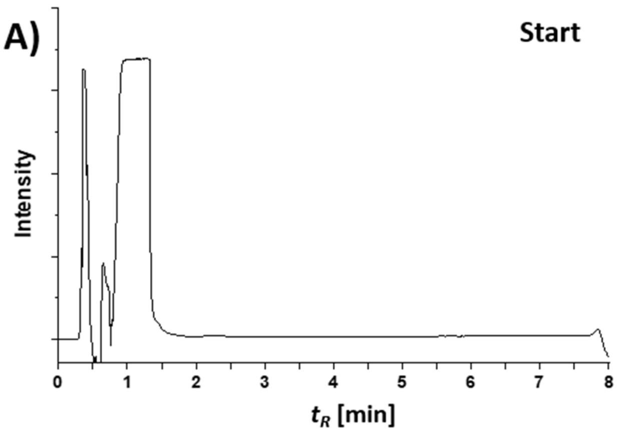

Figure S44: Formation of a non peptidic side product. UPLC trace of the removal mixture without peptide A) at the start and B) after 24 h. UPLC analysis: 3 % ACN for 1 min, then 3 - 30 % in 8 min, λ = 210 nm.

11 Synthesis of P3-P1-P3

P3-P1-P3 (1-54) Thioester (38)

The peptide was synthesized on a pre-loaded chlorotrityl tentagel resin via automated Fmoc-synthesis (see 4.1, loading 4.0 µmol). After cleavage from the resin with HFIP:DCM (7:3 v/v) the thioester was formed according to the general procedure (see 3.1). After 1 h of global deprotection with TFA:TIS:H_2O (96:2:2 v/v/v) and peptide precipitation (see 3.1), the crude peptide was dissolved in H_2O:ACN:DMSO:TFA (3:1:1:0.001 v/v/v/v) and purified by preparative HPLC using a linear gradient from 3-60 % B in 60 min. The desired thioester peptide **38** was isolated as a white solid after lyophilization (see figure S45), which was dissolved in H_2O/ACN/TFA (1:1:0.001 v/v/v) for a spectroscopic determination of the synthesis yield (A_{280} = 0.29, V = 0.5 mL, 0.97 µmol, 24 %).

Peptide sequence: S P E D E I Q Q L E E E I A Q L E Q K N A A L K E K N Q A L K Y G S P E D E I Q A L E E E N A Q L E Q E N A

UPLC-MS: t_R = 4.76 min (3-60 % B in 8 min); m/z 1249.6 ($C_{266}H_{420}N_{74}O_{101}S$ $(M+5H)^{5+}$, calcd.: 1249.8), m/z 1042.1 ($C_{266}H_{420}N_{74}O_{101}S$ $(M+6H)^{6+}$, calcd.: 1041.8), m/z 893.3 ($C_{266}H_{420}N_{74}O_{101}S$ (M+7H)7^{+}, calcd.: 893.1), m/z 781.8 ($C_{266}H_{420}N_{74}O_{101}S$ $(M+8H)^{8+}$, calcd.: 781.6), m/z 695.3 ($C_{266}H_{420}N_{74}O_{101}S$ $(M+9H)^{9+}$, calcd.: 694.9); $C_{157}H_{244}N_{46}O_{52}Se$ (MW = 6246.7 g·mol^{-1}).

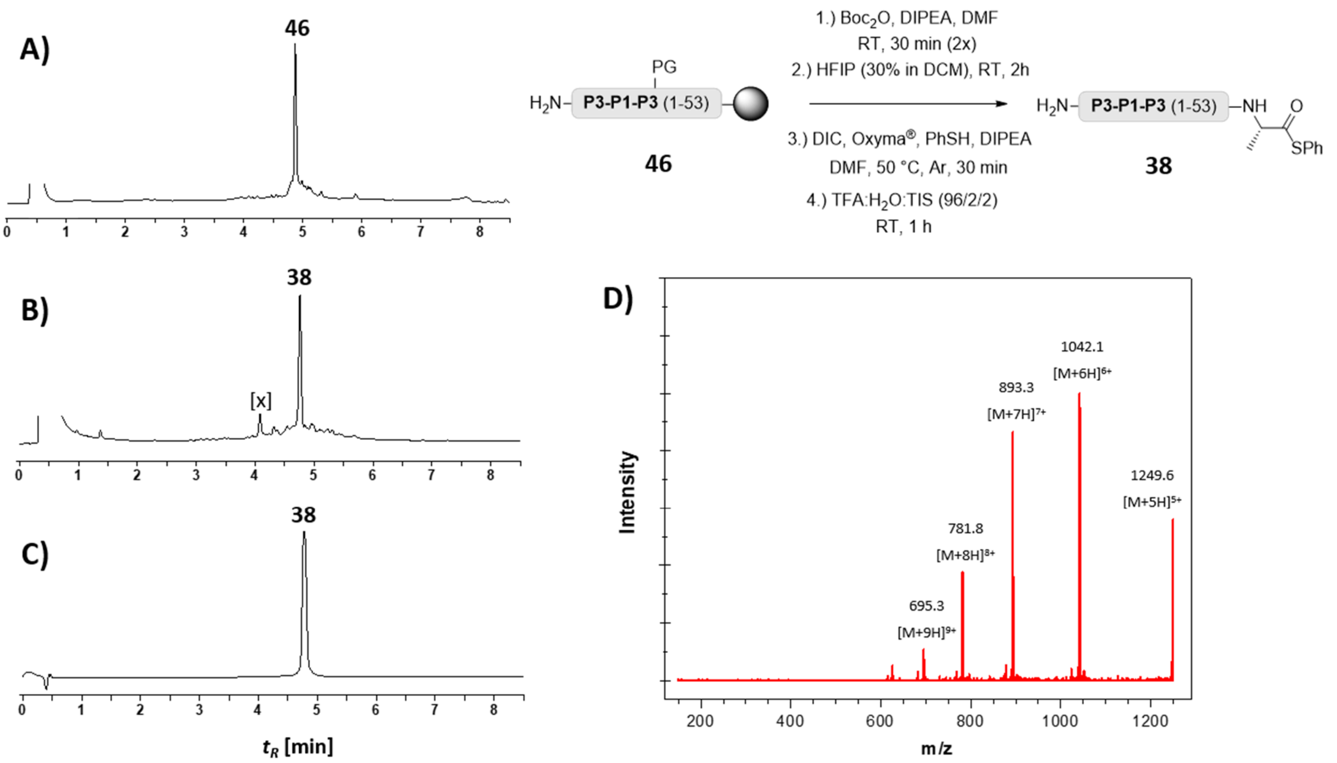

Figure S45: UPLC trace of A) crude **46** after a test cleavage, B) crude **38** and C) purified **38**. D) ESI-MS analysis of purified **38**. UPLC analysis: 20 - 45 % ACN in 8 min, λ = 210 nm. [x] nonpeptidic material.

P3-P1-P3 (55-99) Auxiliary Peptide (39)

The peptide was synthesized on a pre-loaded chlorotrityl tentagel resin via automated Fmoc-synthesis (see 4.1, loading 10.0 µmol). After removal of the N-terminal Fmoc-group the auxiliary was introduced by reductive alkylation with **25** for 24 h (see general procedure, 6.1). Following 30 h of cleavage from the resin with TFA:TIS:H_2O (96:2:2 v/v/v) and peptide precipitation, the crude peptide was dissolved in H_2O:ACN:DMF:TFA (3:1:1:0.001 v/v/v/v) and purified by preparative HPLC using a linear gradient from 3-60 % B in 60 min. The desired auxiliary peptide **39** was isolated as a white solid after lyophilization (see figure S46), which was dissolved in H_2O/ACN/TFA (1:1:0.001 v/v/v) for a spectroscopic determination of the synthesis yield (A_{280} = 1.16, V = 1.0 mL, 2.46 µmol, 25 %).

Peptide sequence: A L E E E I A Q L E Y G S P E D E I Q Q L E E E I A Q L E Q K N A A L K E K N Q A L K Y G

UPLC-MS: t_R = 2.09 min (3-60 % B in 8 min); m/z = 1054.7, ($C_{230}H_{364}N_{58}O_{81}S$ $(M+5H)^{5+}$, calcd.: 1055.0), 879.6 ($C_{230}H_{364}N_{58}O_{81}S$ $(M+6H)^{6+}$, calcd.: 979.3), 753.95 ($C_{230}H_{364}N_{58}O_{81}S$ $(M+7H)^{7+}$, calcd.: 753.8), 659.5 ($C_{230}H_{364}N_{58}O_{81}S$ $(M+8H)^{8+}$, calcd.: 659.7), 586.6 ($C_{230}H_{364}N_{58}O_{81}S$ (M+9H)9^{+}, calcd.: 586.5); $C_{176}H_{272}N_{56}O_{56}S$ (MW = 5269.8 g· mol^{-1}).

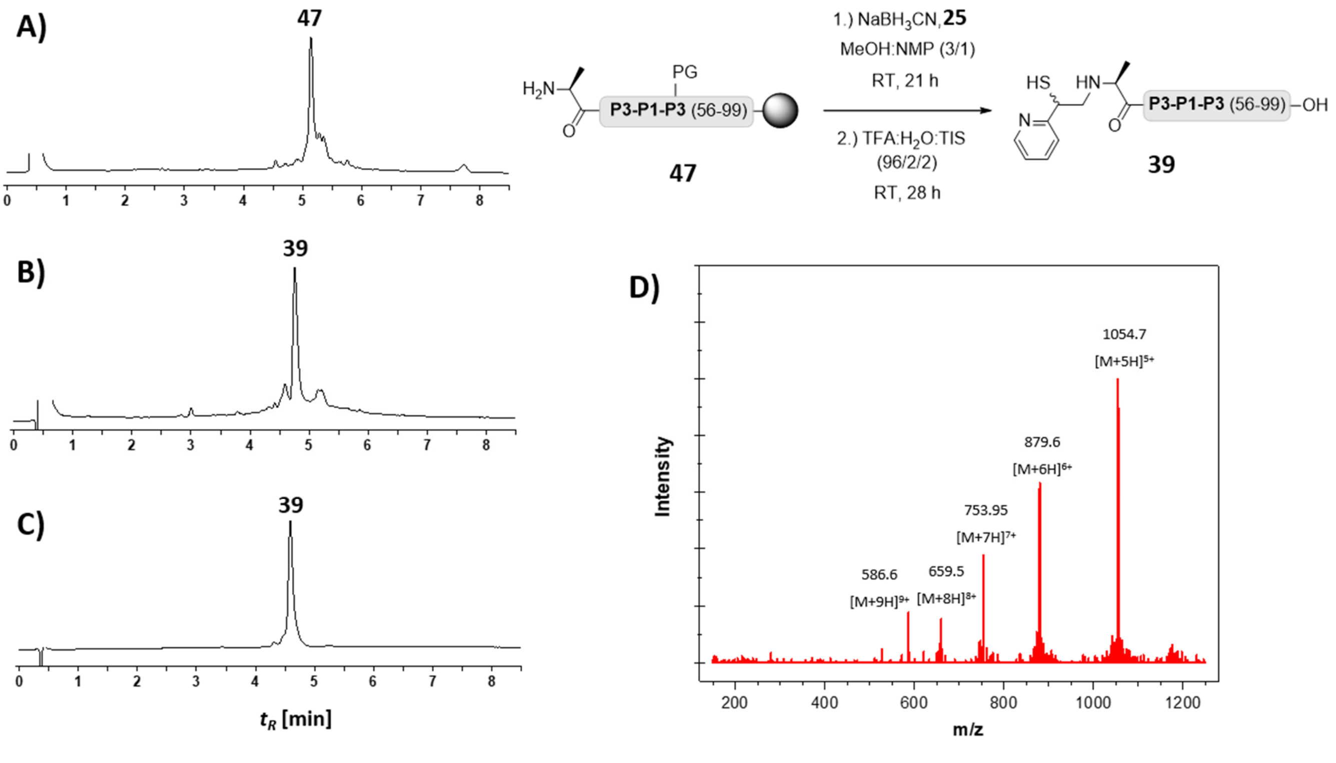

Figure S46: UPLC analysis of crude P3-P1-P3 (55-99) A) before and B) after reductive alkylation. Purified MPyE-peptide **39** analyzed by C) UPLC and D) ESI-MS. UPLC analysis: A) 3 - 60 % ACN in 8 min, λ = 210 nm; B) 20 - 45 % ACN in 8 min, λ = 210 nm; C) 3 - 60 % ACN in 4 min, λ = 210 nm.

Ligation of 38 and 39 (40)

Peptide thioester (550 nmol, 1.4 eq.) **38** and auxiliary peptide (400 nmol, 1 eq.) **39** were combined from stock solutions and lyophilized. The lyophilized peptides were dissolved in 110 µL of ligation buffer (200 mM sodium hydrogen phosphate, 6 M Gdn HCl, 20 mM TCEP, 3 vol.% thiophenol, pH = 7.5). After 24 h the ligation was quenched by addition of 5 µl of a hydrazine solution (81 % in water). Subsequently the ligation solution was purified by preparative HPLC using a linear gradient from 3-60 % B in 60 min. The desired auxiliary containing ligation product **40** was isolated as a white solid after lyophilization (see figure S47), which was dissolved in H_2O/ACN/TFA (1:1:0.001 v/v/v) for a spectroscopic determination of the synthesis yield (A_{280} = 0.23, V = 0.5 mL, 184 nmol, 45 %).

UPLC-MS: t_R = 3.21 min (3-60 % B in 4 min); m/z = 1141.6 ($C_{490}H_{778}N_{128}O_{182}S$ $(M+10H)^{10+}$, calcd.: 1141.6), 1037.9 ($C_{490}H_{778}N_{128}O_{182}S$ $(M+11H)^{11+}$, calcd.:1037.9), 951.6 ($C_{490}H_{778}N_{128}O_{182}S$ $(M+12H)^{12+}$, calcd.: 951.5), 878.6 ($C_{490}H_{778}N_{128}O_{182}S$ $(M+13H)^{13+}$, calcd.: 878.4), 816.1 ($C_{490}H_{778}N_{128}O_{182}S$ $(M+14H)^{14+}$, calcd.: 815.7), 761.5 ($C_{490}H_{778}N_{128}O_{182}S$ $(M+15H)^{15+}$, calcd.: 761.4), $C_{327}H_{510}N_{102}O_{108}S$ (MW = 11406.4 g· mol^{-1}).

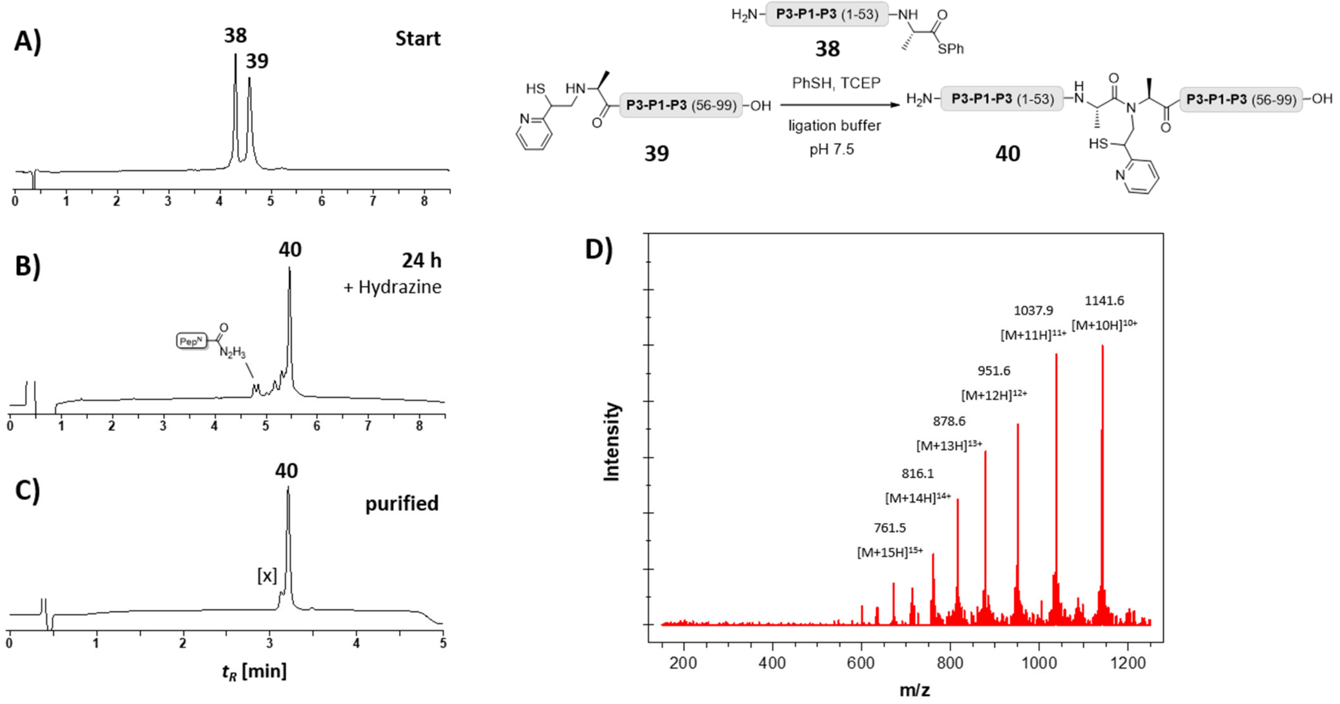

Figure S47: UPLC trace of A) lyophilized starting materials **38** and **39** and B) at t = 24 h of ligation and treatment with hydrazine. Purified ligation product **40** analyzed by C) UPLC-trace and D) ESI-MS. UPLC analysis: A) and B) 20 - 45 % ACN in 8 min, λ = 210 nm; C) 3 - 60 % ACN in 4 min, λ = 210 nm. Pep^N = N-terminal fragment; Pep^C = C-terminal fragment. [x] thioester form by N→S rearrangement.

P3-P1-P3 (41)

274 nmol of the ligation product **40** were dissolved in 274 µL (1 mM) of the auxiliary removal mixture (0.5 M TCEP, 2 M morpholine, 5 mM $MnCl_2$, 0.5 M 2-acetylpyridine, pH = 8.5). After 6 h, the removal product was purified by preparative HPLC using a linear gradient from 3-60 % B in 50 min. The desired native peptide **41** was isolated as a white solid after lyophilization (see figure S48), which was dissolved in H_2O/ACN/TFA (1:1:0.001 v/v/v) for a spectroscopic determination of the synthesis yield (A_{280} = 0.06, V = 0.5 mL, 65 nmol, 24 %).

UPLC-MS: t_R = 3.28 min (3-60 % B in 4 min).

HRMS: m/z = 1610.85 ($C_{483}H_{771}N_{127}O_{182}$ $(M+7H)^{7+}$, calcd.: 1610.65), 1409.61 ($C_{483}H_{771}N_{127}O_{182}$ $(M+8H)^{8+}$, calcd.: 1409.45), 1253.13 ($C_{483}H_{771}N_{127}O_{182}$ $(M+9H)^{9+}$, calcd.: 1252.95), 1127.94 ($C_{483}H_{771}N_{127}O_{182}$ $(M+10H)^{10+}$, calcd.: 1127.76), 1025.48 ($C_{483}H_{771}N_{127}O_{182}$ $(M+11H)^{11+}$, calcd.: 1025.33), 940.12 ($C_{483}H_{771}N_{127}O_{182}$ $(M+12H)^{12+}$, calcd.: 939.97), 867.91 ($C_{483}H_{771}N_{127}O_{182}$ $(M+13H)^{13+}$, calcd.: 867.74), $C_{483}H_{771}N_{127}O_{182}$ (MW = 11269.08 g·mol^{-1}).

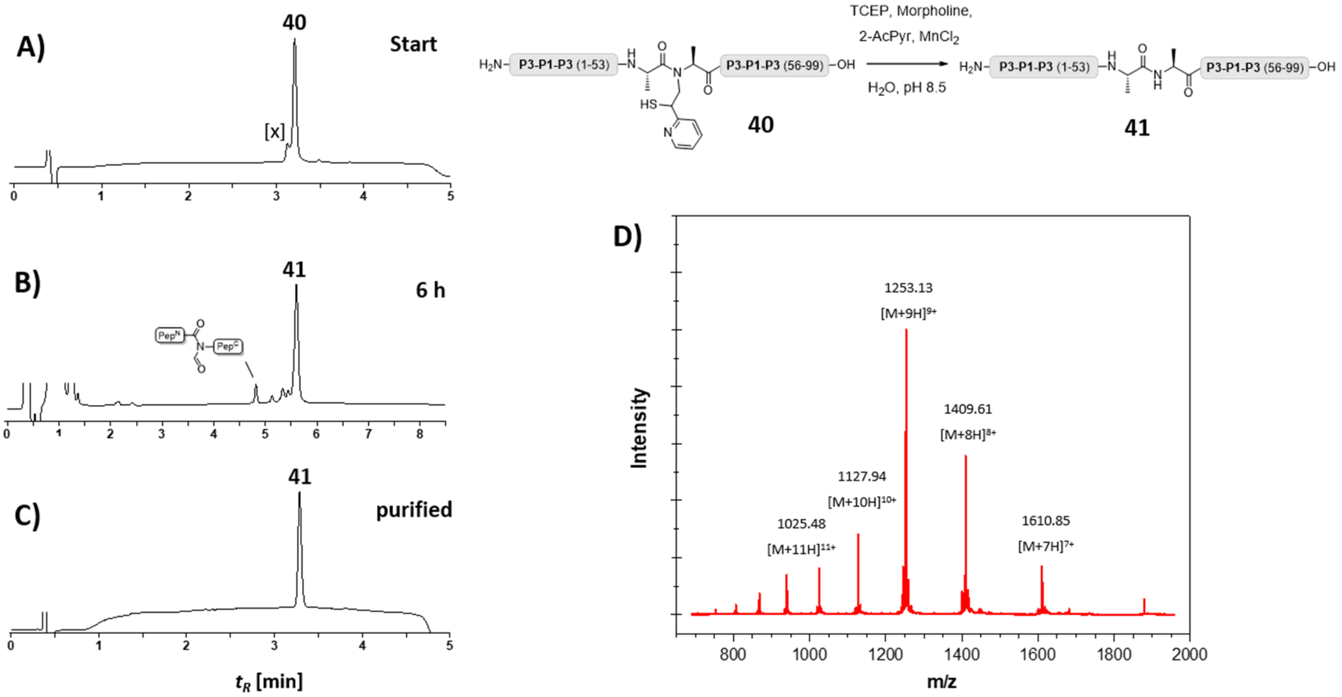

Figure S48: UPLC trace of A) purified starting material **40**, B) crude **41** obtained after 6 h incubation with the removal mixture. Purified **41** analyzed by C) UPLC and D) ESI-HRMS. UPLC analysis: A) 3 - 60 % ACN in 4 min, λ = 210 nm; B) 3 - 60 % ACN in 8 min, λ = 210 nm; C) 3 - 60 % ACN in 4 min, λ = 210 nm. [x] thioester formed by N→S rearrangement.

12 Synthesis of MUC1 80mer

MUC1 (1-38) Selenoester (42)

The peptide was synthesized on a pre-loaded chlorotrityl tentagel resin via automated Fmoc-synthesis (see 4.1, loading 10.8 µmol). After cleavage from the resin with HFIP:DCM (7:3 v/v), the selenoester was formed according to the general procedure (see 4.1). After 1 h of global deprotection with TFA:TIS:H_2O (96:2:2 v/v/v) and peptide precipitation, the crude peptide was purified by preparative HPLC using a linear gradient from 3-20 % B in 50 min. The desired selenoester peptide **42** (10.7 mg, 2.58 µmol, 22 % (based on 3686.9 as 4 x TFA-salt)) was isolated as a white solid after lyophilization (see figure S49).

Peptide sequence: G V T S A P D T R P A P G S T A P P A H G V T S A P D T R P A P G S T A P P

UPLC-MS: t_R = 5,06 min (3-30 % B in 6 min); m/z = 1230.5, ($C_{157}H_{244}N_{46}O_{52}Se$ $(M+3H)^{3+}$, calcd.: 1230.0),922.4 ($C_{157}H_{244}N_{46}O_{52}Se$ $(M+4H)^{4+}$, calcd.: 922.7), 738.0 ($C_{157}H_{244}N_{46}O_{52}Se$ $(M+5H)^{5+}$, calcd.: 738.4); $C_{157}H_{244}N_{46}O_{52}Se$ (MW = 3686.9 g·mol^{-1}).

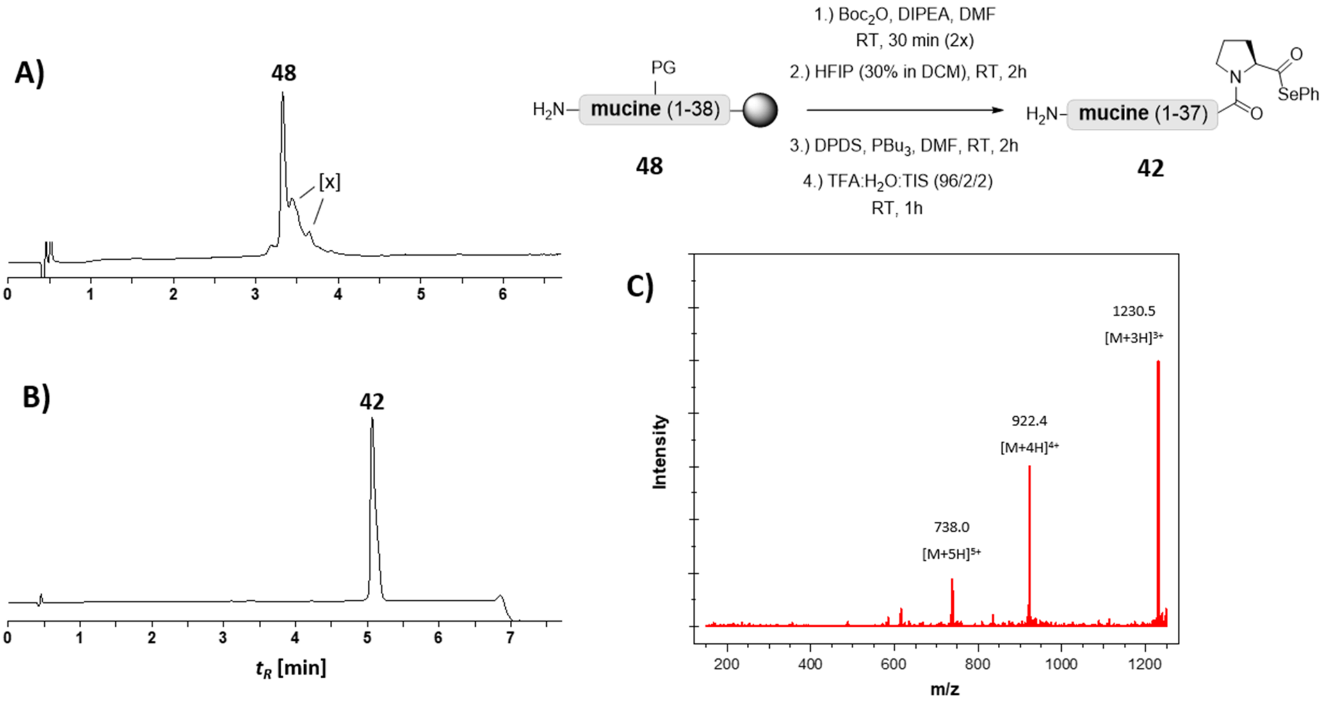

Figure S49: UPLC trace of A) crude **48** after a test cleavage and B) purified **42**. C) ESI-MS analysis of purified **42**. UPLC analysis C) and ESI-MS spectrum D) after HPLC purification. UPLC analysis: 3 - 30 % ACN in 6 min, λ = 210 nm. [x] truncations from SPPS.

MUC1 (39-80) MPyE-Peptide (43)

The peptide was synthesized on a rink-amide tentagel resin via automated Fmoc-synthesis (see 4.1, loading 11.7 µmol). After removal of the N-terminal Fmoc-group the auxiliary was introduced by reductive alkylation of **49** for 24 h (see general procedure, 6.1). Following 30 h of cleavage from the resin with TFA:TIS:H_2O (96:2:2 v/v/v) and peptide precipitation, the crude peptide was purified by preparative HPLC using a linear gradient from 3-20 % B in 50 min. The desired auxiliary peptide **43** (5.87 mg, 1.2 µmol, 11 % (based on 4100.51 as 7 x TFA-salt)) was isolated as a white solid after lyophilization (see figure S50).

Peptide sequence: A H G V T S A P D T R P A P G S T A P P A H G V T S A P D T R P A P G S T A P P A H

UPLC-MS: t_R = 3.26 min (3-30 % B in 6 min); m/z = 1026.1, ($C_{176}H_{272}N_{56}O_{56}S$ $(M+4H)^{4+}$, calcd.: 1026.1), 821.3 ($C_{176}H_{272}N_{56}O_{56}S$ $(M+5H)^{5+}$, calcd.: 821.1), 684.3 ($C_{176}H_{272}N_{56}O_{56}S$ $(M+6H)^{6+}$, calcd.: 684.4), 586,9 ($C_{176}H_{272}N_{56}O_{56}S$ $(M+7H)^{7+}$, calcd.: 586.8); $C_{176}H_{272}N_{56}O_{56}S$ (MW = 4100.51 g·mol^{-1}).

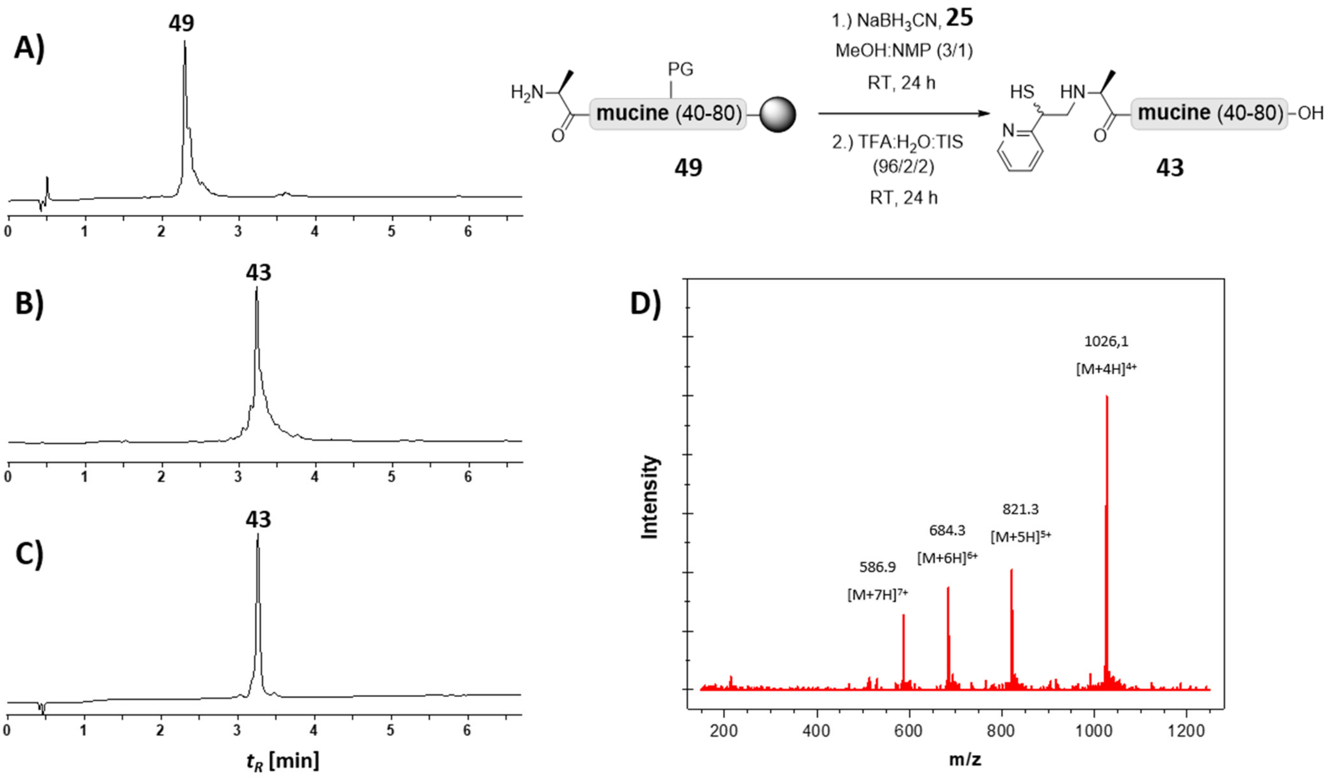

Figure S50: UPLC analysis of crude MUC1 (39-80) A) before and B) after reductive alkylation. Purified MPyE-peptide **43** analyzed by C) UPLC and D) ESI-MS. UPLC analysis: 3 - 30 % ACN in 6 min, λ = 210 nm.

Ligation of 42 and 43 (44)

3.5 µmol peptide selenoester (2 eq.) **42** and 1.8 µmol auxiliary peptide (1 eq.) **43** were weighed in and dissolved in 354 µL of ligation buffer (200 mM phosphate, 6 M Gdn HCl, 100 mM TCEP, 28 mM DPDS, pH = 6.2). After 24 h the ligation was quenched by addition of 40 µl of a hydrazine solution (81 % in water) and 40 µl of a 1 M TCEP solution. Subsequently the ligation solution was purified by preparative HPLC using a linear gradient from 3-20 % B in 50 min. The desired auxiliary containing ligation product **44** (8.72 mg, 880 nmol, 50 % (based on 8770.42 as 10 x TFA-salt)) was isolated as a white solid after lyophilization (see figure S51).

UPLC-MS: t_R = 3.66 min (3-30 % B in 6 min); m/z = 1091.3, ($C_{327}H_{510}N_{102}O_{108}S$ $(M+7H)^{7+}$, calcd.: 1091.0),954.6 ($C_{327}H_{510}N_{102}O_{108}S$ $(M+8H)^{8+}$, calcd.: 954.8), 849.6 ($C_{327}H_{510}N_{102}O_{108}S$ $(M+9H)^{9+}$, calcd.: 848.8), 764.1 ($C_{327}H_{510}N_{102}O_{108}S$ $(M+10H)^{10+}$, calcd.: 764.0), 694.6 ($C_{327}H_{510}N_{102}O_{108}S$ $(M+11H)^{11+}$, calcd.: 694.7), $C_{327}H_{510}N_{102}O_{108}S$ (MW = 7630.34 g·mol^{-1}).

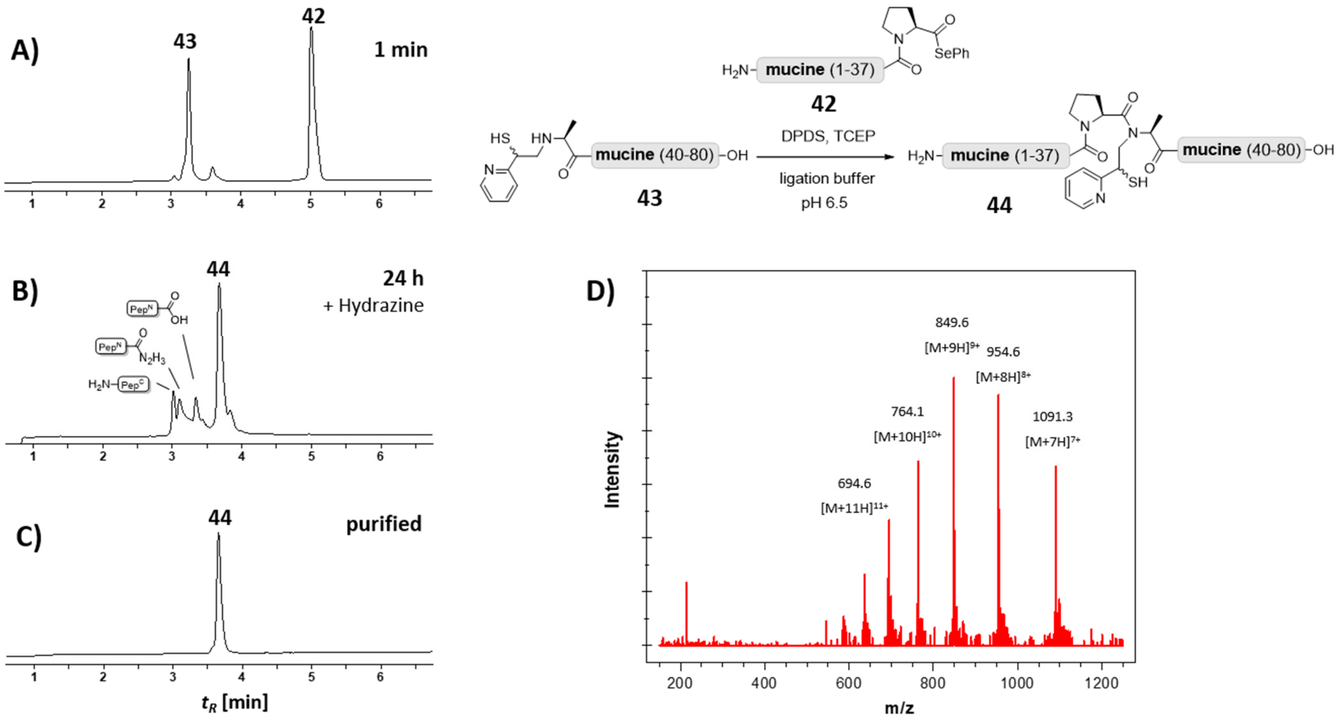

Figure S51: UPLC analysis of ligation A) at t = 1 min and B) after treatment with hydrazine. Purified ligation product **44** analyzed by C) UPLC-trace and D) ESI-MS. UPLC analysis: 3 - 30 % ACN in 6 min, λ = 210 nm. Pep^N = N-terminal fragment; Pep^C = C-terminal fragment.

MUC1 (45)

145 nmol of the ligation product were dissolved in 145 µL (1 mM) of the auxiliary removal mixture (0.5 M TCEP, 2 M morpholine, 5 mM $MnCl_2$, 0.5 M 2-acetylpyridine, pH = 8.5). After 5 h the removal product was purified by preparative HPLC using a linear gradient from 3-20 % B in 50 min. The desired native peptide **45** (0,38 mg, 45 nmol, 32 % (based on 7493.1 as 9 x TFA-salt)) was isolated as a white solid after lyophilization (see figure S52).

UPLC-MS: t_R = 5.40 min (3-60 % B in 8 min); m/z = 1249.5, ($C_{320}H_{503}N_{101}O_{108}$ $(M+6H)^{6+}$, calcd.: 1249.9), 1071.7, ($C_{320}H_{503}N_{101}O_{108}$ $(M+7H)^{7+}$, calcd.: 1071.4), 937.6 ($C_{320}H_{503}N_{101}O_{108}$ $(M+8H)^{8+}$, calcd.: 937.6), 833.6 ($C_{320}H_{503}N_{101}O_{108}$ $(M+9H)^{9+}$, calcd.: 833.6), 750.2 ($C_{320}H_{503}N_{101}O_{108}$ $(M+10H)^{10+}$, calcd.: 750.3), 682.3 ($C_{320}H_{503}N_{101}O_{108}$ $(M+11H)^{11+}$, calcd.: 682.2), $C_{320}H_{503}N_{101}O_{108}$ (MW = 7493.14 g·mol^{-1}).

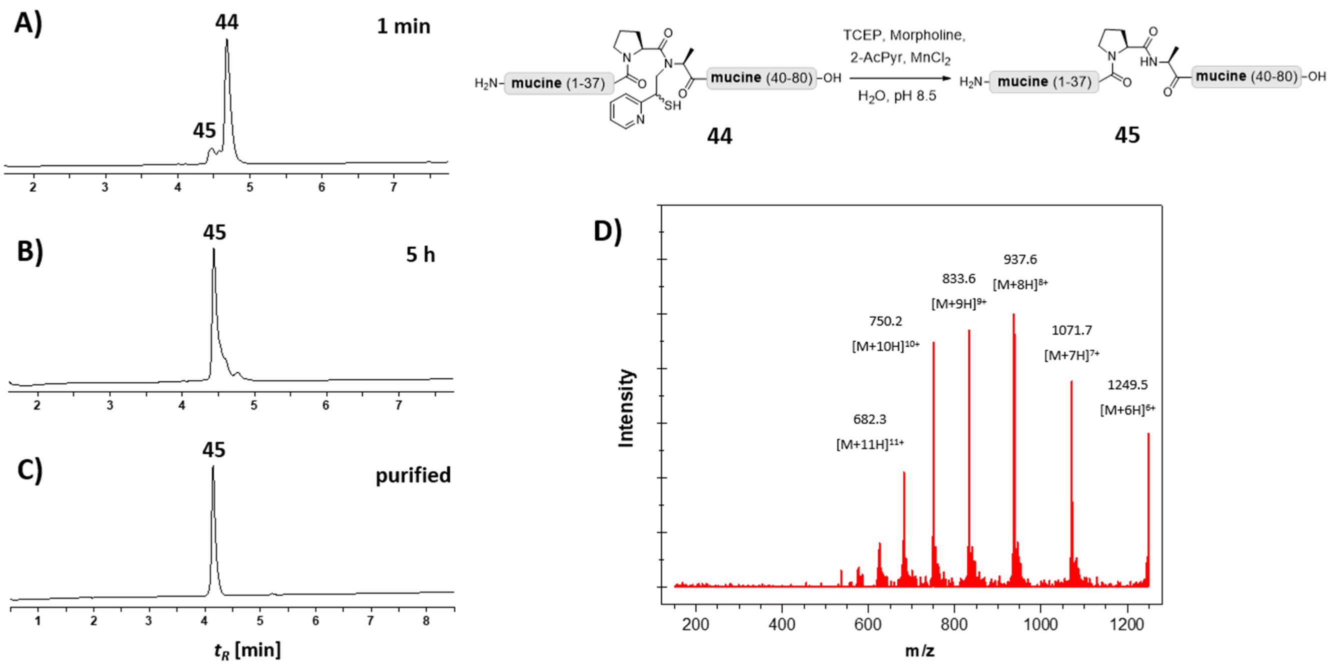

Figure S52: UPLC trace after A) t = 5 min and B) t = 5 h of incubation with the removal mixture. Purified **45** analyzed by C) UPLC and D) ESI-HRMS. UPLC analysis: A) and B) 3 % ACN for 1 min, then 3 - 30 % in 6 min, λ = 210 nm. C) 3 - 30 % ACN in 8 min, λ = 210 nm.

13 Inductively coupled plasma atomic emission spectroscopy

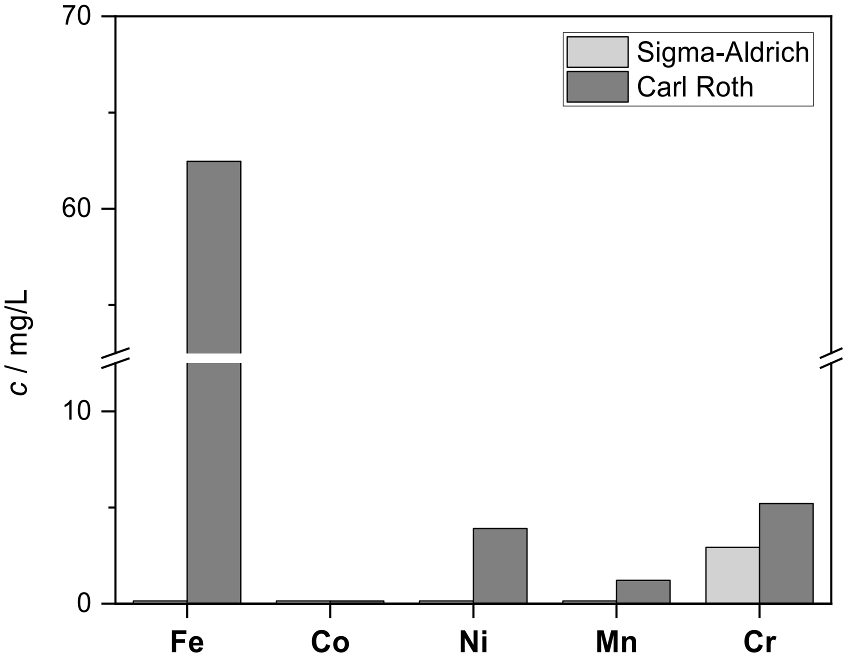

Figure S53: Amount of metal impurities found in two TCEP samples.

Tris(2-carboxyethyl)phosphine hydrochloride from *Carl-Roth* (89 mg, Product Nr. HN95.02 Charge 399287904.) and *Sigma-Aldrich* (112 mg, Product Nr. C406-10G Charge Nr. 10030123801003012380) were dissolved in a 5 mL of H_2O and 5 mL HNO_3 (65 %, *suprapur*, *Merck*). The obtained solutions were diluted to 50 mL with H_2O. From this solution, a 100 µL sample was mixed with 65 µL HNO_3 (65 %, *suprapur*, *Merck*) and further diluted to 13 mL with H_2O prior to the analysis. The amounts found are listed in Figure S53.

To assess the effect of the metal impurities on the auxiliary removal, test reactions were performed at the AN junction. 100 nmol of **29AN** was dissolved in a mixture of 0.5 M TCEP (*Sigma-Aldrich*), 2 M morpholine, 0.1 M 2-acetylpyridine and either $FeCl_2$, $FeCl_3$, $MnCl_2$ or $NiCl_2$ in 0.2 mM concentration. To monitor the progress of the reaction, aliquots were withdrawn from the ligation mixture, quenched with an aqueous solution of 0.1 % TFA and analyzed by UPLC-MS. Of these test reactions, only $MnCl_2$ was found to be beneficial for the removal reaction.

14 Scope and Limitations of the MPyE Auxiliary

14.1 Ligation at difficult ligation sites

Reaction at LL and LV ligation sites proceeded slowly. For the LV junction, approximately half of the starting material was converted to the corresponding ligation product after 24 h and a large portion of thioester **28L** was hydrolyzed (see figure S54). For these ligation sites, we therefor recommend using the peptide thioester in larger excess. Alternatively, the selenoester method may be applied.

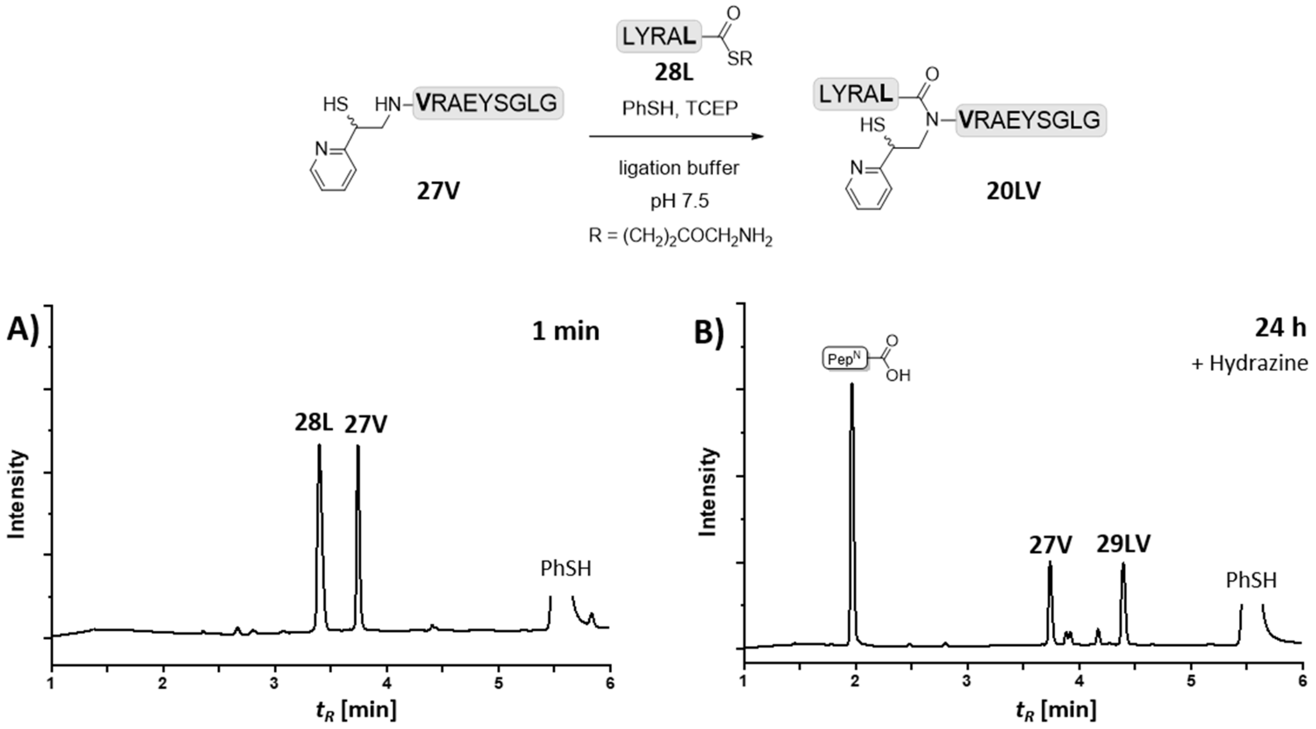

Figure S54: Ligation at the LV junction. UPLC analysis of aliquots withdrawn at A) t = 1 min and quenching with 0.1 % TFA in H_2O and B) t = 24 h after quenching with hydrazine (2.5 %). UPLC analysis: 3 - 30 % ACN in 6 min, λ = 210 nm. Pep^N = N-terminal fragment.

Decomposition of MPyE-Peptide in Presence of Oxidizing Agents

We noticed a rapid decomposition of MPyE-peptides, when brought into contact with peroxide impurities (from Et_2O) or DMSO. We therefore recommend avoiding the use of any oxidizing agents.

15 Quantum Chemical Calculation

All DFT calculations were performed using a locally modified version of the ADF engine[4] (ADF 2020, SCM, Theoretical Chemistry, Vrije Universiteit, Amsterdam, The Netherlands, htpp://www.scm.com) in the AMS program suite based on the 2020 release. (AMS 2020, SCM, Theoretical Chemistry, Vrije Universiteit, Amsterdam, The Netherlands, htpp://www.scm.com). Throughout this publication, the PBE0 functional[5] and the TZ2P basis sets[6] as well as the D4 dispersion correction[7] were used. Numerical quality was set to “good” while the “verygood” option was chosen for the density fitting (ZLMFit). The SCF was converged to a DIIS error of 10^{-7}, structure optimizations were converged to a maximum gradient entry below 10^{-4} Hartree/bohr and an energy change of 10^{-6} Hartree between consecutive structures. Solvent effects were included using the recently published 3D-RISM-SCF implementation[8]. The solvent bulk susceptibility functions were obtained by DRISM using the HNC closure and 16384 grid points with a spacing of 0.01 Å, converged to a residue norm below 10^{-13}. The 3D-RISM equations were solved on a cartesian grid covering a 36 Å box, using 120 grid points along each direction and the KH closure. The Lennard-Jones parameters for the solute atoms were taken from the Amber force field (GAFF)[9]. The obtained free energy of solvation was corrected the PC+ pressure correction[10].For all optimized structures, numerical frequencies were calculated using a displacement of 0.002 Å. All structures showed the appropriate number of real and imaginary frequencies. Intrinsic reaction coordinate (IRC) calculations verified that all transition states connected the corresponding minima. Cartesian coordinates for all optimized structures can be found below.

Additional DLPNO-CCSD(T)-F12 calculations were performed for each structure using the ORCA program, version 4.2.0, and employed the cc-pVTZ-F12 basis sets[11] and the aug-cc-pVQZ/C[12] and cc-pVTZ-F12/CABS[13] auxiliary basis sets. These gas phase energies were used to correct the energies in solution using:

$$E_{corr.} = E_{DFT,sol} + (E_{CCSD(T),gas} - E_{DFT,gas})$$

Cartesian Coordinates

Substrate

C	-2.46572500	-0.57855800	0.77789900
N	-1.59509000	0.21417500	0.14564100
C	-0.86694600	-0.30452000	-0.84192800
C	-0.99889500	-1.63549800	-1.24536900
C	-1.90387200	-2.45123700	-0.58539900
C	-2.65473400	-1.91521800	0.45736100
C	0.08187200	0.62045000	-1.57920500
S	1.76283100	-0.08470600	-1.77464500
C	2.16103200	-0.75014600	-0.18187200
C	3.50428300	-1.42084800	-0.16486900
C	0.10022800	2.06408400	-1.09944900
N	0.74637500	2.26201100	0.18300200
C	0.62337100	3.64411200	0.62141200
O	1.42973600	-0.68865700	0.78547500
H	3.35917900	-2.47833000	0.06872900
H	4.02909000	-1.32778100	-1.11543200
H	4.10452100	-0.97841200	0.63294200
H	-0.24951900	0.64285200	-2.62376100
H	-0.94458700	2.41339300	-1.09358400
H	0.62773600	2.66619300	-1.84586200
H	0.30821800	1.65016400	0.86498700
H	-0.39408000	-2.01657100	-2.06016200
H	-2.02430200	-3.48814400	-0.87918000
H	-3.37177500	-2.51402100	1.00591200
H	-3.04003300	-0.12208000	1.57965300
H	1.06426400	3.76205100	1.61260000
H	-0.41998900	3.99092500	0.66167200
H	1.16256200	4.29793200	-0.06980100

TS 1

C	-2.55611000	-0.81426100	0.81340800
N	-1.67695300	-0.02435600	0.18883600
C	-1.11637400	-0.46609100	-0.93693400
C	-1.43318400	-1.71069000	-1.48800400
C	-2.34856500	-2.52194600	-0.83779200
C	-2.92237700	-2.06887200	0.34794500
C	-0.13723400	0.44228500	-1.64558900
S	1.52400200	-0.30482700	-1.86862000
C	1.99780900	-0.48698400	-0.11074700
C	3.40482500	-0.05540600	0.16282700
C	0.05645400	1.81664200	-1.01161200
N	0.83770900	1.73886400	0.19997500
C	1.51997900	2.96268600	0.55893300
O	1.30076100	-1.09755500	0.67377900
H	3.67906700	-0.29113200	1.19068700
H	4.07585900	-0.57966900	-0.52482500
H	3.50713100	1.01400300	-0.02434800
H	-0.48248800	0.57213200	-2.67616900
H	-0.92787300	2.28205100	-0.86375800
H	0.60325000	2.43596100	-1.72712600
H	0.27195000	1.39029900	0.96593400
H	-0.95657900	-2.02870900	-2.40826800
H	-2.61155300	-3.49167900	-1.24577800
H	-3.63854800	-2.66889300	0.89628200

H	-2.98923400	-0.42371300	1.73042200
H	2.07322700	2.81412800	1.48837000
H	0.84711200	3.82194600	0.69345300
H	2.23936600	3.22170700	-0.22400600

Intermediate 1

C	-2.30176300	-0.99324900	0.92093100
N	-1.21339800	-0.51220200	0.32538600
C	-1.04877500	-0.72139300	-0.98615100
C	-1.97581200	-1.42275400	-1.75029000
C	-3.10697700	-1.93227700	-1.12191200
C	-3.27610000	-1.71638500	0.24029100
C	0.20713900	-0.15618900	-1.59660500
S	1.68803400	-1.09980200	-1.06181800
C	2.23170200	0.16489700	0.31644000
C	3.47396800	0.87786200	-0.19815400
C	0.48458600	1.26574200	-1.12617700
N	1.00314000	1.14616600	0.24901800
C	1.26560100	2.43836600	0.90097100
O	2.32267000	-0.34876300	1.50041800
H	3.82470200	1.59382500	0.54904100
H	4.25055900	0.12694300	-0.34635400
H	3.31564200	1.38858400	-1.14919300
H	0.14678400	-0.18653900	-2.68401200
H	-0.42112600	1.87465800	-1.11068500
H	1.23518300	1.75570200	-1.74699900
H	0.25594700	0.62744000	0.75933400
H	-1.81137400	-1.56272800	-2.81228100
H	-3.84598200	-2.48656600	-1.68977500
H	-4.14202300	-2.09539000	0.76914400
H	-2.40040700	-0.79736900	1.98445800
H	1.72913200	2.25172900	1.86815700
H	0.32222000	2.96419100	1.03950400
H	1.92815500	3.03342200	0.27458700

TS 2

C	-2.77029000	0.11411000	-0.08418300
N	-1.44266700	0.21984600	-0.05542000
C	-0.82113500	1.40666200	-0.15507000
C	-1.55789100	2.57283900	-0.29212300
C	-2.94502000	2.48198500	-0.33621700
C	-3.56379600	1.23934200	-0.23304200
C	0.67820500	1.33714700	-0.10864400
S	1.32616800	0.65473500	-1.68286400
C	1.36501700	-1.19934200	-1.00227500
C	2.84272700	-1.56085000	-0.87923700
C	1.13473600	0.30952300	0.92534100
N	0.73288900	-0.97697900	0.35396400
C	0.91816500	-2.10093900	1.26679100
O	0.66384100	-2.02494300	-1.73573800
H	2.93179700	-2.58974600	-0.51969200
H	3.30525300	-1.49875900	-1.86487500
H	3.38953000	-0.89946500	-0.20504400
H	1.10524000	2.32163700	0.07367200
H	0.62572900	0.45023000	1.88281800
H	2.21430800	0.37017500	1.09347000
H	-0.58050100	-0.62690500	0.11500100
H	-1.05103900	3.52730200	-0.35961200

H	-3.54270700	3.37974300	-0.44558000
H	-4.64093300	1.14083300	-0.26384200
H	-3.18544000	-0.88165900	0.01191500
H	0.68217400	-3.02803300	0.74549600
H	0.24035900	-1.99053700	2.11432600
H	1.94528300	-2.14795600	1.64066100

Intermediate 2

C	-2.90110300	0.21245600	-0.07183100
N	-1.56496000	0.25493000	-0.06696600
C	-0.85584100	1.39468900	-0.16186200
C	-1.53784100	2.59693300	-0.26218100
C	-2.92762200	2.58815600	-0.27617800
C	-3.62196600	1.38380000	-0.18336500
C	0.63287900	1.25499900	-0.14997600
S	1.21861400	0.56041000	-1.74134700
C	1.44290500	-1.26913800	-1.00457100
C	2.95557400	-1.46455500	-0.88874600
C	1.09943000	0.22579400	0.88149000
N	0.78463800	-1.07415300	0.31716500
C	1.05592400	-2.16198900	1.24602200
O	0.84963800	-2.18179900	-1.74200100
H	3.15669100	-2.46576400	-0.49666700
H	3.40870300	-1.38305500	-1.87741800
H	3.43032200	-0.72937300	-0.23625900
H	1.08693100	2.22998400	0.02080800
H	0.56891700	0.34979900	1.83036900
H	2.17142500	0.36142700	1.07955400
H	-0.96020700	-0.60087500	0.03513300
H	-0.97880600	3.52162000	-0.32536200
H	-3.47167500	3.52221500	-0.35560800
H	-4.70290600	1.35075800	-0.19364700
H	-3.35855600	-0.76412300	0.01353500
H	0.87897800	-3.11679800	0.75070800
H	0.38145700	-2.08617200	2.10075100
H	2.08867100	-2.14424200	1.61816400

TS 3

C	-2.87546000	0.19172100	-0.11006100
N	-1.53896300	0.25189800	-0.08444600
C	-0.84210700	1.40176600	-0.14268600
C	-1.54243800	2.59718500	-0.22444300
C	-2.93007900	2.57039100	-0.25852300
C	-3.61045300	1.35426000	-0.20371900
C	0.64778400	1.28972700	-0.13457000
S	1.21305400	0.75436200	-1.79192600
C	1.45507300	-1.41974400	-0.95351700
C	2.96990800	-1.46953300	-0.92838400
C	1.14801800	0.22621200	0.84540600
N	0.79811300	-1.07823600	0.30754100
C	0.97543700	-2.14845600	1.28962200
O	0.84960500	-2.25178000	-1.67316500
H	3.25999300	-2.43113700	-0.48923500
H	3.35672800	-1.42687100	-1.94528400
H	3.42002300	-0.66837700	-0.34471900
H	1.07070100	2.26275500	0.11452400
H	0.66465100	0.33693200	1.82095500
H	2.22721800	0.35439000	0.99840500

H	-0.92858100	-0.59825700	0.00120400
H	-0.99482100	3.53018100	-0.26037800
H	-3.48578000	3.49867500	-0.32398600
H	-4.69061300	1.30728900	-0.22941400
H	-3.32010100	-0.79282300	-0.05386700
H	0.71644500	-3.10437600	0.83519500
H	0.31028200	-1.97279800	2.13646200
H	2.00657600	-2.19942500	1.66272300

Intermediate 3

C	-3.15750900	0.75316800	-0.90814700
N	-1.84466200	0.72451600	-1.18046100
C	-0.89800500	1.24555400	-0.39306100
C	-1.29806400	1.87554200	0.77980100
C	-2.64643500	1.93085600	1.09916800
C	-3.59354400	1.35652500	0.24940700
C	0.54184700	1.11906900	-0.82796200
S	0.72014700	0.45372400	-2.51886100
C	1.96100100	-2.00512100	-0.17565000
C	3.29905900	-1.57437800	-0.71284500
C	1.38089600	0.35283700	0.22193900
N	1.11941700	-1.06537000	0.29517200
C	-0.14300900	-1.49035500	0.86998500
O	1.64624100	-3.20705500	-0.14677800
H	3.88866400	-1.07569900	0.05982200
H	3.83340300	-2.45784400	-1.05417000
H	3.17221500	-0.88022800	-1.54565500
H	0.93875000	2.13895000	-0.80880000
H	1.20314000	0.78972400	1.21087000
H	2.42821100	0.51880300	-0.01624900
H	-1.47742600	0.30120600	-2.05251700
H	-0.55314600	2.32003000	1.42778700
H	-2.96496600	2.42287600	2.01071100
H	-4.65055400	1.38525500	0.47669200
H	-3.81406800	0.29364600	-1.63481800
H	-0.90936100	-1.64287700	0.10389500
H	-0.48878300	-0.72990200	1.57048900
H	-0.00572500	-2.42763800	1.40629800

TS 4

C	-3.12928100	0.76414900	-0.93305300
N	-1.82277400	0.72085200	-1.19281900
C	-0.92568600	1.23638500	-0.36100500
C	-1.32366100	1.85968100	0.81797900
C	-2.67996700	1.91807900	1.10900100
C	-3.60191700	1.35816000	0.22547400
C	0.52832700	1.09791300	-0.79081900
S	0.56505700	0.31230900	-2.44187400
C	1.92706400	-1.98821000	-0.19371100
C	3.23761200	-1.55695400	-0.79631000
C	1.38904700	0.36420500	0.25725700
N	1.13209200	-1.05379000	0.35851100
C	-0.12088000	-1.46948300	0.95986100
O	1.59827800	-3.18760500	-0.18708500
H	3.86666000	-1.05713400	-0.05647300
H	3.75362900	-2.44052900	-1.16468100
H	3.07038700	-0.86565700	-1.62490100
H	0.94025000	2.10846300	-0.86220300

H	1.21674300	0.82021200	1.23794900
H	2.43605300	0.52696200	0.01363800
H	-0.97303200	0.28280700	-2.20170200
H	-0.58986700	2.29122600	1.48834100
H	-3.01986400	2.39851800	2.01944000
H	-4.66527700	1.38789400	0.42710000
H	-3.79205800	0.31919900	-1.66707500
H	-0.91320900	-1.57539700	0.21217800
H	-0.42823600	-0.72086900	1.69026100
H	0.01224500	-2.42557300	1.46278400

Product

C	-3.12634800	0.54888800	-0.66838200
N	-1.84728900	0.51671300	-1.04961200
C	-0.96545200	1.25753100	-0.38115900
C	-1.33909000	2.07149200	0.69066200
C	-2.66985000	2.10261500	1.08161400
C	-3.58816700	1.31897300	0.39071300
C	0.50338800	1.18207300	-0.74519200
S	0.83509500	0.75392200	-2.48232200
C	1.80538300	-2.03462800	-0.25258400
C	3.12797800	-1.62434300	-0.84214600
C	1.31073300	0.32536400	0.25076000
N	0.99063100	-1.08606900	0.24633800
C	-0.23453000	-1.51785200	0.90170400
O	1.47490300	-3.23218300	-0.20789500
H	3.77492100	-1.19706200	-0.07177300
H	3.60819200	-2.50810800	-1.25555300
H	2.99773100	-0.87734500	-1.62729900
H	0.91538800	2.19210800	-0.65628500
H	1.12843300	0.71537600	1.25760400
H	2.36947400	0.46036200	0.04088500
H	0.36603800	-0.52008600	-2.45541800
H	-0.59413400	2.66877400	1.20507200
H	-2.98520300	2.72949100	1.90825300
H	-4.63811400	1.30900500	0.65707800
H	-3.81575700	-0.06719200	-1.23879400
H	-0.97873100	-1.85706300	0.17855100
H	-0.65160600	-0.67887900	1.45691400
H	-0.01915900	-2.33117800	1.59569200

16 References

[1] a) S. Mavila, B. T. Worrell, H. R. Culver, T. M. Goldman, C. Wang, C.-H. Lim, D. W. Domaille, S. Pattanayak, M. K. McBride, C. B. Musgrave, C. N. Bowman, *JACS* **2018**, *140*, 13594-13598; b) R. E. Thompson, B. Chan, L. Radom, K. A. Jolliffe, R. J. Payne, *Angew. Chem. Int. Ed.* **2013**, *52*, 9723-9727.
[2] N. J. Mitchell, L. R. Malins, X. Liu, R. E. Thompson, B. Chan, L. Radom, R. J. Payne, *JACS* **2015**, *137*, 14011-14014.
[3] C. Donald, S. Boyd, *Tetrahedron Lett.* **2012**, *53*, 3853-3856.
[4] G. te Velde, F. M. Bickelhaupt, E. J. Baerends, C. Fonseca Guerra, S. J. A. van Gisbergen, J. G. Snijders, T. Ziegler, *J. Comput. Chem.* **2001**, *22*, 931-967.
[5] M. Ernzerhof, G. E. Scuseria, *The Journal of Chemical Physics* **1999**, *110*, 5029-5036.
[6] E. Van Lenthe, E. J. Baerends, *J. Comput. Chem.* **2003**, *24*, 1142-1156.
[7] E. Caldeweyher, S. Ehlert, A. Hansen, H. Neugebauer, S. Spicher, C. Bannwarth, S. Grimme, *The Journal of Chemical Physics* **2019**, *150*, 154122.
[8] M. Reimann, M. Kaupp, *The Journal of Physical Chemistry A* **2020**, *124*, 7439-7452.
[9] J. Wang, R. M. Wolf, J. W. Caldwell, P. A. Kollman, D. A. Case, *J. Comput. Chem.* **2004**, *25*, 1157-1174.
[10] V. Sergiievskyi, G. Jeanmairet, M. Levesque, D. Borgis, *The Journal of Chemical Physics* **2015**, *143*, 184116.
[11] K. A. Peterson, T. B. Adler, H.-J. Werner, *The Journal of Chemical Physics* **2008**, *128*, 084102.
[12] F. Weigend, A. Köhn, C. Hättig, *The Journal of Chemical Physics* **2002**, *116*, 3175-3183.
[13] K. E. Yousaf, K. A. Peterson, *The Journal of Chemical Physics* **2008**, *129*, 184108.
